# Aggressive or moderate drug therapy for infectious diseases? Trade-offs between different treatment goals at the individual and population levels

**DOI:** 10.1371/journal.pcbi.1007223

**Published:** 2019-08-12

**Authors:** Jérémie Scire, Nathanaël Hozé, Hildegard Uecker

**Affiliations:** 1 Institute of Integrative Biology, ETH Zürich, Zürich, Switzerland; 2 Department of Biosystems Science and Engineering, ETH Zürich, Basel, Switzerland; 3 Swiss Institute of Bioinformatics, Lausanne, Switzerland; 4 Mathematical Modelling of Infectious Diseases Unit, Institut Pasteur, Paris, France; 5 Research group Stochastic Evolutionary Dynamics, Department of Evolutionary Theory, Max Planck Institute for Evolutionary Biology, Plön, Germany; Yale School of Public Health, UNITED STATES

## Abstract

Antimicrobial resistance is one of the major public health threats of the 21^st^ century. There is a pressing need to adopt more efficient treatment strategies in order to prevent the emergence and spread of resistant strains. The common approach is to treat patients with high drug doses, both to clear the infection quickly and to reduce the risk of *de novo* resistance. Recently, several studies have argued that, at least in some cases, low-dose treatments could be more suitable to reduce the within-host emergence of antimicrobial resistance. However, the choice of a drug dose may have consequences at the population level, which has received little attention so far. Here, we study the influence of the drug dose on resistance and disease management at the host and population levels. We develop a nested two-strain model and unravel trade-offs in treatment benefits between an individual and the community. We use several measures to evaluate the benefits of any dose choice. Two measures focus on the emergence of resistance, at the host level and at the population level. The other two focus on the overall treatment success: the outbreak probability and the disease burden. We find that different measures can suggest different dosing strategies. In particular, we identify situations where low doses minimize the risk of emergence of resistance at the individual level, while high or intermediate doses prove most beneficial to improve the treatment efficiency or even to reduce the risk of resistance in the population.

## Author summary

The obvious goals of antimicrobial drug therapy are rapid patient recovery and low disease prevalence in the population. However, achieving these goals is complicated by the rapid evolution and spread of antimicrobial resistance. A sustainable treatment strategy needs to account for the risk of resistance and keep it in check. One parameter of treatment is the drug dose, which can vary within certain limits. It has been proposed that lower doses may, in some cases, be more suitable than higher doses to reduce the risk of resistance evolution in any one patient. However, if lower doses prolong the period of infectiousness, such a strategy has consequences for the pathogen dynamics of both strains at the population level. Here, we set up a nested model of within-host and between-host dynamics for an acute self-limiting infection. We explore the consequences of drug dosing on several measures of treatment success: the risk of resistance at the individual and population levels, the outbreak probability and the disease burden of an epidemic. Our analysis shows that trade-offs may exist between optimal treatments under these various criteria. The criterion given most weight in the decision process ultimately depends on the disease and population under consideration.

## Introduction

Drug resistance is a rising source of concern for health organizations worldwide [1, 2]. The introduction of new antimicrobial agents is often followed closely or even preceded by the emergence of resistant strains, which threatens the efficiency of treatments [3, 4]. It is therefore necessary to adopt treatment strategies that successfully treat diseases while minimizing the risk of resistance.

One component of treatments that can be varied is the drug dose (for a review, see [5]). A lower bound is given by the minimal dose that provides a sufficient therapeutic effect. The upper limit for the dose is set to prevent toxic drug effects. Together, these two boundaries define the therapeutic window. For decades, the commonly accepted strategy has been to treat infections as harshly as possible, that is to choose the highest non-toxic dose [6, 7]. This choice is driven by the expectation that a high dose leads to a rapid eradication of the pathogen, enhancing the chances of survival and swift recovery of the patient. With respect to the evolution of resistance, a high dose could prove beneficial for two reasons. First, a rapid elimination of the sensitive strain reduces the number of new resistance mutations occurring during treatment. Second, for a sufficiently high dose—above the mutant prevention concentration [8]—resistance might be several mutational steps away, making the emergence of resistance considerably less likely. Also, if the dose is so high that resistance is physiologically impossible, no resistance problem exists.

The probability that resistance evolves does not only depend on the rate at which mutations appear but also on the establishment probability of the resistant strain. A high drug dose limits the rate of appearance. However, it might facilitate the proliferation of resistant mutants. The concern is that the elimination of the sensitive strain allows the resistant strain to spread more easily by releasing it from the pressure of competition. This is particularly risky if resistant types pre-exist prior to treatment. Moreover, the immune response might not be strongly triggered if the drug-sensitive pathogens get quickly cleared by the drug. Resistant pathogens are therefore not killed by the immune system while rare, potentially allowing them to reproduce and reach high numbers. These considerations have led several authors to question the standard “hit hard” strategy and to suggest that—at least under some circumstances—a milder treatment might be preferable [9–13]. Experimentally, the idea of low-dose treatment has received particular attention for the treatment of malaria infections [14–17]. For example, Huijben et al. (2013) find that for rodent malaria, low drug doses reduce resistance with no cost in terms of host health [14].

Combining all factors, the adopted schematic view is that the probability of resistance emergence follows an inverted U-curve [5, 18–26] (but more complicated shapes are conceivable): for very low doses, there is no selection for resistance, and for very high doses, resistance is either unlikely due to a large genetic barrier or even physiologically impossible. Kouyos et al. (2014) and Day and Read (2016) argue that, eventually, the range of the therapeutic window determines whether a harsh or a mild treatment is best to mitigate the evolution of resistance [5, 12].

An aspect that has received surprisingly little attention so far is the effect of dose on the disease dynamics in a community [5]. Virtually all studies on drug dosing focus on the evolution of resistance within a single patient (e.g. [10, 12, 14, 27–29]; for an exception see [30]). However, for infectious diseases, it is equally (or even more) important to constrain the transmission of resistance [31], and it is not clear whether a dose best at reducing the probability of resistance within a single host is optimal for managing the disease in the population.

Population level considerations resemble those at the within-host level. Again, two factors are crucial for the evolution of resistance: the total rate at which patients develop a resistant infection, and the probability that an existing resistant strain spreads across the community. With a mild treatment, the infection duration may be prolonged, increasing the risk to infect another member of the community and hence ultimately the total number of infections. Each of these infections might, in turn, become resistant. It is therefore conceivable that even if a mild treatment reduces the risk of *de novo* resistance in every single patient, it increases the rate of resistance appearance in the population. On the other hand, a high dose treatment could lead to a competitive release effect at the between-host level, allowing the resistant strain to spread more easily.

Colijn and Cohen (2015) study the effect of the dose on the level of resistance both at the within- and between-host levels [30]. Performing a parameter sensitivity analysis, they show that at both scales, competition between strains is indeed crucial for whether a mild or a harsh treatment reduces levels of resistance. Importantly, they also discuss the possibility of trade-offs in optimizing the drug dose to minimize resistance at the two levels. However, in their between-host model, the within-host probability of resistance and the patient recovery rates are independent parameters, while in reality they are coupled with each other through the dynamics at the within-host level. Within this framework, it is therefore not possible to explore if and when trade-offs exist within the accessible range of the coupled epidemiological parameters.

In this article, to avoid this limitation, we set up a nested model, taking into account both the dynamics within patients and the spread of the disease between individuals.

Resistance mutations can appear *de novo* within patients and can also be transmitted between individuals. For a more intuitive understanding of the results, we complementarily analyse a standard epidemiological model, for which we estimate the parameters from stochastic within-host simulations. To determine optimal dosing, we apply different measures of treatment success: (1) emergence of resistance at the individual and (2) at the population level, (3) the outbreak probability of an epidemic, and (4) the total disease burden in the population, given an outbreak occurs. We find that, in many cases, the choice of a treatment strategy entails trade-offs between these different criteria. For instance, a low dose treatment might minimize the probability of emergence of resistance at the within-host level but maximize transmission of resistance at the between-host level. This demonstrates the importance of considering both scales and several criteria for determining optimal dosing.

## Methods

### The model

Treatment of mild self-limiting infections is considered by some to be one of the main drivers of the spread of resistance in the community [32]. For this reason, we model an acute self-limiting disease, such as acute otitis media, strep throat or tonsillitis, where the immune system is able to clear the infection even in the absence of the drug [13, 32]. Drug treatment speeds up recovery and reduces the infectious period.

#### Within-host dynamics

For the within-host part, we adopt the two-strain model from Day and Read, 2016 [12] with minor changes. The pathogen load inside the host is denoted by *P*_*w*_ and *P*_*m*_ for the wild-type sensitive strain and the mutant resistant strain, respectively. Mutations happen in both directions during pathogen replication with probability *μ*. (Relaxing the often made assumption that back mutation is not possible does not affect the conclusions; the primary effect of mutation is to seed resistant pathogens into a system that is initially dominated by the sensitive strain). The drug acts by reducing the replication rate of the pathogens, and the replication rates of each strain, *r*_*w*_(*c*) and *r*_*m*_(*c*), are decreasing functions of the drug concentration *c*, where the resistant strain can withstand considerably higher drug pressure than the sensitive strain. The drug concentration is assumed to be constant during the entire course of treatment. The resistance mutation entails a cost, manifested in a lower replication rate in the absence of the drug, (*r*_*w*_(0) ≥ *r*_*m*_(0)). Pathogens of both genotypes have an intrinsic death rate *η*.

The immune response, *I*, is triggered proportionally to the total pathogen load (*P*_*w*_ + *P*_*m*_) with a factor λ and decays at a constant rate *δ*. The depletion of pathogens due to the immune system follows mass-action kinetics and leads to additional pathogen death rates *κP*_*w*_*I* and *κP*_*m*_*I*. Both strains are hence equally affected by the immune system, and there is full cross-immunity.

Deterministically, the dynamics can be described by the following set of differential equations:

$$\dot{P_{w}}=\left(r_{w}\left(c\right)\;\left(1\;-\;\mu \right)-\;\eta \right)\;P_{w}\;-\;\kappa \;P_{w}\;I\;+\;r_{m}\left(c\right)\;\mu \;P_{m}$$ (1a)

$${\dot{P}}_{m}=\left(r_{m}\left(c\right)\;\left(1\;-\;\mu \right)-\;\eta \right)\;P_{m}\;-\;\kappa \;P_{m}\;I\;+\;r_{w}\left(c\right)\;\mu \;P_{w}$$ (1b)

$$\dot{I}=\lambda \;(P_{w}\;+\;P_{m})\;-\;\delta \;I.$$ (1c)

The explicit formulas for the replication rates *r*_*w*_ and *r*_*m*_ are given by:

$$r_{w}\left(c\right)=\frac{\epsilon }{2}\;\left(1-\tanh\left(\;\omega \;\left(c-\theta_{w}\right)\right)\right)$$ (2a)

$$r_{m}\left(c\right)=\frac{\epsilon \;(1-\alpha )}{2}\;\left(1-\tanh\left(\;\omega \;\left(c-\theta_{m}\right)\right)\right),$$ (2b)

where *ϵ* is the growth rate of the susceptible strain in the absence of treatment, *α* represents the fitness cost of resistance, *θ*_*w*_ (*θ*_*m*_) is the dose for which the growth rate of the sensitive (resistant) strain is half the growth rate in the absence of treatment and *ω* determines the steepness of the dose response curve. Resistance is characterized by *θ*_*m*_ > *θ*_*w*_ and *α* ≥ 0.

**Fig 1:**
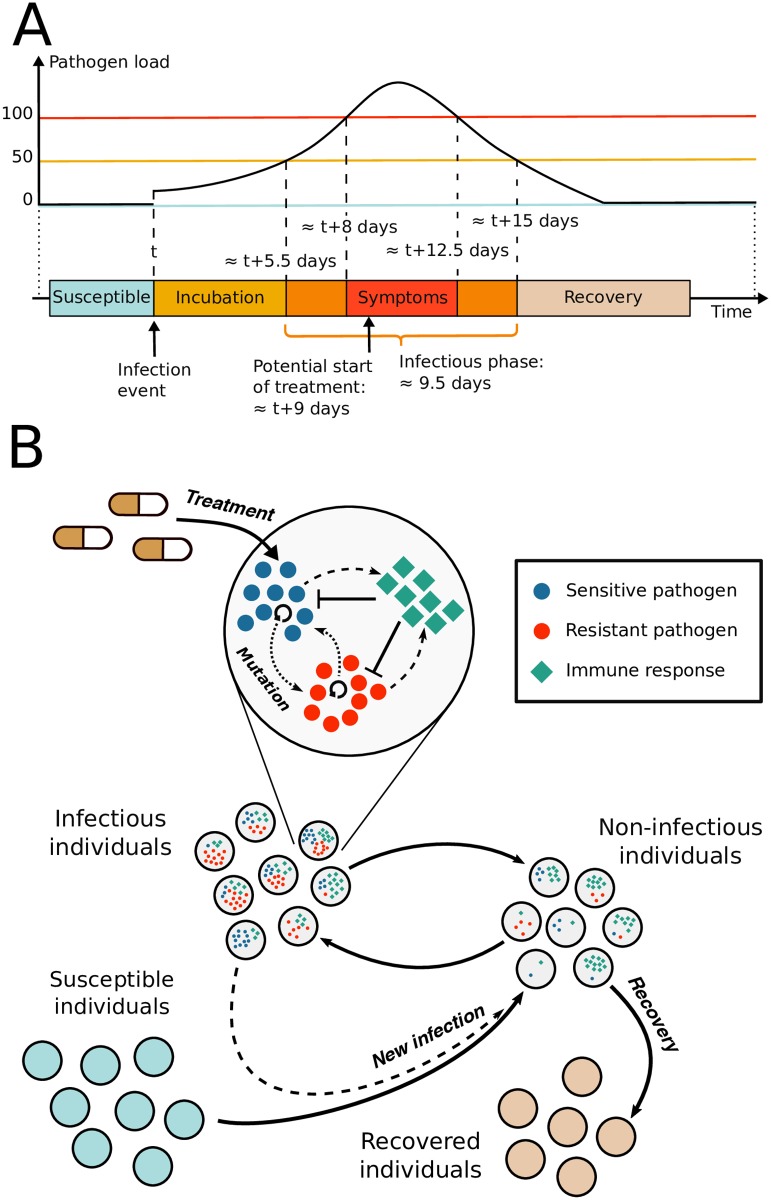
Nested model of an acute infectious disease. (A) Average timeline of the infection for a non-treated individual. The host’s transition from one phase to the other is determined by the pathogen load. (B) Diagram of the nested model. Prior to infection, individuals are susceptible. For infected individuals, the pathogen load defines if an individual is infectious or non-infectious. Once the pathogen load has dropped to zero, individuals are recovered and have aquired life-long immunity.

Fig 1A shows the timeline of an average infection. A susceptible host gets initially infected by a small number of pathogens (at the same time, we choose this number high enough to make stochastic extinction unlikely; concretely, it is set to 7 pathogens). The pathogen load then increases during a symptom-free incubation period. Once the pathogen load crosses a threshold of 50 pathogens, the infected individual turns infectious, and once the pathogen load crosses a threshold of 100 pathogens, symptoms set in (for computational ease, we work with small population sizes as has been done in [12]). Treatment is applied after a delay of one day, which subsumes all steps between the appearance of symptoms and the moment when treatment starts being effective. Due to treatment and to the patient’s immune response, the infection eventually recedes and the patient goes from being infectious to non-infectious as the pathogen load decreases below the infectivity threshold (set to 50 units, see above). The drug is administered to the patient until the pathogen population is extinct. Once the pathogen load of an infected individual has dropped to zero, the patient is considered as recovered and is assumed to have acquired life-long immunity to the disease due to immunological memory, which we do not model explicitly.

For the main text, we choose the parameters such that in a typical course of infection, patients become infectious about 5.5 days after infection, and develop symptoms 2.5 days later. For all figures in the main text, we set *μ* = 0.002, *η* = 0.02 day^−1^, *κ* = 0.0225 day^−1^, λ = 0.035 day^−1^, *δ* = 0.01 day^−1^, *ϵ* = 0.6 day^−1^, *α* = 0.017, *θ*_*w*_ = 0.3, *θ*_*m*_ = 0.6 and *ω* = 15. The initial level of the immune system is set to *I*_*t*=0_ = 7. With these parameters, the disease is self-limiting, i.e., even in the absence of treatment, the infection is successfully suppressed by the host’s immune system.

#### Between-host dynamics

On top of this within-host model, we build a between-host model, describing the spread of the disease in a population (see Fig 1). Initially, a single individual is infected with the sensitive strain, and all other individuals are susceptible. How well the infection spreads is determined by the basic reproductive number, defined as the mean number of secondary cases caused by one infectious host in an otherwise fully susceptible population. This number crucially depends on the length of the infectious period and thereby on the drug dose. In the following, we denote by *R*_0_ the basic reproductive number of the sensitive strain in the absence of treatment.

For simplicity, we assume that chances of transmission depend on the infecting host’s pathogen load in a step-function fashion (no transmission below a certain threshold, full transmission above). Other than that, all infectious individuals are equally likely to infect another population member, independent of the genetic composition of the pathogen population and treatment status. There is therefore no direct transmission cost of the resistant strain. All individuals (infected or not) except recovered ones are potential targets for infection, and infection occurs randomly between individuals, i.e. we disregard population structure. With *I* the number of infectious hosts, *R* the number of recovered individuals, and *β* the transmission coefficient, the rate at which infection events happen in the population is given by *β* × *I* × (*N* − *R* − 1). (The “−1” accounts for the fact that infectious individuals do not infect themselves).

The inoculum size at infection is fixed to 7 pathogens and again independent of the infecting host’s pathogen load. If the infecting host is co-infected by both pathogen strains, the transmitted strain is chosen randomly in proportion to the pathogen load of each strain in this host. Only one strain is therefore transmitted at each infection event. This relies on the idea that the two pathogen strains are not well mixed in a co-infected individual and that pathogens in close spatial proximity are likely to be transmitted together, which in turn makes it likely that all pathogens in the inoculum derive from the same clone. Superinfection of an already infected individual follows the same rules as infection of a pathogen-free individual.

Since the infection is assumed to be non-lethal and since the duration of the epidemic outbreak is short for the vast majority of parameter sets used in this study (in the order of 10^2^ days), we ignore birth and death of individuals as well as immigration into or emigration from the population. The total population size is set to *S*_0_ = 10, 000 throughout the manuscript. Within this framework, the epidemic occurs in one “wave” and then goes extinct. Even for high transmission coefficients, persistence is impossible since, as the epidemic proceeds, the number of susceptible hosts drops to a level too low to maintain the epidemic. For low transmission coefficients, for which the average number of secondary cases is less than one, most outbreaks will be small. For high transmission coefficients that are large enough for the epidemic to spread, the distribution of outbreak sizes is bi-modal. Either the epidemic comes to a halt quickly due to stochastic extinction, or a large outbreak occurs that is only stopped by the lack of a sufficient number of susceptible hosts (see Fig I).

### Analysis

#### Agent-based simulations

We run stochastic simulations of the nested model of within-host and between-host dynamics. The differential equations of the within-host level (Eq 1) and the state-transition rules for the between-host level are implemented stochastically, using the fixed-step-size tau-leaping Gillespie algorithm [33, 34]. Simulations start at time *t* = 0 with one individual newly infected with the wild-type strain of the pathogen. At each time step, the within-host infection dynamics of all the infected individuals are updated, using the stochastic counterpart of Eq 1 with exponentially distributed waiting times. At the end of each step, transmission events (new infections and superinfections) are randomly drawn, using the rules defined in the previous paragraph. Simulation runs end when there is no pathogen-carrying host in the population anymore. In all simulations presented here, the time steps of the algorithm are of length *δ*_*t*_ = 5 × 10^−3^ days. The implementation is done in Java. For each parameter set explored, we run enough independent simulations of the dynamics to capture the overall distribution of possible outcomes with reasonably-high confidence. There is a high variance in complexity of these individual simulations, depending on how well the pathogen is spreading in the population, which leads to a portion of runs finishing very fast and some taking much longer to run.

#### Simplification into a deterministic SIR model

The agent-based nested model is complex to analyze and to dissect. To gain a better and more intuitive understanding, we introduce a classical epidemiological model, representing a simplified version of the dynamics. Except for the transmission coefficient *β*, all the parameters of this simplified model are extracted from simulations of the stochastic within-host dynamics of the nested model.

**Fig 2:**
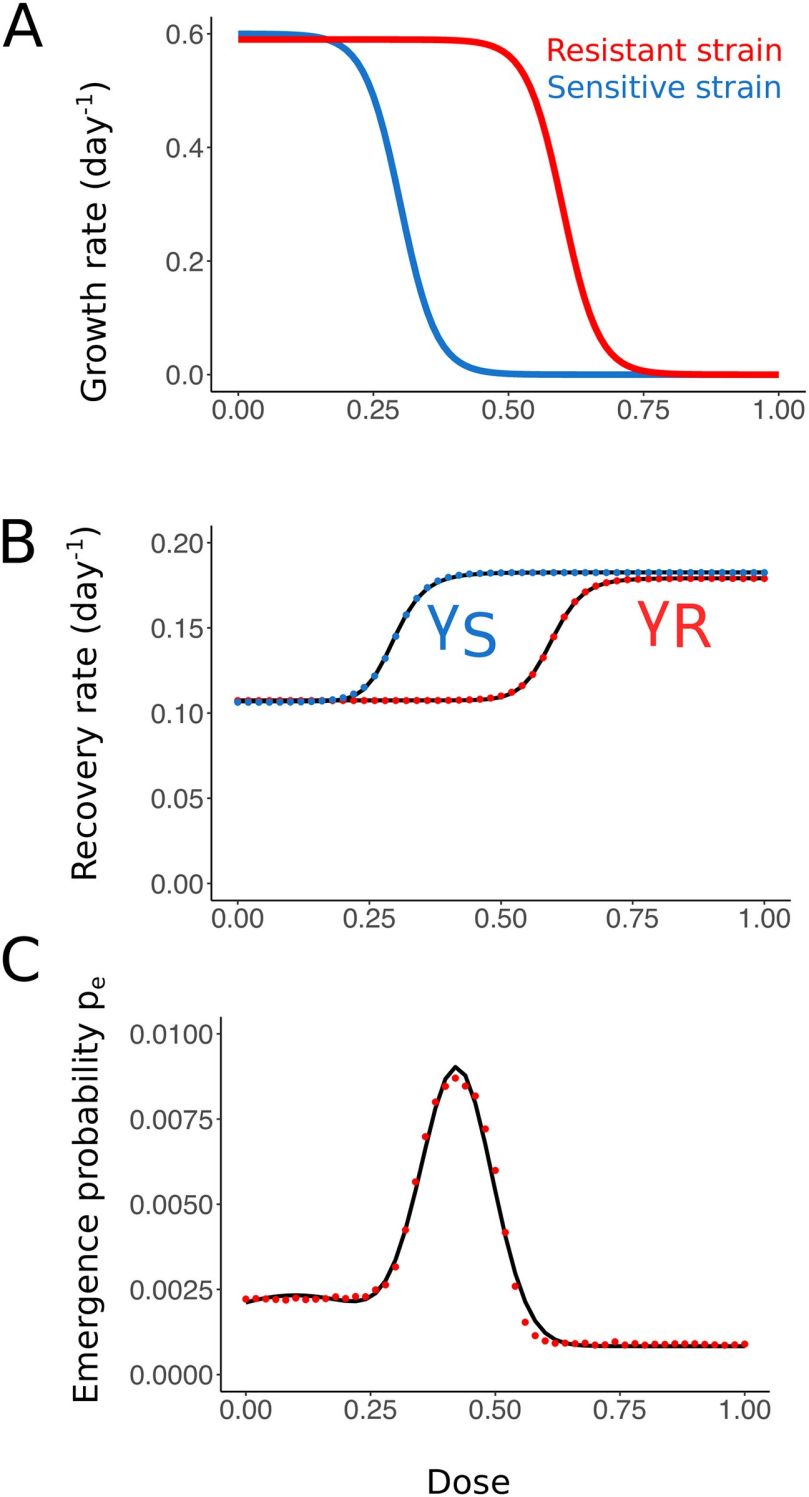
Transition from the nested agent-based model to an SIR model. (A) Effect of treatment strength on the growth rate for the sensitive and resistant strains. At very high doses, neither strain can grow in the presence of treatment. (B) Mean recovery rates of an individual infected by the sensitive (blue) and resistant (red) strain. Simulated results are fitted with sigmoid functions. (C) Probability of emergence of resistance as a function of dose, obtained from 2 × 10^6^ within-host simulations per dose (red) and fitted with the sum of two Gaussians (black).

For the simplified deterministic model, we divide patients into four groups. Individuals can be either susceptible, infected by one of the two strains, or recovered (SIR model, see the schematic Fig III). The relevant processes, leading to transitions between the compartments, are infection, patient recovery, and the emergence of resistance. Unlike the emergence of resistance, reversion of drug-resistant to drug-sensitive infections can be ignored since sensitive infections initially dominate and later, the dynamics are governed by the competition for hosts. We describe the dynamics by a set of deterministic differential equations:

$$\dot{S}=-\;\beta \;S\;(I_{S}+I_{R}),$$ (3a)

$${\dot{I}}_{S}=\beta \;S\;I_{S}-\gamma_{S}\left(c\right)\;I_{S},$$ (3b)

$${\dot{I}}_{R}=p_{e}\left(c\right)\;\gamma_{S}\left(c\right)\;I_{S}\;+\;\beta \;S\;I_{R}\;-\;\gamma_{R}\left(c\right)\;I_{R},$$ (3c)

$$\dot{R}=\left(1\;-\;p_{e}\left(c\right)\right)\;\gamma_{S}\left(c\right)\;I_{S}\;+\;\gamma_{R}\left(c\right)\;I_{R},$$ (3d)

where *S* is the compartment of susceptible hosts, *I*_*S*_ and *I*_*R*_ the compartments of infected hosts, respectively with the sensitive and resistant strain, and *R* is the compartment of recovered individuals. As before, *β* is the transmission coefficient of the pathogen, assumed identical for both strains. *γ*_*S*_(*c*) and *γ*_*R*_(*c*) are the dose-dependent recovery rates of individuals infected with the sensitive strain and the resistant strain, and *p*_*e*_(*c*) is the probability of resistance emergence at the within-host level. We estimate the functions *γ*_*S*_(*c*), *γ*_*R*_(*c*), and *p*_*e*_(*c*) from simulations of the within-host model introduced above (Fig 2). Details are given in SI section S3.1.

The SIR model provides a deterministic non-nested approximation of the full agent-based model. It only describes the between-host dynamics and therefore does not allow for feedbacks between the within-host and between-host levels. While differing in many respects from the agent-based model, the SIR model is still able to capture important aspects of the dynamics such as competition of the two pathogen strains for susceptible hosts at the population level. The non-nested SIR model helps us to explore parameter ranges more easily than with the computationally expensive nested model. Especially, it allows us to efficiently investigate the influence of the coefficient of transmission of the epidemic *β*, which is shared between the two models. In the SIR model, infections are clearly classified as caused by the sensitive or the resistant strain and mixed infections (as they naturally appear in the nested model) are ignored. This simplification provides very helpful insight into the interaction of the two pathogen strains at the between-host level.

While we focus on the deterministic SIR model in the main text, we also discuss the relation of the agent-based model to the stochastic SIR model (i.e. the stochastic version of Eq 3) in SI section S3.4 and use it to gain additional insight in SI section S5.

For the SIR model, the basic reproductive number (i.e. the number of secondary infections caused by one infected individual in a fully susceptible population) of the sensitive strain in the absence of treatment can be obtained as *R*_0_ = *βN*/*γ*_*S*_(0). As *R*_0_ is a very informative quantity in epidemiology—in particular, an epidemic can spread with non-zero probability for *R*_0_ > 1 and quickly goes extinct otherwise—, we will in the following report this *R*_0_, when we vary *β*. In the analysis presented here, the “true” basic reproductive number in the agent-based model hardly differs from the basic reproductive numbers calculated for the SIR model (Fig IV). (However, the distribution of the number of secondary infections differs between the full nested model and the stochastic SIR model (Fig VI)).

### Evaluating the success of a treatment strategy

Various criteria can be used to assess the quality of a treatment strategy, which—in our context—refers to the choice of drug dose. We use here four different measures. The first two focus on the evolution and spread of resistance, while the other two consider overall treatment success.

#### Probability of emergence of resistance at the within-host level

Focusing on the evolution of resistance, one aim is to minimize the probability of *de novo* emergence of resistance within a host, *p*_*e*_, as is done by Day and Read (2016) [12]. We say that resistance has emerged if the resistant pathogen load crosses a given threshold. We choose this threshold to be 50 pathogens. This corresponds to the threshold above which a host becomes infectious. Setting the threshold to 100 resistant pathogens (threshold for the appearance of symptoms) as was done by Day and Read (2016) with quite similar parameters does not affect the conclusions (Fig XXI).

#### Spread of resistance at the between-host level

As a measure of the risk of emergence and spread of resistance in the population, we count the number of transmission events of the resistant strain from one host to another (remember that only a single strain is transmitted at infection). These transmission events can either be superinfections of already-infected individuals or infections of susceptible hosts. Restricting this measure to infection of susceptible-hosts-only yields qualitatively similar results.

The other two criteria are based on the disease burden, which we define as the cumulative number of days that members of the population spend being infectious. With *δ*_*t*_ the step-size of the tau-leaping algorithm, and *I*(*t*_*i*_) the number of infectious hosts in the *i*^*th*^ calculation step, and *K* the number of steps of the algorithm needed to reach the disease-free equilibrium, *B*_0_ is approximated as:

$$B_{0}\approx \sum_{i=1}^{K}\;I\left(t_{i}\right)\;\delta_{t}.$$ (4)

We focus on infectious individuals rather than individuals showing symptoms to allow for a comparison of results from the agent-based model and the SIR model. As discussed below, this does not alter the conclusions.

#### Probability of an epidemic outbreak

Initially, only a single individual is infected. It is therefore possible that due to stochasticity, the epidemic dies out after only a few infection events rather than developing into a serious outbreak with many infecteds. We use the probability of an epidemic outbreak as a first measure of success of a chosen drug dose. To evaluate concretely if an epidemic has broken out in a simulation run, we focus on the disease burden *B*_0_.

As pointed out above and shown in Fig I, the distribution of *B*_0_ is bimodal unless the transmission coefficient is low and the drug dose high. In the vast majority of cases, a threshold of 300 patient days clearly separates epidemics that quickly went extinct due to stochasticity from serious outbreaks. Thus, we define the outbreak probability as the probability that the disease burden reaches at least 300 patient days and determine it as the fraction of simulation runs which yield a disease burden larger than the 300-patient-days threshold (estimating the outbreak probability from the number of secondary cases leads to very similar results, see section S2 and Fig II). Focusing on symptomatic or infectious individuals does not change the results on the outbreak probability.

#### Total disease burden over the course of an epidemic

As a fourth criterion, we assess the severity and magnitude of spread of an epidemic measured by the disease burden, provided an outbreak occurs, i.e. we only consider epidemics where the disease burden reaches at least 300 patient days. We denote the average disease burden in this subset of epidemics by *B*. This provides a measure of the overall negative impact that an epidemic has on the population. In the following, when we speak about the disease burden, we mean *B* (rather than *B*_0_).

In the SIR framework, the disease burden is given by

$$B=\;\int_{0}^{\infty }(I_{S}\left(t\right)+I_{R}\left(t\right))\;dt.$$ (5)

Note that since the SIR model is deterministic, the disease certainly spreads when one of the strains has a basic reproductive number >1, while the number of the infecteds cannot increase at all otherwise. We therefore do not include any conditioning.

## Results

### Within-host probability of resistance emergence

**Fig 3:**
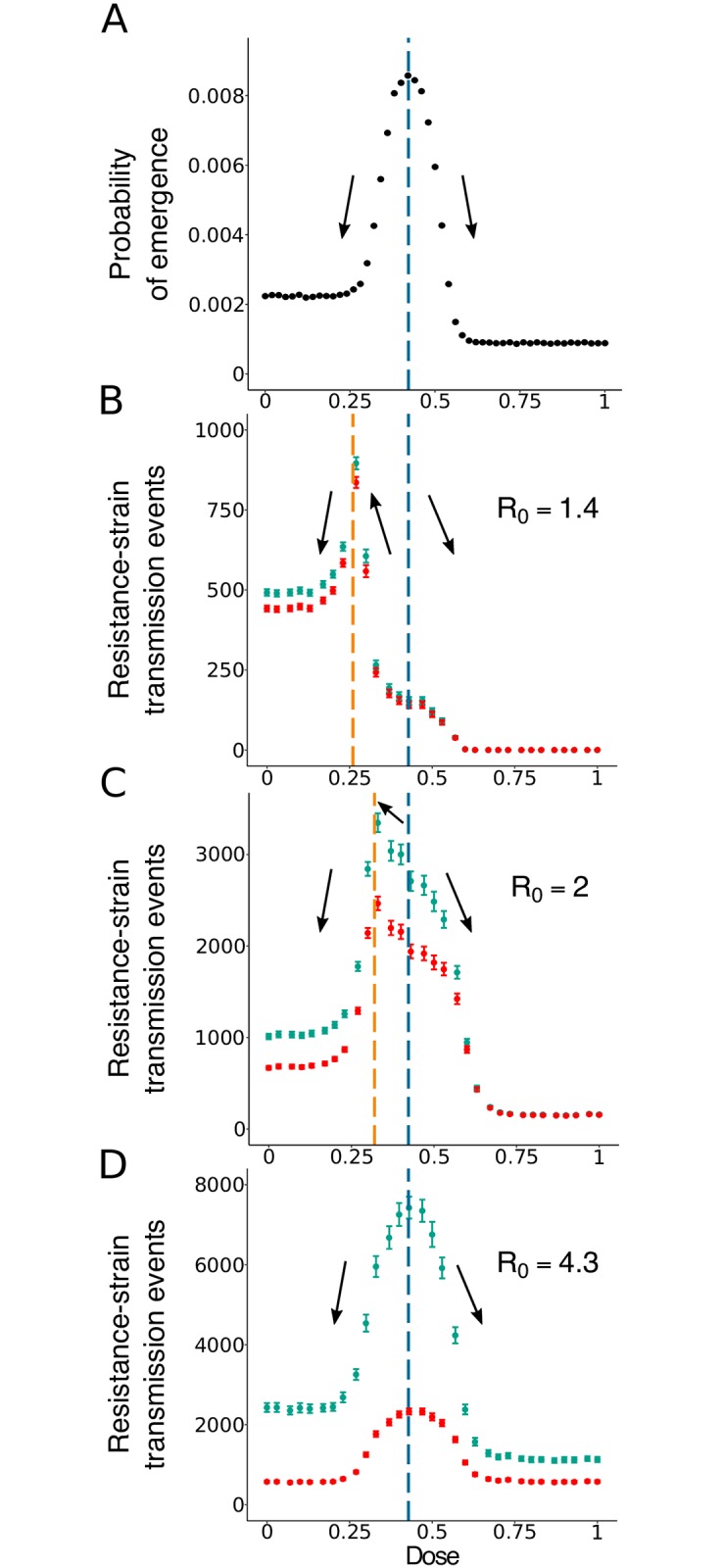
Comparison of optimal dose determined through the within-host probability of resistance (A) or through the number of transmission events of the resistant strain during an epidemic (B-D). Blue symbols show the total number of transmission events, while red symbols show only transmission events towards susceptible hosts. The vertical blue line corresponds to the peak in the within-host probability of resistance and the orange line to the peak in the number of resistant-strain transmission events. The black arrows represent the trends in the number of resistant strain transmission events when one shifts the treatment dose away from the dose giving a maximum in *p*_*e*_ (given by the vertical blue line). Mean over 1, 500 to 24, 000 simulations runs per data point. In dark blue and dark red: the 95% confidence intervals of the mean for each dose.

The probability that resistance evolves *de novo* within a single patient, *p*_*e*_(*c*), follows an inverted-U-shaped curve with a single maximum (see Fig 3A and [12]). For low doses, the sensitive strain triggers a strong immune response, and resistant pathogens are likely to be suppressed by the immune system before reaching high levels. For high doses, resistance is not sufficient to withstand the drug. The maximum corresponds to doses for which the sensitive strain is cleared too rapidly by the drug to evoke a strong immune response, while the resistant strain is not substantially affected by the drug. At these doses, spread of the resistant strain is easiest. The location of the peak is also influenced by the number of *de novo* mutations during treatment, which is higher with lower doses. Yet, for the chosen parameter set, this does not have a major effect. Changing the model parameters, we find that this statement is robust to variation in single parameters (see Fig XX). Only for a poor immune response (small λ, small *κ*, or large *δ*) does the peak shift to lower doses.

The dose at which the within-host probability of resistance is maximal remains the same if we set the threshold for the emergence of resistance to 100 (instead of 50) resistant pathogens (Fig XXI), and is then also robust to variation in single parameters (Fig XXII).

The best dose choice to reduce the within-host probability of emergence is not straightforward. Depending on the therapeutic window (i.e. on the range of doses that can practically be administered), the best dose choice can either be on the left-hand side of the central peak, in the low-dose region; or it can be on the right-hand side in the high-dose one. In any case, as highlighted by Day and Read [12], the optimal dose with respect to this criterion can be nothing else than one of the two boundaries of the therapeutic window.

### Transmission of the resistant strain in the population

We next explore how the expected number of transmission events of the resistant strain in the population, ${\bar{T}}_{R}$, depends on the drug dose. ${\bar{T}}_{R}$ is highest for intermediate doses but the location of the peak depends on the transmission coefficient *β* (Fig 3B–3D). This holds true if we vary single parameters of the within-host model (see Fig XXIII). Likewise, for a given *β*, the location of the peak depends on the population size *S*_0_ (Fig XB-D and Fig XXIII). What explains the difference in the location of the peak for low and high *R*_0_?

The number of transmission events of the resistant strain, *T*_*R*_, is influenced by two factors: the rate at which the resistant strain appears in the population (i.e. by the rate at which some host develops a resistant infection) and its chances to spread once it has appeared.

These two factors need not peak simultaneously. The rate at which the resistant strain appears in the population is determined by how likely the resistant strain is to emerge within a host (approximately given by *p*_*e*_) and by the number of hosts infected with the wild-type strain. The more individuals are infected by the wild-type pathogen, the more chances the resistant strain has to emerge.

On the other hand, large wild-type outbreaks that favor the *de novo* appearance of the resistant strain in the population do not provide ideal conditions for its spread. For large wild-type outbreaks, by the time the resistant strain appears, many individuals have already acquired immunity through infection with the sensitive strain. At low doses, where the sensitive strain is able to infect many individuals, the spread of the resistant strain is hence hindered through the greatly reduced pool of targets of infection and through competition with the sensitive strain for the remaining susceptibles. However, at high doses, it cannot spread well either since it has an equally low fitness as the sensitive strain. Spread of the resistant strain is hence expected to be easiest at intermediate drug doses, where it experiences competitive release from the sensitive strain and is itself barely affected by treatment. Indeed, it seems intuitive that spread of resistance at the between-host level is easiest at similar doses as the spread of the resistant strain at the within-host level (which largely determines the peak in *p*_*e*_ as discussed above): the differential recovery rates for patients who are infected with the sensitive or the resistant strain reflect the differential growth rates at the within-host level (compare Fig 2A and 2B); the former strongly affect the spread of the resistant strain at the population level, the latter at the within-host level.

For a high *R*_0_, a large initial epidemic of the wild-type pathogen is likely even for high drug doses. A large wild-type epidemic ensures that the resistant strain appears in some hosts in the population (though the rate of appearance of resistance is, of course, still modulated by the within-host probability of resistance). Therefore, for high *R*_0_, the shape of ${\bar{T}}_{R}$ is largely dominated by the rate of spread (Fig 3D and Fig XD), which, as pointed out in the previous paragraph, peaks at similar doses as *p*_*e*_ such that these two factors do not have any antagonistic effects. In contrast, for low *R*_0_, appearance of resistance at the population level is a limiting factor. For low *R*_0_, the probability of a large outbreak (with a large opportunity for resistance to appear) drops strongly with the drug dose, shifting the peak to lower doses, where large outbreaks are more likely (Fig 3B and Fig XD). If we consider transmission events towards susceptibles only (red symbols in Fig 3), the same conclusions for the location of the peak hold. (However, the peak is then highest for intermediate *R*_0_, presumably because for high *R*_0_, too few targets of infection are available). Note that the shift in the peak is primarily due to stochasticity in the appearance of resistance. This includes in particular that resistance might not appear at all before extinction of the disease. In the classical epidemic model with susceptible, infected, and recovered hosts (SIR model, see Methods section), the integral $\int_{0}^{\infty }\beta I_{R}\left(t\right)S\left(t\right)dt$ quantifies transmission of the resistant strain (there is no superinfection in the SIR model and all transmission is towards susceptibles). Appearance of the resistant strain is guaranteed in this setting, and the location of the peak depends only weakly on *R*_0_.

The basic reproductive number *R*_0_ does not only depend on *β* and *S*_0_ but also on the recovery rate in the absence of treatment, *γ*_*S*_(0). From this, we would expect a shift in the peak to lower doses for large *γ*_*S*_(0) (for a given *β* and *S*_0_). However, changes in *γ*_*S*_(0) go hand in hand with changes in the recovery rates at other doses. In particular, the ratio of *γ*_*S*_(0) and *γ*_*S*_(1) will change (see Fig XVIII), and this has consequences for the location of the peak as well. If treatment has a large effect on the recovery rate (*γ*_*S*_(0)/*γ*_*S*_(1) small), the difference in the number of sensitively infected patients between low-dose and high-dose treatment is large. Hence for a given *R*_0_, the peak shifts to lower doses with a higher drug efficacy (i.e. higher *γ*_*S*_(1)). One parameter of the within-host model that strongly affects *γ*_*S*_(1) but not *γ*_*S*_(0) is the pathogen threshold for the onset of symptoms (see Fig XIX). With a lower symptoms threshold, onset of treatment is earlier and recovery is faster. For fixed *R*_0_, the peak in ${\bar{T}}_{R}$ therefore shifts to lower doses for a lower symptom threshold as shown in Fig X.

In section S5, we analyze an endemic rather than an epidemic disease, modeled by an SIR model with immigration/birth and death. We assume that the population is at endemic equilibrium prior to the evolution of resistance and evaluate the risk of resistance emergence. The model allows for an analytical treatment, making it easy to disentangle the two factors contributing to resistance (rate of appearance and spread). An analysis of the equilibrium number of infecteds in the absence and presence of drug treatment supports the statement that the location of the peak depends on *R*_0_ as well as on the treatment efficacy (i.e. the ratio of *γ*_*S*_(0) and *γ*_*S*_(1)).

### Trade-offs between the risk of resistance at the within-host and between-host levels

For increasing *R*_0_ (Fig 3C and 3D and Fig XC,D), the peak in the number of transmission events of the resistant strain converges to similar doses as the peak in *p*_*e*_. This means that, for high *R*_0_, decisions made to minimize *p*_*e*_ will also minimize the number of events of transmission of the resistant strain and vice versa. In contrast, with a rather low *R*_0_ (Fig 3B and Fig XB), the peak in the number of transmission events of the resistant strain is considerably shifted towards lower doses compared to the peak in the probability of within-host emergence of resistance, *p*_*e*_ (shown in Fig 3A). Therefore, there is a non-negligible range of doses for which lowering the dose in order to reduce the probability of within-host emergence of resistance will in return substantially increase the average number of transmission events of the resistant strain. For many if not all strategies for resistance mitigation, this would be the exact opposite of the aimed outcome.

Likewise, for a given *R*_0_, we find a trade-off between reducing the risk of resistance at the within-host and between-host levels if treatment has a strong effect on recovery but both measures align for a less strong effect (see Fig X).

In all instances of a trade-off as discussed here, the risk of resistance in the population peaks for considerably lower doses than the within-host emergence of resistance. In the case of a poor immune system (where *p*_*e*_(*c*) peaks at lower doses as mentioned above), the opposite trade-off can occur with *p*_*e*_(*c*) being maximal at lower doses than ${\bar{T}}_{R}$ (Fig XI).

For the endemic model, we find similar patterns in the risk of resistance as for the epidemic disease considered in the main text (Fig VII). This also holds if immunity is not life-long but lost at a certain rate (Fig VIII).

### Outbreak probability

**Fig 4:**
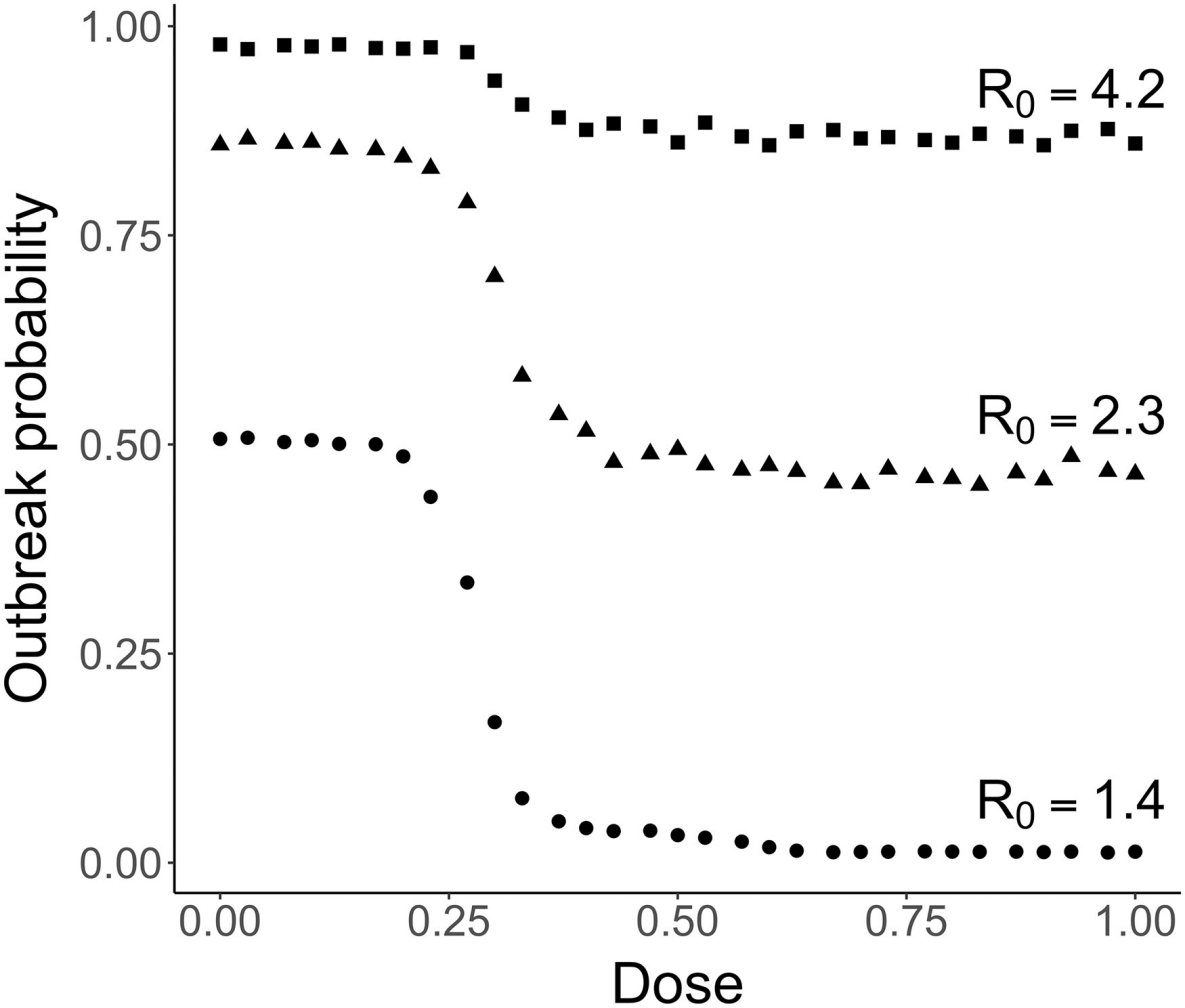
Effect of the treatment strength on the outbreak probability for various transmission coefficients. Mean over 1500 to 20000 simulations per dose and transmission coefficient. Circles: *R*_0_ = 1.4 (*β* = 1.5 × 10^−5^ days^−1^). Triangles: *R*_0_ = 2.3 (*β* = 2.5 × 10^−5^ days^−1^). Squares: *R*_0_ = 4.2 (*β* = 4.5 × 10^−5^ days^−1^). The confidence intervals are not shown as they are too small to be clearly seen on the plot.

Initially, only a single individual is infected. It is therefore possible that by chance, the disease disappears from the population after just a few infection events. If it survives this initial phase, the population is hit by an epidemic outbreak. (This dichotomic behavior holds unless *R*_0_ is small; see Methods section). The stronger the dose administered to the infected patients in the population, the less likely an outbreak is to occur (Fig 4 and Fig XXIV). The first few infected individuals are very likely to be mostly carrying the sensitive strain. A stronger dose decreases the time during which these individuals are infectious, making them less likely to transmit the pathogen further in the population (cf. also SI section S2). This in turn reduces the outbreak probability. Thus, the increased recovery rate in early-infected patients associated with an increased treatment strength is key in reducing the likelihood that a budding epidemic can develop further. Therefore, to reduce the outbreak probability, one should prescribe the highest available dose of treatment to early-infected patients. As can be seen in Fig 4, the relative reduction in the outbreak probability for high compared to low doses is stronger for low than for high *R*_0_.

These considerations can be related to the outbreak probability in a one-strain stochastic SIR model, given by $P_{\text{outbreak}}\left(c\right)=1-1/R_{0}\left(c\right)=1-\frac{\gamma_{S}\left(c\right)}{\beta S_{0}}$ [35, p. 107]. Here, *γ*_*S*_(*c*) is the dose-dependent recovery rate for infections with the sensitive strain, *β* is the transmission coefficient as before, and *S*_0_ denotes the initial number of susceptible hosts. *P*_outbreak_ shows the same qualitative behavior as observed in Fig 4. However, since the distribution of secondary cases differs between the stochastic SIR model and the agent-based model, the outbreak probabilities differ quantitatively by a large margin (see SI sections S3.4.1 and S3.4.2).

### Disease burden in large outbreaks

Last, we consider the disease burden, defined here as an epidemiological measure of the total number of days that individuals spend being infectious (in other words, it can be thought of as (Number of infected people) × (Duration of an infection), see Methods section for details). We focus on the disease burden, provided a large outbreak occurs, and denote this quantity by *B*.

**Fig 5:**
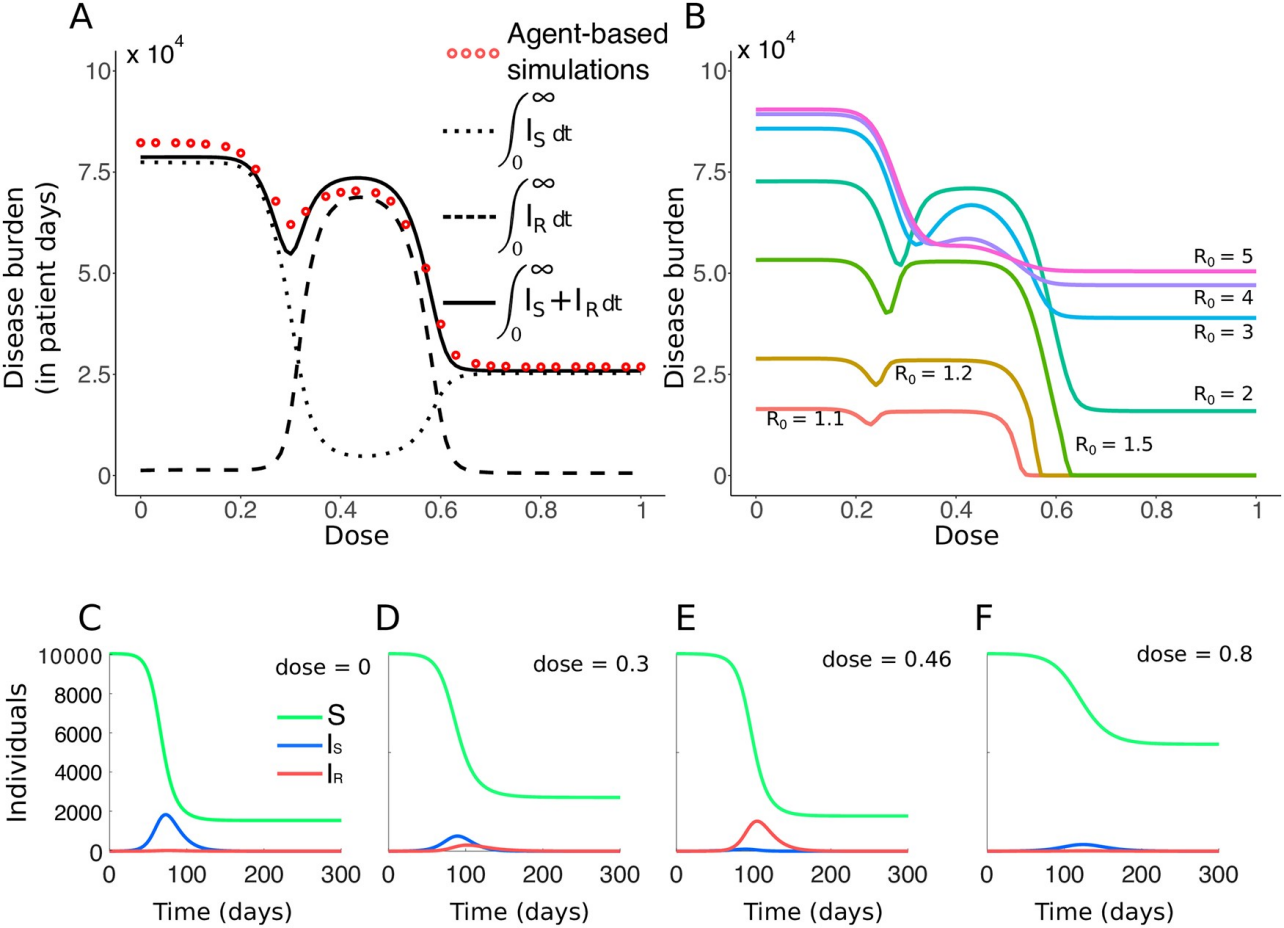
Effect of dose on the disease burden and infection dynamics. A: Comparison of the disease burden obtained with the nested model and the SIR model (*R*_0_ = 2.3). Each red dot represents the mean for 3800 to 4700 runs of simulation of the nested model, with *β* = 2.5 × 10^−5^ days^−1^. For the SIR model, the contributions of the sensitive and resistant strains to the disease burden are also shown. B: Burden curves (in patient days) for various values of *R*_0_. C-F: Exemplary population dynamics of the SIR model for four doses and *R*_0_ = 2.3. Note that the burden *B* is the area-under-the-curve of the sum of the blue and red curves (number of individuals infected by each strain at time *t*).

As can be intuitively expected, the overall trend is that the burden *B* decreases for increasing doses (Fig 5). *B* is maximal in the absence of treatment and minimal with very aggressive treatment (where the resistant strain cannot withstand the drug pressure either). Interestingly, however, for intermediate doses, the disease burden displays a local minimum (and maximum) unless *R*_0_ is large (Fig 5A and 5B and Fig XXV). One can already see that, when high drug concentrations are forbidden because they are toxic, the best dose choice to minimize *B* can be the one corresponding to this local minimum. For the following discussion of this result, remember that we only consider outbreaks where the disease burden is at least 300 patient days, meaning that an epidemic outbreak actually occurred. For the majority of cases, this implies quite a large burden of the order of 10^4^ patient days (see Fig I). Appearance of resistance is hence not limiting, and the population-wide dynamics of the resistant strain are mainly determined by its ability to spread. The large number of pathogens of both strains also allows us to draw on the deterministic SIR model, which describes the disease burden surprisingly well (Fig 5A and Fig V; however, note that the SIR overestimates the depth of the “valley” around the local minimum).

Fig 5A separates the contributions of the sensitive and the resistant strain to the disease burden, and Fig 5C–5F illustrate the disease dynamics of the SIR model for four different drug concentrations. For very low doses, the sensitive strain is barely affected by the drug (Fig 5C). It spreads and as a consequence, the number of susceptible hosts quickly drops below the threshold which would allow the rare resistant strain to spread. The burden caused by the resistant strain is therefore negligible (Fig 5A and 5C). For slightly larger doses (Fig 5D), as the dose increases, the sensitive strain gets more and more hampered by treatment. However, it is still able to infect a large number of individuals that become immune, and because of the lack of available susceptible individuals, the resistant strain cannot proliferate abundantly in the population. In this region, the disease burden has a minimum (see SI section S8.2 and Fig XV for an analysis with a simplified model with two strictly sequential epidemics). As the drug concentration increases further (Fig 5E), the resistant strain experiences stronger competitive release from the sensitive strain. In this regime, it not only outcompetes the sensitive strain, it also benefits from a large pool of susceptible individuals, left untouched by the sensitive strain due to its low fitness. It hence spreads quickly and causes most of the disease burden in an epidemic nearly as large as in the absence of treatment. For high doses (Fig 5F), where both strains have again similar fitness (i.e. recovery rates), the general dynamics are the same as at very low doses, but neither strain can spread well. Even though the reduction in the burden at the local minimum is not very pronounced, the difference in terms of days spent infected by the individuals of the population can be major in comparison to the neighboring local maxima (in the order of 10^4^ patient days of infection in a population of 10, 000).

For large *R*_0_ (Fig 5B), the disease burden as a function of dose is monotonically decreasing. For all doses, the sensitive strain is able to infect too many hosts to allow for a pronounced competitive release effect. Whether the disease burden is monotonically decreasing or displays a minimum at an intermediate dose depends on a complex interplay between the recovery rates and the transmission parameter. We give analytical details on this interplay in section S8.3, generalising the insights provided from the specific figures in the main text (see also Figs XVI, XVII and XIV). We moreover provide simulations for additional parameter sets in Fig XXV.

As a general trend, the burden increases with increasing *R*_0_. A larger transmission coefficient causes, in general, a larger outbreak. However, for intermediate drug concentrations, the disease burden is maximal for intermediate *R*_0_. This means that for such doses, a higher *R*_0_ does not lead to more days of infection as a whole, but to less of them. This occurs for doses around *c* = 0.5 where the competitive release effect is strongest. For large *R*_0_, as discussed above, there is no substantial competitive release, and most individuals get infected by the sensitive strain instead of the resistance strain, contrarily to what happens for these doses at lower *R*_0_. In the intermediate dosing regime, the recovery rate of the sensitive strain is substantially higher than that of the resistant strain. Thus, the disease burden caused by a sensitive-strain epidemic is much lower than the burden caused by a resistant-strain epidemic if intermediate doses are used. This leads to the observed maximum in the burden for intermediate values of *R*_0_ in this dosing regime.

Identical qualitative results are obtained if, instead of considering the disease burden as the number of days members of the population spend being infectious, we restrict the definition of the disease burden to the amount of days individuals spend showing symptoms. In fact, if we focus on symptomatic cases, the “valley” in the disease burden as a function of dose is even deeper than for the infectious burden (see Fig XII). For instance for *R*_0_ = 2.3 (*β* = 2.5 × 10^−5^ days^−1^), the reduction is 23% for the symptomatic burden, while it is only 11% for the infectious burden. The location of the minimum in the burden in dependence of the drug dose remains the same.

Note that we do not observe a non-monotonic behavior in the unconditioned disease burden *B*_0_(*c*), which considers epidemics of all sizes—including epidemics that die out very early. This is due to the rapid decline in the outbreak probability with increasing dose. (However, we can not entirely exclude that *B*_0_ is non-monotonic as well in certain parameter regions).

In Fig XIII, instead of the disease burden, we consider the total number of individuals infected during the course of the epidemic as a function of the drug dose. This measure also shows a local minimum. For high *R*_0_, this local minimum fades away, similarly as for the disease burden. However, contrarily to the disease burden, the total number of infecteds during the course of an epidemic monotonically increases with *R*_0_ for all doses. This confirms that the larger burden for intermediate *R*_0_ and doses around *c* = 0.5 is attributable to the differential recovery rates of patients infected with the sensitive or the resistant strain. In SI section S8.2, we simplify the true dynamics to two strictly sequential epidemics of the sensitive and the resistant strain and determine the total number of infected individuals of both epidemics. The simplified model reproduces the patterns observed in the full model.

### Trade-offs between treatment goals

From a comparison of Fig 3A and 3B, we have already seen that a certain dose choice might minimize the risk of *de novo* resistance at the within-host level but maximize the transmission of resistance at the population level and vice versa.

**Fig 6:**
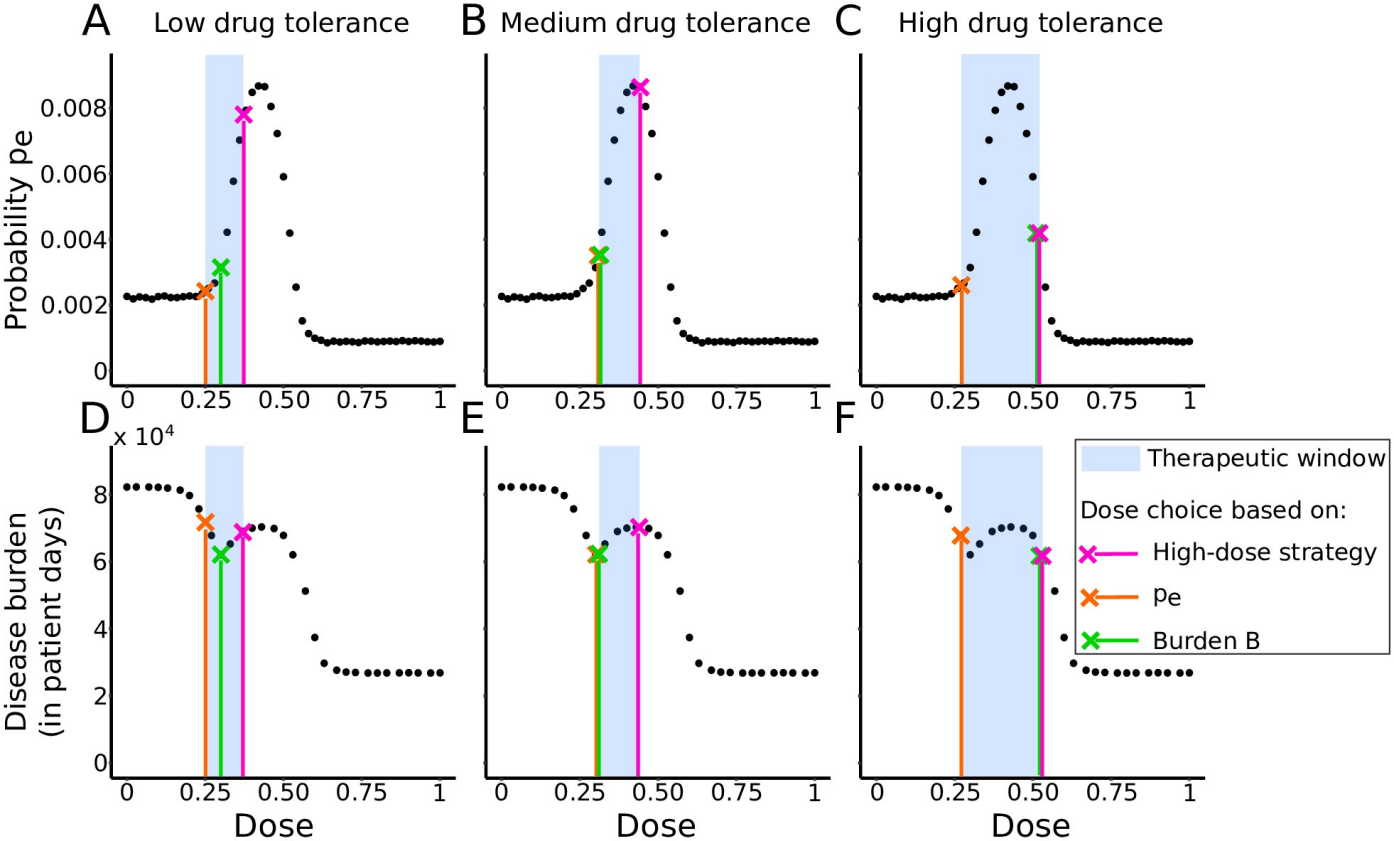
Comparison of three dosing strategies. Strategy that (1) uses the highest possible dose (pink), (2) uses the dose that minimizes the within-host probability of resistance (orange) and (3) uses the dose that minimizes the disease burden (green). *Top row*: Effect of each strategy on the within-host probability of resistance *p*_*e*_ for three therapeutic windows. The black dots represent probabilities of resistance emergence computed from simulations of the within-host model with varying drug doses. *Bottom row*: Effect of each strategy on the disease burden *B*, where we fix *β* = 2.5 × 10^−5^ days^−1^ (*R*_0_ = 2.3). The black dots represent disease burdens computed from simulations of the full nested model. The three therapeutic windows are: (A, D) low-drug tolerance window (0.25 < *c* < 0.37); (B, E) medium drug-tolerance window (0.32 < *c* < 0.45); (C, F) high drug-tolerance window (0.27 < *c* < 0.52).

Here, we compare the within-host probability of resistance and the disease burden for three different strategies: (1) using the highest possible dose, (2) using the dose that leads to the lowest within-host probability of emergence *p*_*e*_ (cf. Day and Read, 2016 [12]), and (3) using the dose that leads to the lowest disease burden *B*. This comparison, shown in Fig 6, is done for three different therapeutic windows. As can be seen, a dosing strategy that is optimal to achieve one goal may be suboptimal under another measure of treatment success. E.g., for the first window (Fig 6A), representing a low drug-tolerance scenario, the dose leading to the lowest *p*_*e*_ (orange cross on the figure) leads to the highest disease burden at the population level. Choosing the highest possible dose (pink cross) leads to a suboptimal disease burden as well. For the therapeutic windows in Fig 6B and 6C, each time, two of the three strategies yield the same outcome, while the third one differs. Other therapeutic windows can lead to even different conclusions, with, for instance, all of these three strategies agreeing on choosing the highest available dose.

### Model extension: Incomplete treatment coverage

The model assumes a perfectly homogeneous population. We now introduce a form of population structure by extending the SIR model to include incomplete treatment coverage. Infected patients receive treatment with probability *f* and remain untreated with probability 1 − *f* (see SI section S4 for the model equations).

**Fig 7:**
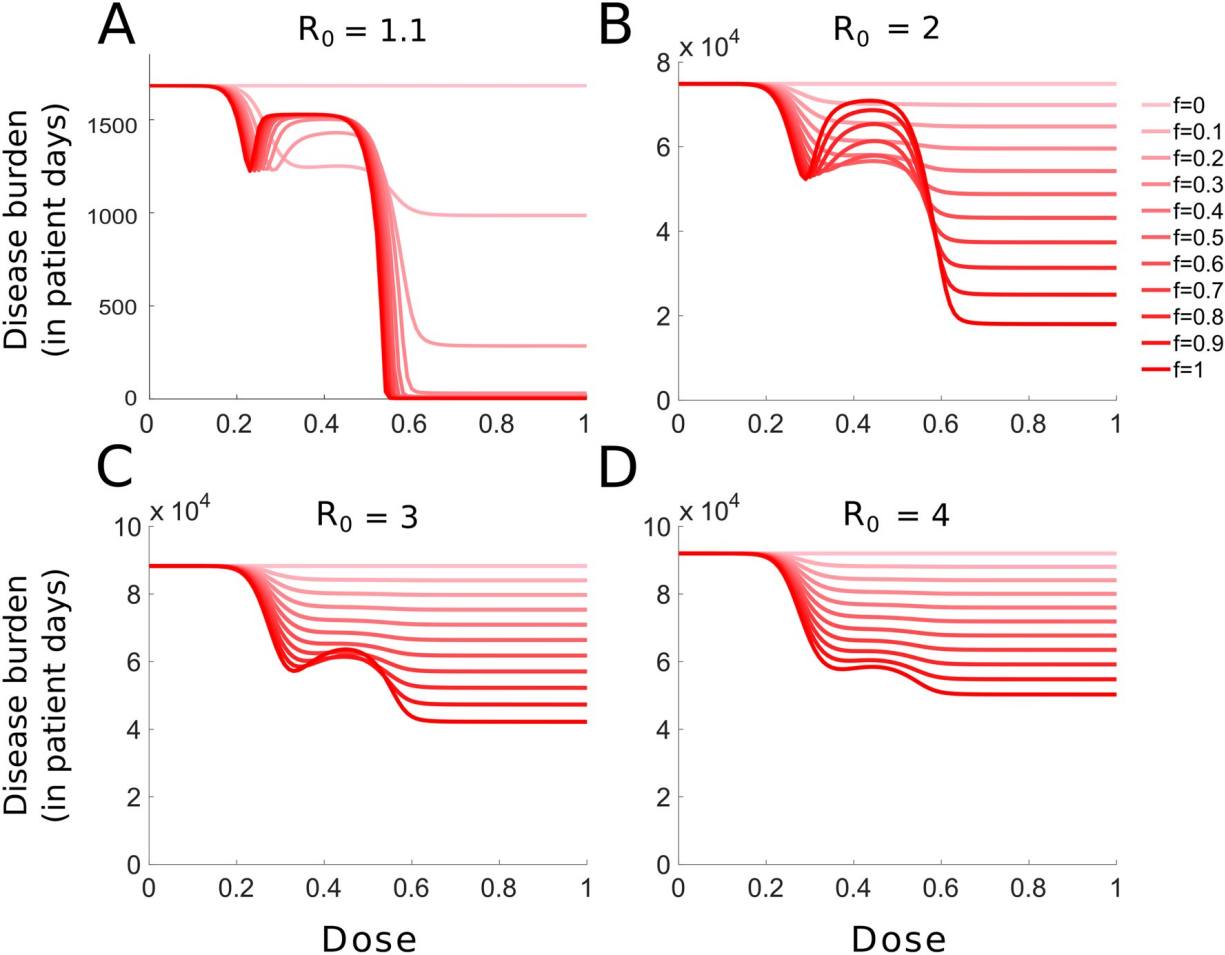
Effect of the treatment coverage on the disease burden. The disease burden for various fractions *f* of treated hosts is shown, ranging from no treatment (*f* = 0, pale red) to full treatment (*f* = 1, dark red). The basic reproductive number is given by: (A) *R*_0_ = 1.1; (B) *R*_0_ = 2; (C) *R*_0_ = 3; (D) *R*_0_ = 4. Note that the scale on the y-axis in panel A is very different from the other panels.

For all four values of *R*_0_ considered in Fig 7, the burden as a function of dose becomes monotonically decreasing as coverage gets lower. Therefore, for low treatment coverage, the optimal dose-choice to minimize the burden is the highest possible dose, regardless of where the therapeutic window lies. However, as can be seen in Fig 7A–7C for intermediate doses, the disease burden displays an intermediate minimum as a function of treatment coverage. For intermediate doses (roughly 0.3 < *c* < 0.55), treating only a fraction of the population (incomplete treatment coverage) is more beneficial to the population as a whole than treating all infected hosts. In the case of a complete coverage, the resistant strain dominates the pathogen-mix in the population and produces full-fledged epidemics. Conversely, for very low coverage, the sensitive strain dominates. For intermediate coverage, the two strains compete for hosts, resulting in a suboptimal epidemic in total.

In SI section S5.3 and Fig IX, we consider the emergence of resistance in an endemic disease (modeled by an SIR model with birth and death) and find similar non-trivial dependencies on treatment coverage.

## Discussion

From a within-host model, previous work concluded that, in order to minimize the risk of resistance, either the highest tolerable dose or the lowest effective dose should be used [12]. Between these two doses, the right choice depends on where the therapeutic window lies with respect to the “inverted-U-shaped” curve that describes the relationship between the administered dose and the probability of emergence of resistance.

When it comes to infectious diseases, emergence and spread of antimicrobial resistance are not only determined by possibilities for resistance to emerge inside one infected host, but are also strongly influenced by the population-level dynamics of the disease [30, 31, 36]. Therefore, we developed a nested model of within-host and between-host dynamics –using simple general assumptions about the pathogen dynamics and the transmission process– to study the effect of drug dose on several measures of treatment success at the population level (outbreak probability, transmission of the resistant strain in the population, disease burden). We showed that these different population-level criteria may lead to different dosing recommendations. What is more, they can strongly differ from recommendations based on measures at the within-host level.

By using general models at both scales, we obtained insights into the fundamental mechanisms generating trade-offs between treatment goals at the two levels. While we used a single set of within-host parameters throughout the main text of the manuscript, we took several steps to back up our results. Specifically, in addition to the nested model, we analysed the stochastic and deterministic dynamics of an SIR model. This enabled us to uncover patterns at the between-host level and to determine the influence of the recovery rates and the within-host probability of resistance on the observed patterns. To make the link to the full model, we performed a parameter sensitivity analysis to see the influence of the within-host parameters on the recovery rates and the within-host probability of resistance. In combination, the analytical results from the SIR model and the parameter sensitivity analysis of the within-host dynamics allow to understand the behavior of the full model. For further confirmation, we performed simulations of the full (computationally expensive) model for a limited set of within-host and between-host parameters.

### Which dose do the four criteria suggest?

The dose choice required to minimize the outbreak probability is the highest non-toxic dose. Therefore, in an ideal case with a perfectly-monitored population, using aggressive antimicrobial chemotherapy on the first few infected patients is best to minimize the probability of an epidemic breaking out.

The optimal strategy to adopt is not as straightforward if the aim is on suppressing antimicrobial resistance. Since the probability of emergence of resistance within one host shows a peak when varying the treatment intensity, one must take into account the therapeutic window and choose one of the boundaries to minimize it [12]. But this probability is not enough to assess the consequences of a dose choice at the population level in the case of infectious diseases. The transmission characteristics of the pathogen of interest need to be taken into consideration.

Criteria of treatment success that focus on the emergence and spread of resistance in the population are fundamental for assessing the influence of different treatment strategies on drug resistance. We chose the number of transmission events of the resistant strain as such a success criterion. As the within-host probability of resistance, this measure displays an intermediate maximum. Yet, the peak in the number of resistant strain transmission events is not necessarily at the same dose as the within-host probability of resistance. For pathogens with high transmission coefficients, the peaks are located at similar doses, and the individual- and population-level measures of the risk of resistance lead to similar conclusions. In contrast, for pathogens with rather low transmission coefficients, the peak in the number of resistant transmission events is shifted towards lower doses. This effect is caused by the rapid decrease in hosts infected by the wild-type strain when the treatment strength is increased. In that case, adopting a dose that minimizes the probability of emergence of resistance inside a host may actually maximize the average transmission of resistance in the population. Thus, the strategies built for minimising pathogen resistance from an individual or a population standpoint can be perfectly antagonistic. These results remain true when relaxing the closed-population assumption of our model by adopting an SIR model with birth and death, in which the population is at the endemic equilibrium prior to the emergence of resistance (see section S5).

As for minimizing the overall disease burden borne by the infected population the best strategy strongly depends on the pathogen’s transmission coefficient. We find that in parameter regimes with low transmission coefficients, a local minimum, maximum, or both, can be observed within the therapeutic window of interest. In that case, the optimal dose to minimize the disease burden can range from the lower bound of the therapeutic window to its higher bound. In particular, intermediary doses inside the therapeutic window can be the most beneficial doses. For higher transmission coefficients, the disease burden decreases monotonically with the treatment strength. Then, aggressive chemotherapy is consistently optimal, as it is optimal for minimizing the outbreak probability.

We also show that incomplete treatment coverage (e.g., due to asymptomatic cases or infecteds not seeking medical help) can alter the optimal dose choice. In particular, the disease burden decreases monotonically for low treatment coverages. For epidemics in which only a small portion of the population can be treated, adopting aggressive chemotherapy can be beneficial to the population as a whole, regardless of the *R*_0_. Moreover, we show that for intermediate doses (which favor the resistant strain), the disease burden displays a local minimum as a function of coverage for low transmission epidemics. There, a partial coverage stimulates competition for hosts between the resistant and sensitive strain, resulting in a smaller epidemic overall (see also [37–40] and discussion further below). It can therefore be beneficial to the population as a whole to actively reduce coverage in cases where the therapeutic window is restricted to these intermediate doses.

Overall, the four criteria do not always agree on what the best dose choice is, and trade-offs exist between different treatment goals, which raises the question of which criterion should be used.

### Which criterion should be used?

Obviously, it is most desirable to prevent a large outbreak from occurring in the first place, which is best achieved with high drug doses. However, this requires to detect and to treat all (or at least most) of the early cases, which might not be feasible in reality. Moreover, even with the highest drug dose and perfect treatment of all symptomatic cases, the outbreak probability is still about 40% for the parameter choice of Fig 4 and an intermediate *R*_0_ of 2.3. Our intuition (which is supported by the considerations in SI section S2 and Fig II) is that the possibility of resistance generally does not affect the outbreak probability. Resistance normally only has a small probability to appear in any one host and hence most likely emerges in the population only once a major outbreak has started. However, since people travel (which we do not consider in our model), resistance might be imported from the outside [41]. In this case, using high drug doses is likely to be less efficient in reducing the risk of a large outbreak (see also a corresponding comment in [37]).

When the epidemic cannot be contained and a large outbreak occurs, the criterion that should guide decisions is not obvious and largely dependent on the disease of interest. Preventing the evolution and spread of resistance is not a goal per se (otherwise, not treating at all would be best). Tanaka et al. (2014) distinguish direct and indirect benefits of treatment [42]. Direct effects of treatment refer to the successful therapy of infected individuals, reducing their morbidity (or mortality). Indirect effects focus on the population as a whole and have been quantified based on the total number of cases over the course of the epidemic [37–40, 42, 43]. The disease burden as a measure combines both of these effects since it is reduced both by the prevention of cases and the successful treatment of infections. The disease burden (restricted to symptomatic cases) can be understood as the total number of days infected individuals of a population spend being incapacitated by the infection. It can therefore be most relevant for diseases that would cause symptoms severe enough to force infected individuals to stop their activities for a few days, but not severe enough to cause any deaths.

The disease burden is a meaningful criterion from an utilitarian point of view. However, it ignores the perspective of any specific patient once treatment is administered. The wish of any patient is to recover as quickly as possible, and physicians usually take their decisions accordingly. We have not included this as a criterion in the main part of the manuscript. However, from a look at the mean recovery rates (Fig 2B) and the low probability of within-host resistance (Fig 2C), one can immediately see that to achieve this goal, the patient should be given the highest possible non-toxic dose. Since under some circumstances, the disease burden suggests a low-intermediate dose (and intermediate treatment coverage), there can be a conflict between the interest of the individual patient and of the population as a whole.

In the present article, the disease is non-lethal for all patients, even in the absence of treatment. For diseases with a potentially fatal outcome, the reduction in mortality would be a highly relevant population-level criterion. At the level of the individual, the within-host probability of resistance would be important since the evolution of resistance would not simply delay recovery but substantially increase the risk of death. A risk of pathogen-induced mortality could be included into our model, e.g. by introducing a risk of lethality beyond a certain pathogen load. The dose-choice strategies would then have to account for the necessity of containing the pathogen load below potentially-lethal levels [44].

We assume that all patients are immuno-competent. However, some hosts can be immuno-compromised and therefore be particularly vulnerable to treatment failure. Even if the disease is non-lethal to the majority of the population, it could pose a major risk to these patients who are hence extremely dependent on the availability of efficient drugs. To protect them, suppressing the spread of resistance in the population would be a priority, and population-level measures of resistance emergence and spread would be very relevant. The drug dose administered to the immuno-competent part of the population should then maximally limit the spread of resistance even at the cost of delayed recovery. Obviously, higher doses would be required to treat the immuno-compromised patients.

Optimally, of course, we would not need to make compromises, prioritizing one treatment goal over another. Our work shows that with modulating nothing but the drug dose, this is not possible. In order to reconcile the different scales and criteria, additional measures are necessary. For example, using drug combinations might be a way to achieve rapid patient recovery while keeping the risk of resistance low. Trade-offs between the individual and the population levels may be alleviated through the isolation of symptomatic patients.

### Why and when do differences between individual- and population-driven strategies arise?

Trade-offs between treatment goals appear for two reasons. First, some criteria (outbreak probability, disease burden) count infections both with the sensitive and with the resistant strain, hence explicitely taking treatment effects on both into account, while other criteria (resistance emergence at either level) only focus on the resistant strain. Second, and more interestingly, several forces act and drive the evolutionary dynamics at both levels, leading to differences between criteria at the within-host and between-host scales.

**Fig 8:**
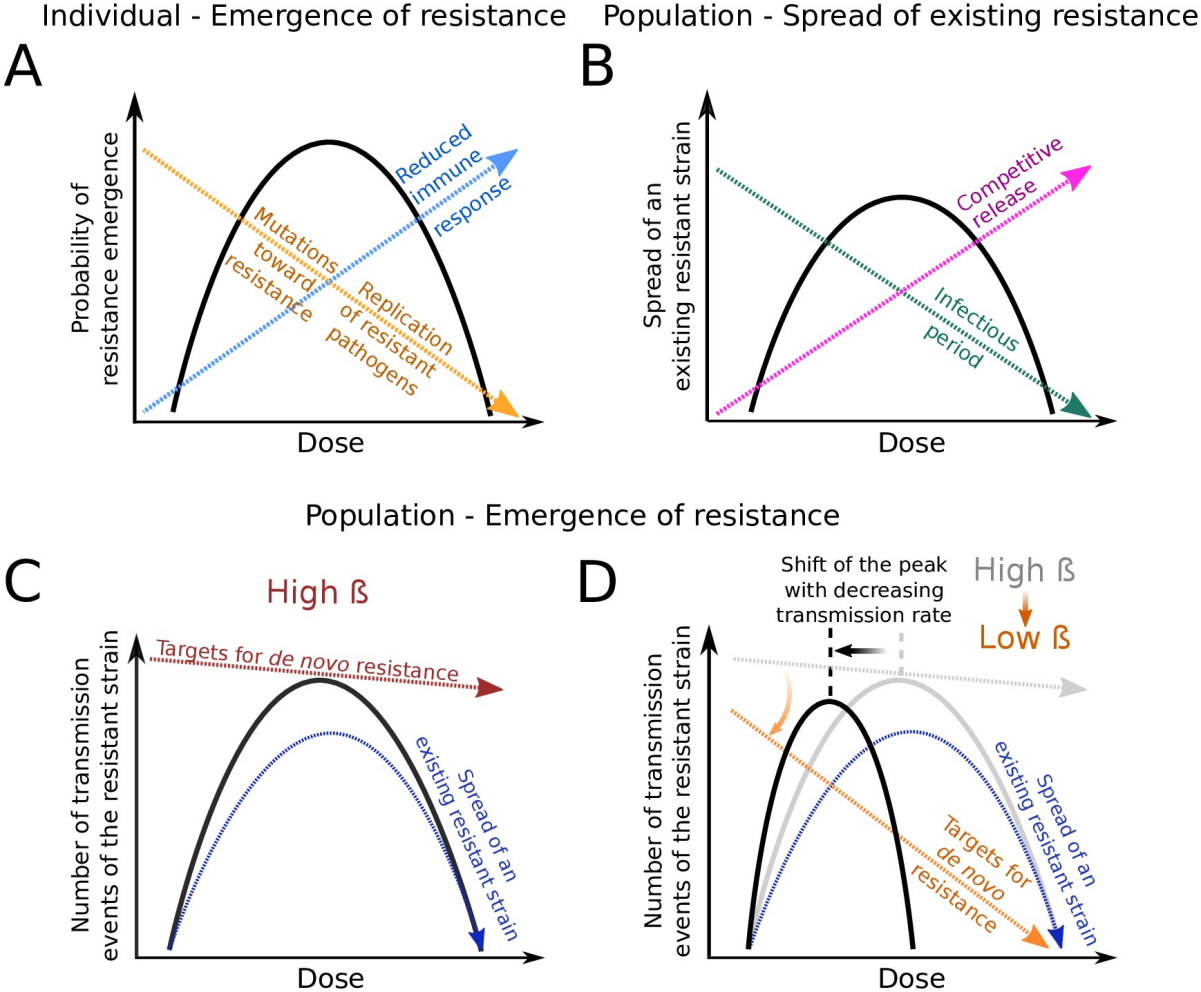
Risk of resistance and the factors shaping it at the individual and population levels. (**A**) The within-host probability of resistance emergence (solid black line) is shaped by three forces that are in part inversely affected by the drug dose: the strength of the immune response triggered by the sensitive strain (dashed blue arrow), the mutations of sensitive-to-treatment pathogens towards resistance during treatment (dashed orange arrow) and the rate of clearance of the resistant strain through the drug (dashed orange arrow as well). This panel is based on Figure 1 from Kouyos et al. [5] and adapted to represent the scenario we model. (**B**) The spread of an existing resistant strain in a susceptible population (solid black line) also has an inverted U-shape. Increased drug pressure favors the spread of a resistant strain through competitive release (dashed pink arrow). However, with increasing dose, the infectious period (dashed green arrow) decreases, even for infections with the resistant strain. (**C,D**) Number of transmission events of the resistant strain, at a high (C) or low (D) transmission coefficient *β*. This measure (solid black lines) depends on the ability of a resistant strain to spread (dashed dark-blue arrows, see panel B), and on the rate of *de novo* development of a resistant infection. This latter rate depends on the number of sensitively infected patients (or “targets for *de novo* resistance”), which in turn depends not only on the drug dose but also on *R*_0_. With a large *R*_0_, large outbreaks of the sensitive strain guarantee a high probability of appearance of resistance (red dashed line in Fig 8C). In contrast, for low *R*_0_, this probability severely decreases with increasing drug dose (dashed orange arrow in Fig 8D). The location of the peak in the number of transmission events of the resistant strain therefore depends on the transmission coefficient of the disease. The black curve is essentially the product of the red (or orange) arrow with the dark-blue arrow. The grey line and arrow in panel D are a repetition of the red arrow and black line in panel C.

Fig 8 conceptually summarises the various dose-dependent forces and factors that affect the dynamics of the resistant strain at the individual and population levels. At both levels, the dynamics are determined by the *de novo* appearance of resistance and by the spread of existing resistance. Let us consider spread of resistance first. At the within-host level, the factors that shape the spread of resistance (once there) are (1) the direct effect of the drug on the resistant strain and (2) the suppression of the resistant strain through the host’s immune response (Fig 8A). Spread is easiest at doses for which the difference in the within-host growth rates of the strains is largest. At the population level, the factors that determine how well the resistant strain spreads once it has emerged are the infectious period for infections with this strain (directly affecting the number of secondary cases) and the strength of competition for susceptible hosts with the sensitive strain (Fig 8B). Spread is easiest at doses for which the difference in the recovery rates for infections with the sensitive and the resistant strain is largest. Since the recovery rates mirror the within-host growth rates, it appears plausible that spread of the resistant strain in the population is maximal for similar doses as the within-host spread of the resistant pathogens.

Yet, it is not sufficient to consider spread of existing resistance. The appearance of resistance needs to be taken into account as well. This can shift the peaks in the risk of resistance at either level to lower doses. If one is shifted more than the other, a trade-off between these two criteria appears. From our analysis, we can draw some inferences of when this likely occurs.

Within hosts, the number of *de novo* mutations during treatment decreases with the drug dose. For our model and the parameter regimes considered, this has little effect on the location of the peak (unless the immune system is very bad), and the within-host probability of resistance is maximal where spread of resistance is easiest. (As a caveat, we point out that we only changed one parameter at a time in the parameter sensitivity analysis; small changes in several parameters may have an effect even if changes in single parameters do not, as found in [30]).

At the population level, we need to consider the rate at which individuals develop resistant infections. This rate does not only depend on the within-host probability of resistance but also on the number of sensitively infected hosts who might develop a resistant infection. This number in turn depends on the drug dose. If it becomes limiting for high drug doses, the peak in the between-host transmission of resistance is shifted to lower doses. We find that a low *R*_0_ as well as a strong effect of treatment on recovery favour such a shift (comparison of Fig 8C and 8D). In these cases, the curves describing the risk of resistance at the individual and population levels may peak at very different drug doses, and considerations at the population level may suggest higher doses than considerations at the individual level. Note that this trade-off goes into the opposite direction than the one discussed in [30]. Colijn and Cohen (2015) discuss the possibility that high-dose treatment is best at the individual but worst at the population level [30]. We find this situation only with bad immune systems (but this does not exclude that it appears more readily with different modeling assumptions or in parameter regimes that we did not consider). However, in this context, it should be noted that the within-host probability of resistance affects the between-host resistance (meaning that a shift of the former to lower doses may also shift the latter to lower doses) but not the other way round.

Trade-offs between minimising the within-host probability of resistance and minimising the disease burden essentially arise because the disease burden caused by the sensitive strain increases with decreasing dose. This may lead to situations where the within-host probability of resistance can be lowered by lowering the dose but at the expense of increasing the disease burden due to the higher number of sensitive infections.

The outbreak probability always suggests to use the highest possible dose and is hence in conflict with the other measures of treatment success whenever these suggest to use lower doses. The reason is that the outbreak probability is the only measure that is not affected by the dynamics of the resistant strain (unless the within-host probability of resistance is extremely high).

### The concept of the therapeutic window

Whenever the relationship between the criterion used for the dose assessment and the drug dose itself is non-monotonic, whether the criterion shows a single peak (e.g. *p*_*e*_) or several local extrema (disease burden), any recommendation on the best dose is strongly dependent on where the therapeutic window lies. The therapeutic window depends on the minimum effect of treatment and the maximum of side effects that is considered acceptable. In our model, the recovery rate changes substantially across the therapeutic window. Besides clearing the pathogen, treatment could in addition directly mitigate symptoms. This would be another component of treatment effect. If low doses are similarly good at mitigating symptoms as high doses, the effects of treatment could be considered more similar at low and high doses despite differential recovery rates (however, in our model, symptoms are directly linked to the pathogen load).

It is quite likely that the therapeutic window varies between patients. Adjusting prescribed doses to the body weight of each patient is a way to partially account for host heterogeneity. Still, acquiring accurate knowledge on the therapeutic window for each patient may be challenging. Prescribing low-dose treatments can be dangerous if the lower bound of the therapeutic window is underestimated for a given patient. Here, the disease modeled is self-limiting and all hosts are immuno-competent such that under-dosing would not be life-threatening, but it will be for many diseases. The same can be true for overdosing.

What is relevant for our study (as it is for others) is the relationship between drug dose and the in-vivo probability of resistance evolution within the therapeutic window. It is hard to obtain this information for a given disease and drug. Yet, clarifying this relationship can help to guide the development of new treatment strategies.

### Optimal treatment strength at the population level: Similarities between drug dosing, coverage, and population-wide timing of treatment

The strength of treatment can be modulated in various ways. One of them is the choice of the drug dose as considered in the present study. At the population level, it is also possible to vary the fraction of the population that receives treatment (“coverage” or “treatment level”) and the optimal time point during the course of the epidemic when antimicrobials start being used in the population. The optimal choice of these two factors, given the risk of resistance evolution, has been assessed in a series of studies on optimal drug use during influenza epidemics (e.g. [37–39, 42, 43, 45]).

There are many similarities between the findings of these studies and the present article. First, high treatment coverage right at the start of the epidemic might contain the spread of the disease, and so does high dose treatment in our model. More subtle is the case when containment is impossible or has failed. If the number of susceptible hosts is a limiting factor to the disease spread—as it is in our simulated study and as it can be in real-life epidemics (e.g. influenza) –, the disease dynamics in the population is strongly affected by competition between the sensitive and the resistant strains for susceptible hosts. By choosing the right treatment strength (which may refer to drug dose, coverage, or timing of treatment), this competition may be exploited to reduce the overall negative impact of the disease on the population. Essentially, treatment needs to be strong enough to impede the wide spread of the sensitive strain but weak enough to allow enough individuals to receive immunity through infection with the sensitive strain; then, spread of the resistant strain is hampered due to the reduced pool of susceptibles. If the strength of treatment is too strong, so-called “overshooting” occurs. Too many susceptibles remain untouched by the sensitive strain to establish herd immunity that would prevent or at least mitigate a time-delayed resistant epidemic [46]. The resistant strain then profits from competitive release.

With treatment strength referring to drug dose, this competitive release is translated in an optimal spread of resistance at high-intermediate doses that efficiently suppress the sensitive but not the resistant strain (cf. the prediction in [5]). The disease burden and the total number of infecteds therefore display a maximum in this dose range. In some low-intermediate dose range, where the sensitive strain is suppressed well but not well enough to allow for competitive release, we find a local minimum. Likewise, intermediate coverage or a delay in the use of antimicrobials in the population may be best in reducing the total number of cases [37–39, 42, 43]; see also our Fig 7. However, if the resistant strain suffers a strong transmission cost (i.e. *β* is much lower for the resistant strain), high coverage yields the best outcome [37, 39]. We have not implemented such a transmission cost into our model, which might alter results. Another factor that has been considered in the context of influenza epidemics is prophylactic treatment, which—once the resistant strain has appeared in the population—substantially facilitates its spread since it meets little or no competition for the treated hosts with the sensitive strain [37, 45].

### Limitations and extensions

The interactions between pathogen load, treatment, immune response, and symptoms are complex and depend on the disease and the drug. In our model, symptoms set in once the pathogen load reaches a given threshold. This implies that they are directly caused by the pathogen. However, symptoms can also be due to the action of the immune system, e.g. fever can be part of the immune response. Within our setup, this would mean that the onset of therapy would depend on the level of the immune response, *I*, rather than on the pathogen load, *P*_*w*_ + *P*_*m*_. In that case, the drug dose may have less effect on the strength of the immune response. On the other hand, drugs can also have a direct effect on the immune system, which would be dose-dependent. For example, they can mitigate corresponding reactions such as fever. Both factors would change the relationship between dose and within-host probability of resistance evolution and hence conclusions on optimal dosing (where the notion of resistance assumes that treatment does not only affect the immune response but also the pathogen itself). On the other hand, resource competition also leads to an inverted U-shaped relationship between the within-host probability of resistance and the drug dose, even for different interactions between treatment and the immune system [12, 47].

In our study, we assume that upon recovery, each patient has built up an immunological memory that protects the individual from reinfection at least until the end of the epidemic. This happens independently of the level of the immune response, *I*, reached during the infection. However, with a high drug dose that quickly suppresses the pathogen, no strong immune response might be triggered and no (or only weak) immunological memory might develop. In that case, reinfection cannot be excluded, and the disease burden could be higher for aggressive therapy than predicted by our model. To investigate this, a more accurate modeling of the immune system would be necessary, following e.g. Ankomah and Levin (2014) or Gjini and Brito (2016) [28, 29].

Our within-host model also ignores classical ecological factors such as spatial structure and temporal changes in drug pressure. Drug penetration is not equal across body tissues, potentially creating compartments with high and low drug concentrations. This spatial drug heterogeneity has major implications for the evolution of drug resistance [48–50], and it will also affect the dose-dependent recovery rate. Likewise, changes in the drug concentration upon drug intake and subsequent decay (pharmacokinetics) might have an effect on the within-host dynamics of the two strains. In this context, it would also be interesting to include incomplete adherence to treatment, where patients skip doses or stop treatment prematurely. Omitted doses could lead to a lower “effective dose”, i.e. aggressive chemotherapy might, for example, resemble treatment with intermediate doses.

On the genetic side, considering only one resistant genotype is a major assumption. Normally, multiple different mutations confers resistance to antibiotics, where only a few lead to high-level resistance, while many lead to low-level resistance (and might potentially accumulate to achieve resistance to high doses). How this alters the prediction of an inverted U-curve for the probability of within-host resistance needs to be investigated within a more complex within-host model.

Another set of assumptions concerns the between-host level. The mode of pathogen transmission assumes transmission of one strain only during a transmission event, even when the infecting host carries both pathogen strains. This assumption is justified if the two strains are not well-mixed inside the disease-carrying host and if the mode of transmission is likely to produce an inoculum consisting of pathogens that were originally grouped together. Alternatively, in other cases, transmission of a mixed inoculum containing pathogens drawn randomly in proportion with the load of each strain in the infecting host may be more realistic. This would probably alter the speed of spread of resistance in the population and mitigate strain competition, presumably leading to different results regarding optimal drug-dose choices. Another important assumption with consequences for the spread of resistance is the assumption of full cross-immunity between strains. Also, as in the within-host model, consideration of multiple resistant strains and the step-wise acquisition of mutations over several patients could be an extension towards more realism.

Also, in our model, the transmission coefficient remains constant throughout the course of the infection. However, in many cases, patients will stay at home or be isolated, once symptoms set in. Another potential extension of our work would therefore be to include a diminished transmission coefficient for individuals with declared symptoms. Moreover, we did not consider an effect of the pathogen load, the intensity of treatment, or the immune system on the transmission coefficient. It was found in rodent malaria experiments that in mixed infections with a low initial frequency of resistance, aggressive treatment leads to more transmission than mild treatment [14]. Such effects are not captured by our modeling approach.

We considered a homogeneous and well-mixed population. Variation in the number of contacts (e.g. “superspreaders”) and more generally the contact structure of the social network influences the disease dynamics (see for instance [51, 52]). This structure moreover affects the invasion of a second—for us, the resistant—strain [53]. It would be interesting to investigate how different contact structures affect optimal drug dosing. It might also turn out to be optimal to use different doses for different people, depending on their position in the network and their number of contacts. Individuals moreover differ in their immune response, as mentioned above.

For the antibiotic treatment of bacterial infections, bystander selection of resistance in the commensal flora is an important aspect and may be dose dependent [54–56]. Assessing the relationship between dose and resistance evolution in the commensals is beyond the scope of our study but of high practical relevance.

### Conclusions

While real-life scenarios might differ from our model in many respects, our model and its analysis (involving simplified epidemiological models) provide general insight into how dose-related trade-offs between different treatment goals and scales can arise.

One of the primary drivers of the trade-offs we observe is the dose dependence of the infectious period. Epidemiological parameters that are dose dependent (such as the period of infectiousness) influence the disease dynamics at the population level, bringing the problem of optimal dosing from the individual to the population. Importantly, both the within-host and the between-host dynamics affect the evolutionary dynamics of the pathogen and hence selection for resistance. Choosing the optimal drug dose within the therapeutic window can prove non-trivial, and the right decision depends on the measure of treatment success.

Our results draw attention to an important problem, which is that finding the right dose is not just a within-host matter. We hope that this study will encourage future empirical work on the respective benefits of low vs high doses to include the dynamics of transmission of the pathogen of interest, instead of focusing on isolated hosts. Both within-host and between-host scales need to be taken into account for disease and resistance management.

## Supporting information

S1 Text Full supporting information (text and figures). (PDF)
