# Supplementary material for: Aggressive or moderate drug therapy for infectious diseases? Trade-offs between different treatment goals at the individual and population levels
Source: PLoS Comput Biol. 2019 Aug 12;15(8):e1007223. doi: 10.1371/journal.pcbi.1007223 (PMC6742410; doi:10.1371/journal.pcbi.1007223)
Supplement: S1 Text — (PDF) [file pcbi.1007223.s001.pdf]

Supporting Information

Aggressive or moderate drug therapy for infectious diseases? Trade-offs between different treatment goals at the individual and population levels

Jérémie Scire, Nathanaël Hozé, Hildegard Uecker

S1: Distribution of the disease burden

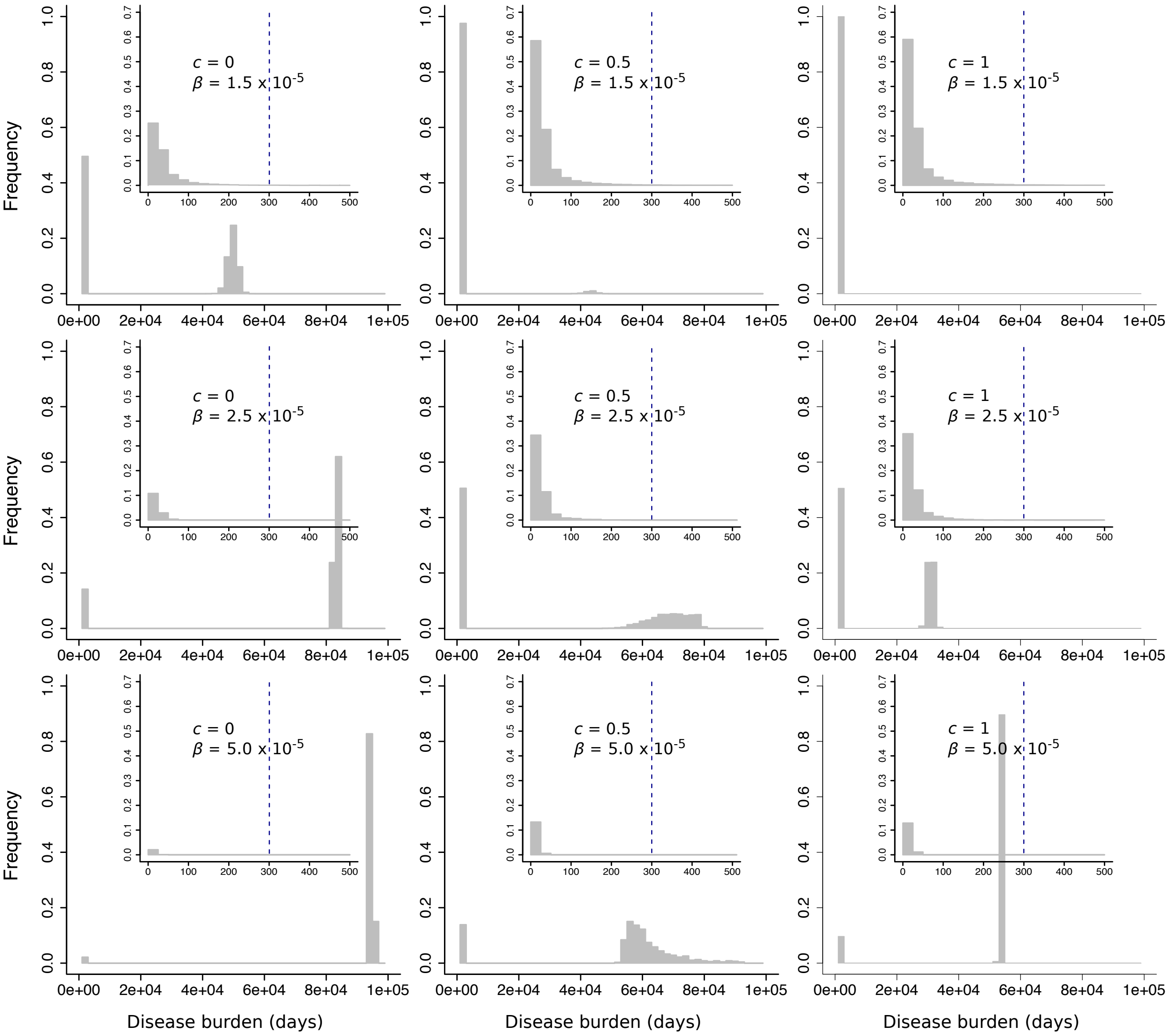

Figure I. Distribution of the disease burden B_0. Left column: absence of treatment ($c = 0$). **Middle column:** medium treatment strength ($c = 0.5$). **Right column:** maximal treatment strength ($c = 1$). **Top row:** Low infectivity ($\beta = 1.5 \times 10^{-5}$). **Middle row:** Medium infectivity ($\beta = 2.5 \times 10^{-5}$). **Bottom row:** High infectivity ($\beta = 4.5 \times 10^{-5}$). Each panel comprises of a histogram of the disease burden for ≥ 1800 simulation runs per parameter set (up to 20,000). The insets represent zoom-ins into the region of low disease burdens (between 0 and 500). The blue vertical dashed line in these insets represents an arbitrary threshold for defining a "successful outbreak", set at 300 days. For most parameter values, this threshold sets a clear limit between the two sub-distributions of B_0. The simulation runs are divided then into two types, one type produces small to nonexistent outbreaks whereas the other produces full-fledged outbreaks. The only parameter sets for which this distribution of disease burdens B_0 is unimodal and concentrated around 0 correspond to high doses combined with low transmission coefficients (top-right panel).

S2: Derivation of the outbreak probability from the distribution of secondary cases

We define the outbreak probability as the probability that the disease burden reaches at least 300 days. Unless R_0 is small and the dose high, the distribution of the burden is bimodal with either very large or very small epidemics. We therefore can determine the outbreak probability as the probability of non-extinction of a branching process. For this, we use the distribution of secondary cases obtained from simulations, $P_{\mathrm{ABM}}(Y = k)$. The corresponding probability generating function is given by

$$f(s) = \sum_{k=0}^{\infty} P_{\mathrm{ABM}}(Y = k) s^k, \tag{S2.1}$$

and the extinction probability q of the process is the smallest fixed point in $[0, 1]$:

$$q = f(q), \tag{S2.2}$$

see e.g. [1]. We can determine q numerically, and obtain the outbreak probability as $P_{\mathrm{outbreak}} = 1 - q$.

This approach is not suitable for small R_0 and high doses, where the distribution of the burden is unimodal but is in very good agreement with simulation results when the distribution is bimodal (see Fig II). Since the number of secondary infections is determined in the absence of resistance evolution, this confirms that the possibility of resistance evolution is negligible for the assessment of the outbreak probability (at least when the burden distribution is bimodal).

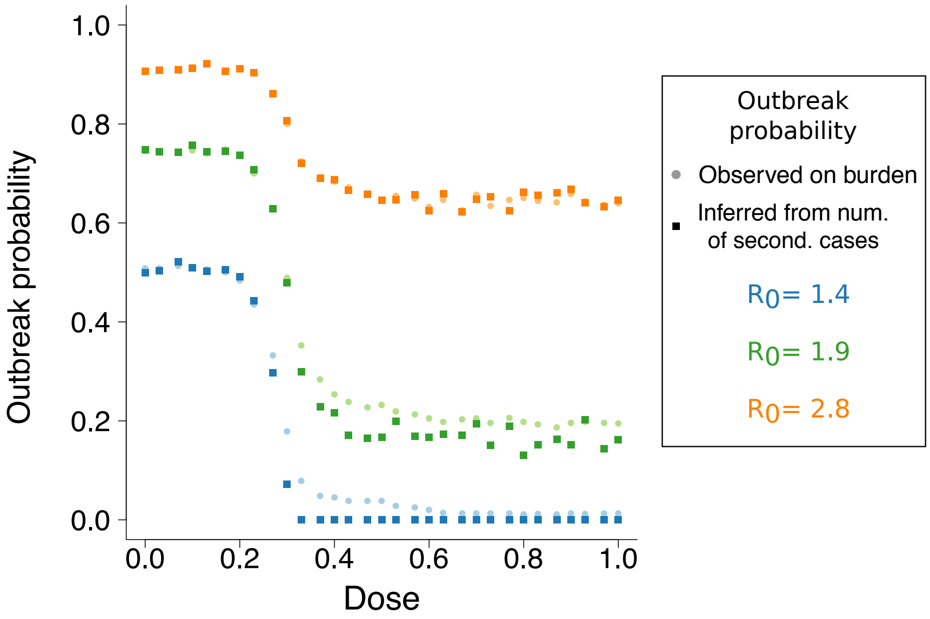

Figure II. Comparison of the outbreak probability as observed from the measured disease burden and the outbreak probability calculated from the distribution of the secondary cases of the first infected individual. Values obtained with three different transmission coefficients are presented. The squares represent the value calculated from the distribution of secondary cases. The dots, in a lighter shade, represent the value measured from the disease burden observed in the simulations. The truncated arrows represent the 95% confidence intervals of the mean. As expected, the calculated outbreak probability gives accurate results except for situations with low transmission coefficient and high dose, as seen in the blue curves and, to a lesser extent, in the green curves. In blue: $\beta = 1.5 \times 10^{-5}$. In green: $\beta = 2.0 \times 10^{-5}$. In orange: $\beta = 3.0 \times 10^{-5}$. Between 2200 and 22000 runs of simulation were performed per transmission coefficient and treatment dose.

S3: Relation between the nested agent-based model and the classical SIR model

S3.1 Transition from the agent-based model to the SIR model

In this section, we describe in more detail the transition from the full nested model to the classical SIR model defined by Eq (3) in the main text. The flow diagram of the SIR model is shown in Fig III.

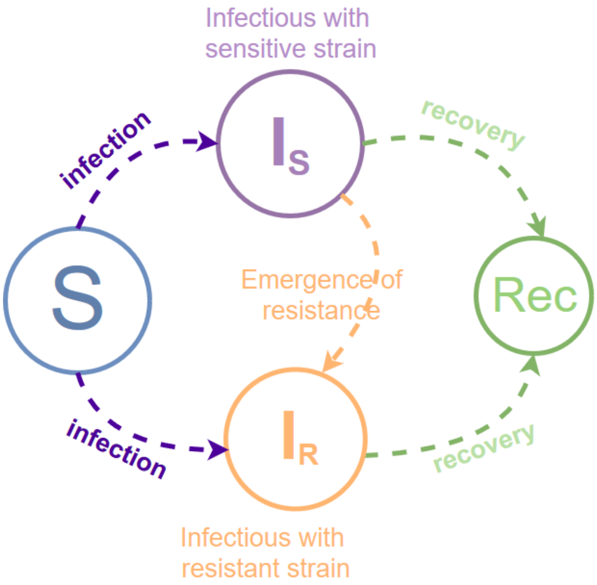

Figure III. Diagram of the SIR model.

The parameter values for the SIR model directly derive from simulations of the within-host model Eq (1) (see Fig 2B-C). The recovery rate γ_S (γ_R) is measured as the average time a single individual infected with the sensitive (resistant) strain spends being infectious. It is estimated over 10^5 stochastic runs of the within-host model. The probability of emergence p_e is measured as the fraction of runs where the resistant pathogen load reaches a threshold of 100, after the host has been infected with the sensitive strain. 10^7 runs of simulations are performed to estimate $p_e(c)$. The parameter β is the same as in the agent-based model. To obtain analytical expressions for the recovery rates, we fit the function

$$\gamma(c) = a \cdot \frac{\left(\frac{c}{c_0}\right)^b}{1 + \left(\frac{c}{c_0}\right)^b} + v. \tag{S3.3}$$

to the data obtained from the within-host simulations. For $\gamma_S(c)$, we obtain $a = 0.076$, $b = 10.4$, $c_0 = 0.30$, $v = 0.11$. For $\gamma_R(c)$, we obtain $a = 0.072$, $b = 20.3$, $c_0 = 0.60$, $v = 0.11$. To fit the probability of within-host evolution of resistance, we use the function

$$p(c) = a_1 e^{-\left(\frac{c-b_1}{c_1}\right)^2} + a_2 e^{-\left(\frac{c-b_2}{c_2}\right)^2} + d, \tag{S3.4}$$

yielding $a_1 = 0.0079$, $b_1 = 0.42$, $c_1 = 0.10$, $a_2 = 0.0015$, $b_2 = 0.10$, $c_2 = 0.25$, $d = 0.00083$. Fits are performed in R.

S3.2 Comparison of the basic reproductive number between both models

The basic reproductive number R_0 (defined as the mean number of secondary cases caused by one sensitively infected individual in a fully susceptible population) turns out to be identical in both models (Fig IV).

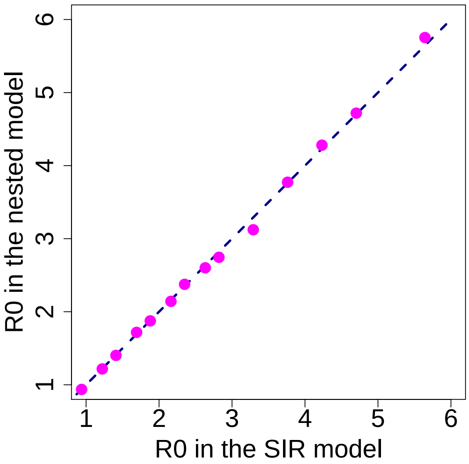

Figure IV. Comparison of the basic reproductive numbers measured for the agent-based and the SIR models. For the nested model, each data point averages the number of secondary infections that a single individual infected with the wild-type strain caused in an otherwise fully-susceptible population of $10,000$ individuals, for a transmission coefficient β, over 300 simulation runs. For the SIR model, each data point calculates the R_0 corresponding to the transmission coefficient β, following the formula $R_0 = \beta S_0/\gamma_S(0)$. As in the rest of our analysis, $\gamma_S(0) = 0.1063$ days^{-1}. The dashed blue line corresponds to $y = x$.

S3.3 Comparison of the disease burden to results from agent based simulations

The SIR model does not allow for co-infection with both strains or for superinfection of already-infected hosts. Moreover, the model allows for transitions between the two compartments for infected individuals in one direction only: from the compartment of hosts infected with the sensitive-to-treatment strain to the compartment of hosts infected with the resistant-to-treatment strain. Despite these strong simplifying assumptions, the results for the disease burden are similar in both models (see Fig V) which justifies the introduction of such a simpler model for the analysis of our results (even though the “valley” is more pronounced in the deterministic SIR model).

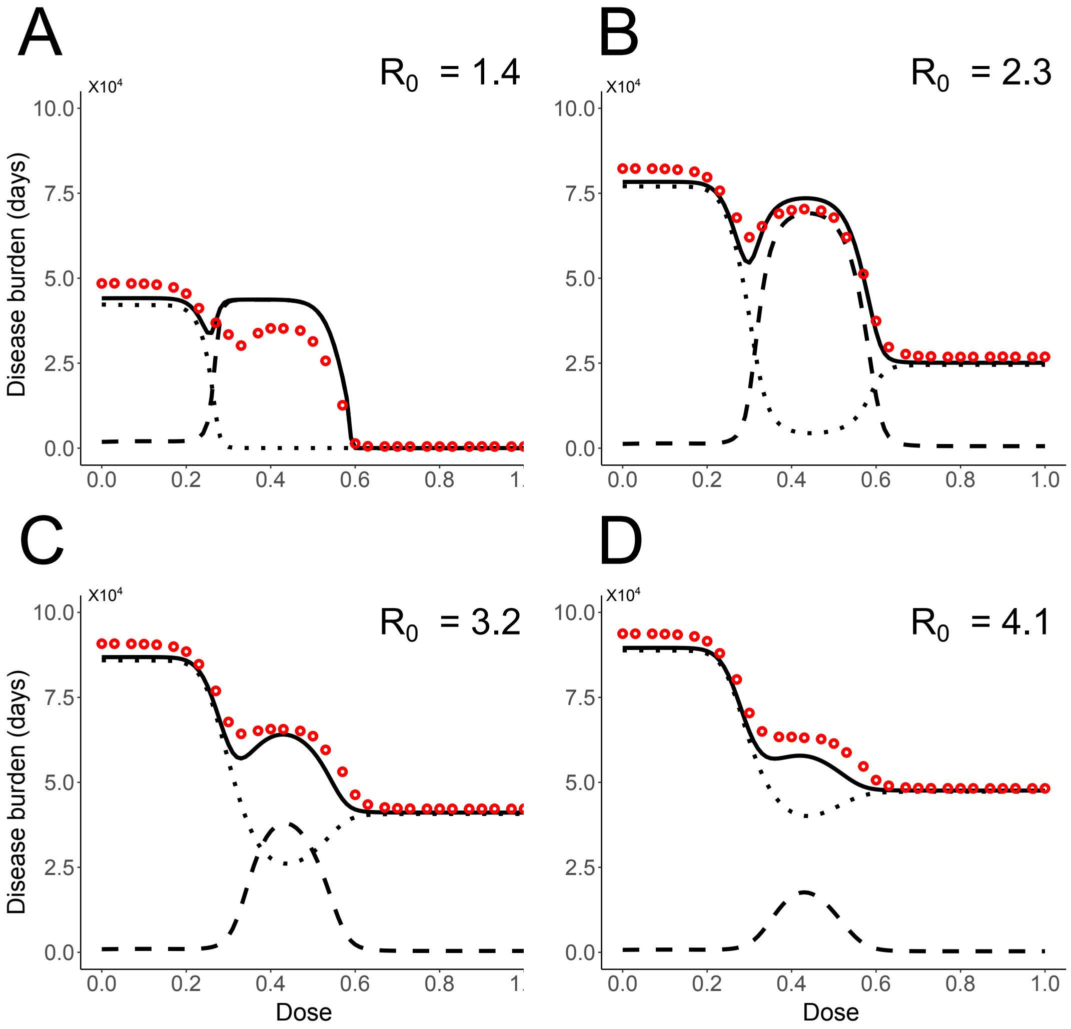

Figure V. Comparison of the disease burden in the SIR and nested models. For the nested model (red dots), each dot represents the mean value over 800 to 4000 runs of simulation. The transmission coefficients are given by: (**A**) $\beta = 1.5 \times 10^{-5}$; (**B**) $\beta = 2.5 \times 10^{-5}$; (**C**) $\beta = 3.5 \times 10^{-5}$; (**D**) $\beta = 4.5 \times 10^{-5}$.

S3.4 Comparison to a stochastic SIR model

For the disease burden, we only consider large outbreaks where the number of pathogens are high. These large numbers allow us to use a deterministic SIR model. Since the R_0 is similar in the SIR model and the agent-based model, we find good agreement between both models.

In other cases, stochastic effects matter, and we therefore briefly discuss the relation of the agent-based model to a stochastic SIR model.

S3.4.1 Comparison of the distribution of the number of secondary cases

The number of secondary cases caused by a sensitively infected individual in a fully susceptible population is a random variable, which we denote by Y in the following. Since the probability of resistance emergence is small for any one individual, we ignore it in the following discussion for simplicity.

In the SIR model, the waiting times for the next event (recovery or infection) are exponentially distributed. This implies that the random variable Y is geometrically distributed

with parameter

$$p = \frac{\gamma_S(c)}{\beta S_0 + \gamma_S(c)}, \tag{S3.5}$$

i.e., the probability that there are k secondary infections is given by

$$P_{\mathrm{SIR}}(Y = k) = (1-p)^k p. \tag{S3.6}$$

For the nested agent-based model, the recovery time is not exponentially distributed, and the distribution of the number of secondary cases differs from a geometric distribution (see Fig VI).

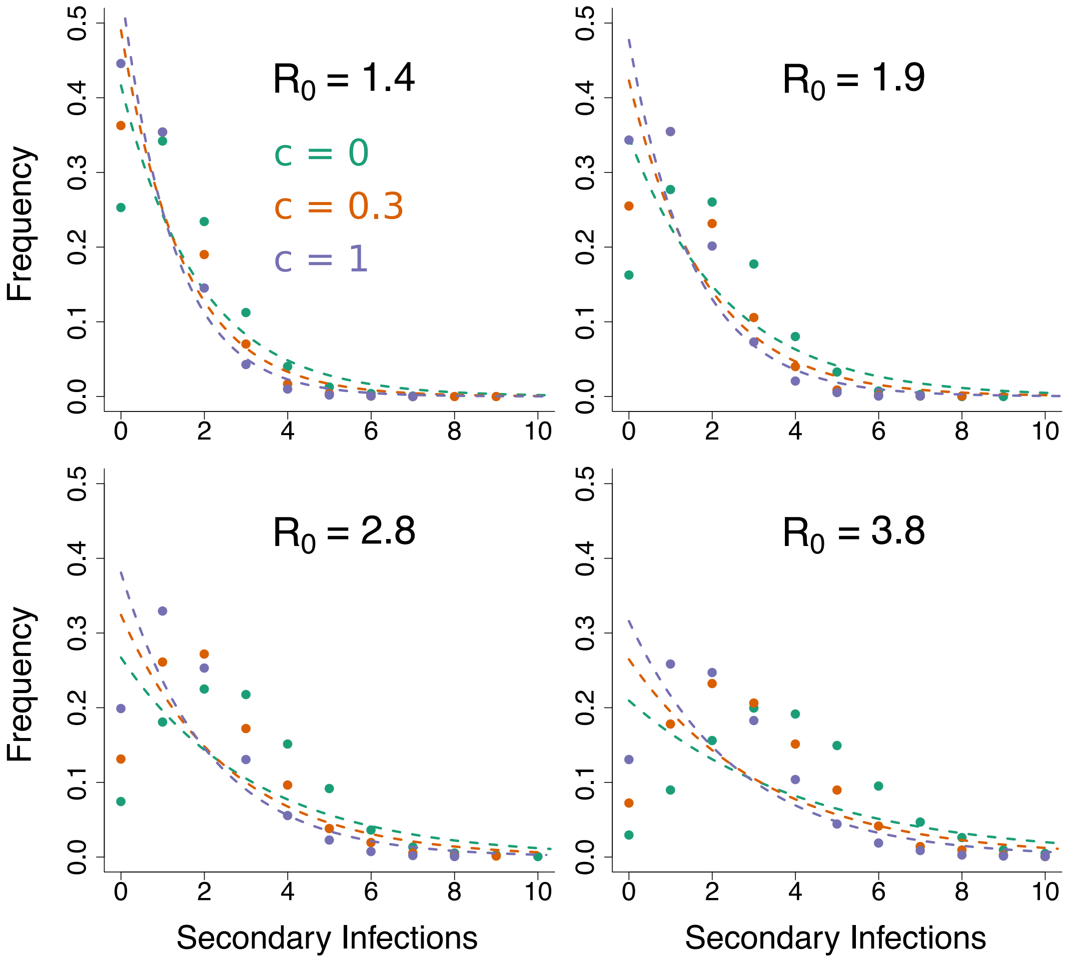

Figure VI. Distribution of the number of secondary cases for an infectious individual in an otherwise fully-susceptible population, in the nested model. The distributions are shown for four different transmission coefficients (one per panel) and for three different treatment doses. Colours are used to distinguish between the doses. The dashed lines represent a geometric distribution of mean identical to its corresponding distribution. This would be the expected distribution for a stochastic SIR model with parameters similar to the nested model presented here. The R_0 indicated in each panel is the basic reproduction number in the absence of treatment associated with the transmission coefficient used there. From left to right and top to bottom: $\beta = 1.5 \times 10^{-5}$, $\beta = 2.0 \times 10^{-5}$, $\beta = 3.0 \times 10^{-5}$, $\beta = 4.0 \times 10^{-5}$. Between 1700 and 2100 runs of simulation were performed per transmission coefficient and treatment dose.

S3.4.2 The outbreak probability in a stochastic SIR model and in the nested agent-based model

As long as infected individuals are rare in the population, the dynamics are highly stochastic and the distribution of secondary cases is important. It is therefore not surprising that the outbreak probability differs quantitatively between the agent based model and a stochastic SIR model. As we argue in the main text and discuss in more detail below, the possibility of resistance evolution usually does not influence the outbreak probability. It is therefore sufficient to focus on patients that are infected with the sensitive strain.

The outbreak probability in the SIR model can be obtained using a branching process approach based on Eq (S3.6) (or directly on a continuous-time birth-death process), leading to the well-known result

$$P_{\text{outbreak}}(c) = 1 - \frac{1}{R_0(c)}. \tag{S3.7}$$

For $R_0 = 2.3$ and $c = 0$, this predicts an outbreak probability of about 57%, which is considerable lower than in the agent based model ($\approx$ 85%, see Fig 4). However, both models agree in their qualitative predictions. In particular, Eq (S3.7) also predicts that high dose treatment reduces the outbreak probability more strongly for low R_0 than for high R_0.

S4: SIR model with partial treatment coverage

We extend the SIR model by considering that only a fraction of the infected population receives the treatment. Upon infection, the newly infected individuals are separated in two compartments: a fraction f gets treatment (superscript T) and a fraction $1-f$ is untreated (superscript U). Here, $\gamma_S(0)$, $\gamma_S(c)$ are respectively the recovery rates of the untreated and treated infected with the susceptible pathogen; $\gamma_R(0)$, $\gamma_R(c)$ are the recovery rates of the untreated and treated infected with the resistant pathogen; $p_e(0)$, $p_e(c)$ are the probability of emergence for untreated and treated individuals. The dynamics of the epidemics with partial coverage is described by the system of differential equations:

$$\dot{S} = -\beta S(I_S^U + I_S^T + I_R^U + I_R^T) \tag{S4.8a}$$

$$\dot{I_S^U} = \beta S(I_S^U + I_S^T)(1-f) - \gamma_S(0) I_S^U \tag{S4.8b}$$

$$\dot{I_S^T} = \beta S(I_S^U + I_S^T)f - \gamma_S(c) I_S^T \tag{S4.8c}$$

$$\dot{I_R^U} = \beta S(I_R^U + I_R^T)(1-f) - \gamma_R(0) I_R^U + p_e(0)\gamma_S(0) I_S^U \tag{S4.8d}$$

$$\dot{I_R^T} = \beta S(I_R^U + I_R^T)f - \gamma_R(c) I_R^T + p_e(c)\gamma_S(c) I_S^T \tag{S4.8e}$$

$$\dot{R} = (1-p_e(0))\gamma_S(0) I_S^U + (1-p_e(c))\gamma_S(c) I_S^T + \gamma_R(0) I_R^U + \gamma_R(c) I_R^T. \tag{S4.8f}$$

The total burden is measured as

$$B = \int_0^\infty (I_S^U(t) + I_S^T(t) + I_R^U(t) + I_R^T(t))\mathrm{d}t. \tag{S4.9}$$

S5: Emergence of resistance in an endemic disease

We here consider an SIR model with birth and death (immigration and emigration), in which the disease can persist rather than going extinct due to a shortage of susceptibles as in the main text. Within this framework, we derive the hazard rate for the emergence of resistance. For simplicity, we assume that the population is in endemic equilibrium prior to the emergence of resistance.

S5.1 Complete treatment coverage and lifelong immunity

By adding birth of susceptible individuals at total rate Λ and death at per-capita rate δ to the model defined in Eq (3), we obtain:

$$
\begin{aligned}
\dot{S} &= \Lambda - \beta\, S\, (I_S + I_R) - \delta S, && \text{(S5.10a)}\\
\dot{I}_S &= \beta\, S\, I_S \quad - \gamma_S(c)\, I_S \ - \delta I_S, && \text{(S5.10b)}\\
\dot{I}_R &= p_e(c)\, \gamma_S(c)\, I_S \quad + \beta\, S\, I_R \quad - \gamma_R(c)\, I_R \ - \delta I_R, && \text{(S5.10c)}\\
\dot{R} &= (1 - p_e(c))\, \gamma_S(c)\, I_S \quad + \gamma_R(c)\, I_R \ - \delta R. && \text{(S5.10d)}
\end{aligned}
$$

We assume here that the death rate is the same for all individuals, independent of their status (susceptible, infected, recovered).

As in the main text, we denote by R_0 the basic reproductive number of the sensitive strain in a fully susceptible population, $R_0 = \frac{\beta S_0}{\gamma_S(c)+\delta} = \frac{\beta \Lambda}{(\gamma_S(c)+\delta)\delta}$. We assume that resistance has not yet emerged ($I_R = 0$). For $R_0 > 1$, an endemic equilibrium with $I_S > 0$ exists, and we focus on this case. In equilibrium:

$$
\begin{aligned}
S^{\text{eq}}(c) &= \frac{\delta + \gamma_S(c)}{\beta}, && \text{(S5.11a)}\\
I_S^{\text{eq}}(c) &= \frac{\Lambda}{\delta + \gamma_S(c)} - \frac{\delta}{\beta}, && \text{(S5.11b)}\\
R^{\text{eq}}(c) &= \frac{\Lambda}{\delta} - S^{\text{eq}}(c) - I_S^{\text{eq}}(c). && \text{(S5.11c)}
\end{aligned}
$$

We now consider the stochastic emergence of resistance. Instead of the deterministic system defined by Eq (S5.10), we study the corresponding stochastic system, where events happen at the respective rates. With this, resistant infections appear de novo at rate $p_e(c)\gamma_S(c)I_S^{\text{eq}}(c)$. Once there, resistance can either disappear after few infections or spread in the population. Importantly, even if superior to the sensitive strain under sufficiently strong treatment, the resistant strain can be lost from the population due to stochasticity. We therefore need to determine the probability that resistance becomes endemic. The basic reproductive number of the resistant strain, when the sensitive strain is at equilibrium, is given by

$$R_0^{r,\text{eq}}(c) = \frac{\beta S^{\text{eq}}(c)}{\gamma_R(c) + \delta}. \qquad \text{(S5.12)}$$

From the basic reproductive number, the establishment probability of the resistant strain, initially appearing in a single infection, can be obtained as

$$P_{\text{res}}(c) = \begin{cases} 1 - \frac{1}{R_0^{r,\text{eq}}(c)} & \text{if } R_0^{r,\text{eq}}(c) > 1, \\ 0 & \text{else,} \end{cases} \qquad \text{(S5.13)}$$

[see e.g. 2, p. 107]. The condition $R_0^{r,\text{eq}}(c) > 1$ is equivalent to $\gamma_R(c) < \gamma_S(c)$. Note that $P_{\text{res}}(c)$ is independent of β. The advantage of higher infectivity with higher β is exactly counterbalanced by a lower pool of targets of infection. Panels A and B in Fig VII show the establishment probability of the resistant strain $P_{\text{res}}(c)$ at the between-host level along with the probability of resistance emergence at the within-host level $p_e(c)$. For low doses, resistance cannot spread in the population since the pool of susceptibles is too low ($\gamma_S(0) < \gamma_R(0)$). For high doses, the resistant strain has a low establishment probability since it is only very slightly better than the sensitive strain. For intermediate doses, $P_{\text{res}}(c)$ displays a maximum just as the within-host probability of resistance. Both probabilities peak at similar doses. This essentially comes since the within-host growth rates of both strains (determining p_e) determine the recovery rates (which in turn determine P_{res}). The peak in p_e is close to doses where the difference in the growth rates of the two strains is largest and spread of resistance easiest (the higher number of *de novo* mutations for lower doses only has a small effect on

the location of the peak in the parameter ranges considered); the peak in P_{res} is where the difference in the recovery rates is largest.

Overall, we obtain the hazard rate for resistance emergence at the between host level as:

$$h_{\mathrm{res}}(c) = p_e(c)\gamma_S(c) I_S^{\mathrm{eq}}(c) P_{\mathrm{res}}(c). \tag{S5.14}$$

Fig VII shows the hazard rate and its components for two different values of R_0. For $R_0 = 1.75$, the number of individuals that are infected by the sensitive strain drops by more than 95% if a high dose is used, while this reduction is only $\approx$ 55% for $R_0 = 4$ (Panels B and F). For $R_0 = 4$, the rate of appearance of the resistant strain peaks at the same dose as the within-host probability of resistance, $p_e(c)$. As discussed before, the establishment probability of the resistant strain at the population level, $P_{\mathrm{res}}(c)$, peaks at a similar dose as well. Hence, there are no antagonistic effects between both components, and the hazard rate is highest at the same dose as $p_e(c)$. In contrast, for $R_0 = 1.7$, the sharp drop in susceptibles with increasing drug dosage strongly affects the rate of appearance of the double resistant strain, which therefore is highest for low doses. As a consequence, the hazard rate peaks at lower doses than p_e and P_{res}.

Ultimately, this implies that for low R_0, the peaks of the hazard rate and the probability of resistance emergence at the within-host level are shifted. There is hence a regime, where lowering the drug dose reduces the probability that resistance emerges within a given patient but increases the probability that resistance emerges and spreads at the between-host level.

The equilibrium population size of sensitively infected patients for a given dose c is given by

$$I^{\mathrm{eq}}(c) = \frac{\Lambda}{\delta + \gamma_S(c)} - \frac{\delta}{\beta} = \frac{\delta}{\beta}\left(R_0 - 1\right). \tag{S5.15}$$

The ratio between the number of sensitively infected patients at dose 0 and dose 1 is hence given by

$$F(R_0, \Gamma) = \frac{I^{\mathrm{eq}}(c=0)}{I^{\mathrm{eq}}(c=1)} = \frac{R_0(0) - 1}{R_0(1) - 1} = \frac{R_0 - 1}{\Gamma R_0 - 1} \tag{S5.16}$$

with

$$\Gamma = \frac{\gamma_S(0) + \delta}{\gamma_S(1) + \delta}.$$

The function F decreases in R_0 and in Γ. In other words, the relative difference in the equilibrium number of sensitively infected patients is large if R_0 or Γ are small. R_0 is small if (1) the transmission coefficient β is small (2) the population size S_0 is low (3) the natural recovery rate in the absence of drug is large. Γ is low if $\gamma_S(1) \gg \gamma_S(0)$. All these conditions make it likely that the peak in the hazard rate is at a lower dose than the peak in the within-host probability of resistance. (Note that the natural death rate δ has a double-edged effect: it decreases R_0 but increases Γ. However, δ is not the primary focus here.)

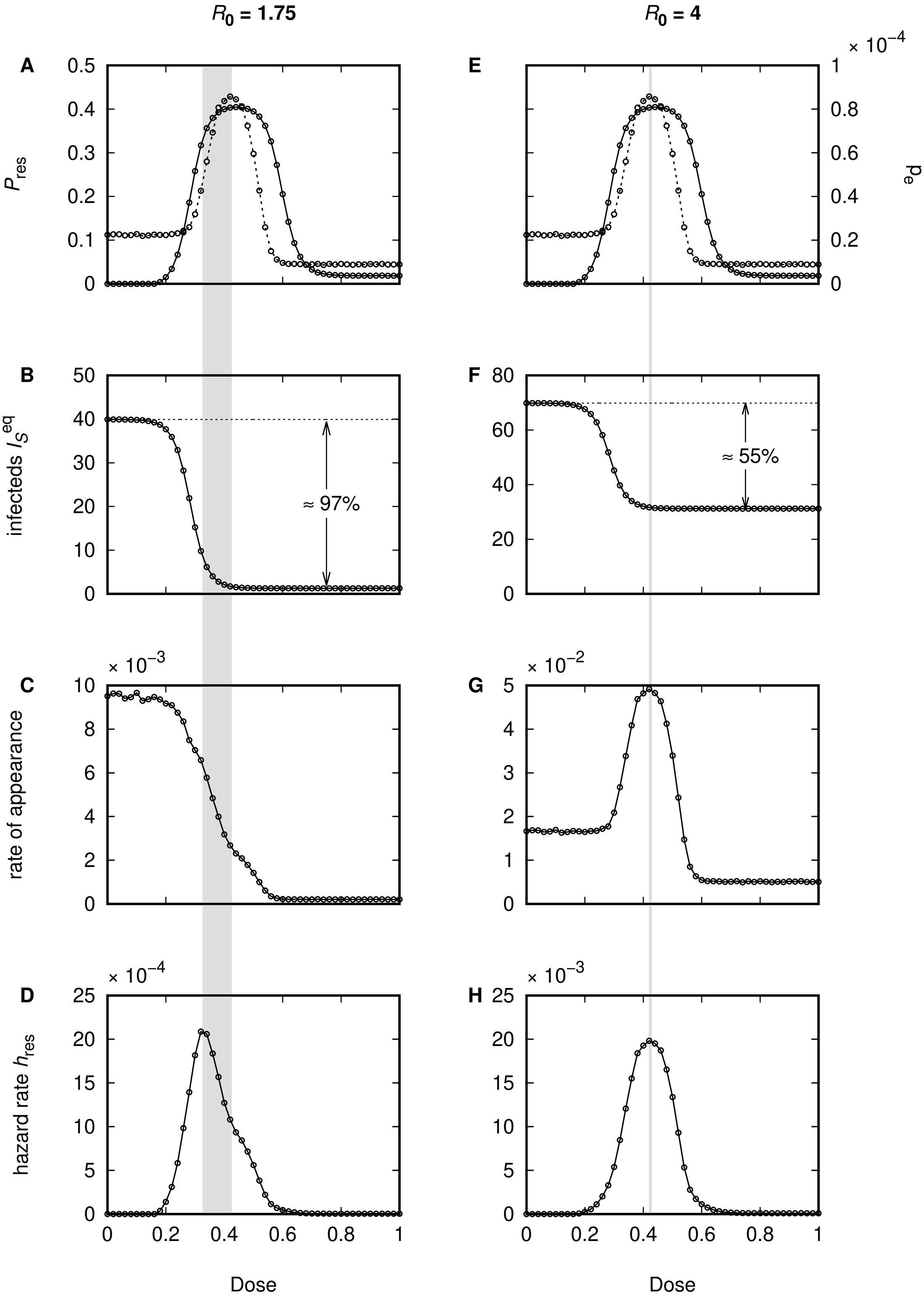

Figure VII. Emergence of resistance in an SIR model with birth and death as defined in Eq (S5.10) **for two different values of** R_0**.** (**A**) and (**E**): Probability of establishment of the resistant strain in the population (solid line) and probability of within-host resistance (dotted line). (**B**) and (**F**): Number of individuals infected by the sensitive strain at endemic equilibrium before resistance has emerged. (**C**) and (**G**): Rate of appearance of the resistant strain in the population. (**D**) and (**H**): Hazard rate of resistance emergence (combining de novo appearance and spread) at the population level. The gray bars illustrate the shift and alignment, respectively, in the peaks between the hazard rate and the probability of within-host resistance. All quantities got evaluated at discrete points (denoted by the symbols), for which we had obtained simulation results for the within-host probability of resistance. Lines are included for readability. Parameters: $\Lambda = 10$, $\delta = 10/S_0 = 0.001$. The within-host parameters are chosen as in the main text.

S5.2 Waning immunity (SIRS model)

Up to now, we have considered life-long immunity. We here discuss how results change if immunity is lost at rate q:

$$\begin{aligned}
\dot{S} &= \Lambda - \beta\, S\, (I_S + I_R) - \delta S + q\, R, && \text{(S5.17a)}\\
\dot{I}_S &= \beta\, S\, I_S - \gamma_S(c)\, I_S - \delta I_S, && \text{(S5.17b)}\\
\dot{I}_R &= p_e(c)\, \gamma_S(c)\, I_S + \beta\, S\, I_R - \gamma_R(c)\, I_R - \delta I_R, && \text{(S5.17c)}\\
\dot{R} &= (1-p_e(c))\, \gamma_S(c)\, I_S + \gamma_R(c)\, I_R - \delta R - q\, R. && \text{(S5.17d)}
\end{aligned}$$

This assumes that q is independent of dose (it is, however, conceivable that q is dose dependent).

In the endemic equilibrium before the emergence of resistance, we have

$$S^{\text{eq}}(c) = \frac{\delta + \gamma_S(c)}{\beta}, \tag{S5.18}$$

$$I_S^{\text{eq}}(c) = \frac{\delta + \gamma_S(c)}{\delta + \frac{\delta \gamma_S(c)}{\delta + q}} \times \left(\frac{\Lambda}{\delta + \gamma_S(c)} - \frac{\delta}{\beta} \right), \tag{S5.19}$$

$$R^{\text{eq}}(c) = \frac{\Lambda}{\delta} - S^{\text{eq}}(c) - I_S^{\text{eq}}(c). \tag{S5.20}$$

Note that the number of susceptibles does not change, i.e., the initial spread of resistance – once there – is not altered ($P_{\text{res}}(c)$ remains the same). However, the number of individuals infected with the sensitive strain increases. The *de novo* appearance of resistance in the population is hence increased. The hazard rate is obtained analogously to before.

In Fig VIII, one sees that the drug concentration at which the hazard rate is maximal does not strongly depend on the duration of immunity.

S5.3 Incomplete treatment coverage

Analogously to before, we can add birth and death to Eq (S4.8). In the endemic equilibrium before the emergence of resistance, we then have

$$\begin{aligned}
S^{\text{eq}}(c) &= \frac{(\gamma_S(c) + \delta)(\gamma_S(0) + \delta)}{\beta((1-f)\gamma_S(c) + f\gamma_S(0) + \delta)}, && \text{(S5.21a)}\\
I^{T,\text{eq}}(c) &= \frac{f\Lambda}{\gamma_S(c) + \delta} - \frac{f\delta(\gamma_S(0) + \delta)}{\beta((1-f)\gamma_S(c) + f\gamma_S(0) + \delta)}, && \text{(S5.21b)}\\
I^{U,\text{eq}}(c) &= \frac{(1-f)\Lambda}{\gamma_S(0) + \delta} - \frac{(1-f)\delta(\gamma_S(c) + \delta)}{\beta((1-f)\gamma_S(c) + f\gamma_S(0) + \delta)}, && \text{(S5.21c)}\\
R^{\text{eq}}(c) &= \frac{\Lambda}{\delta} - S^{\text{eq}}(c) - I^{T,\text{eq}}(c) - I^{U,\text{eq}}(c). && \text{(S5.21d)}
\end{aligned}$$

In treated individuals, resistance appears at rate $p_e(c)\gamma_S(c)I_S^{T,\text{eq}}(c)$, and in untreated individuals at rate $p_e(0)\gamma_S(0)I_S^{U,\text{eq}}(c)$. The establishment probability of the resistant strain differs, depending on whether it appeared in a treated or in an untreated individual. We denote the respective probabilities by $P_{\text{est}}^T(c)$ and $P_{\text{est}}^U(c)$. In order to derive these establishment probabilities, we approximate the initial phase of spread of resistance at the population level by a two-type branching process, where one type corresponds to treated and the other to untreated infections. We denote the corresponding extinction probabilities by $Q^T(c)$ and $Q^U(c)$ (we give a derivation of $Q^T(c)$ and $Q^U(c)$ below). We can then approximate $P_{\text{est}}^T(c) \approx 1 - Q^T(c)$ and $P_{\text{est}}^U(c) \approx 1 - Q^U(c)$.

With this, we obtain for the hazard rate

$$h_{\text{res}}(c) = p_e(c)\gamma_S(c)I_S^{T,\text{eq}}(c)P_{\text{est}}^T(c) + p_e(0)\gamma_S(0)I_S^{U,\text{eq}}(c)P_{\text{est}}^U(c). \tag{S5.22}$$

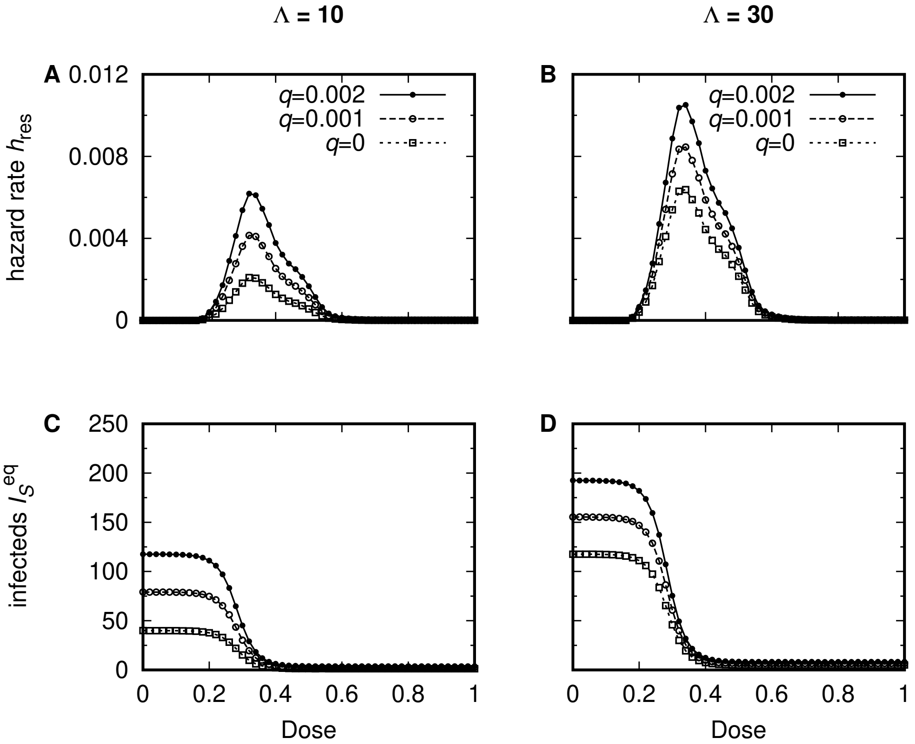

Figure VIII. Emergence of resistance in an SIR model with birth and death and waning immunity for two different birth/immigration rates and $R_0 = 1.75$. Top row (**A-B**): hazard rate as a function of treatment dose. Bottom row (**C-D**): number of infecteds with the sensitive strain as a function of treatment dose. Panels **A-C**: $\Lambda = 10$, panels **B-D**: $\Lambda = 30$. All quantities got evaluated at discrete points (denoted by the symbols), for which we had obtained simulation results for the within-host probability of resistance. Lines are included for readability. The within-host parameters are chosen as in the main text.

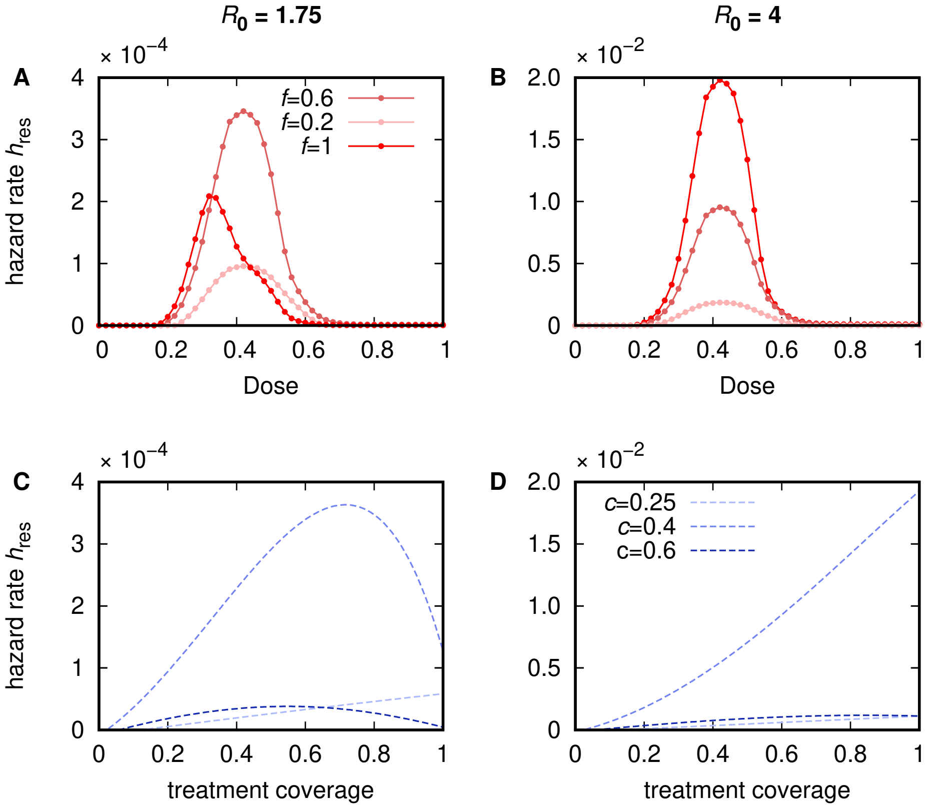

Figure IX. Emergence of resistance in an SIR model with birth and death and incomplete treatment coverage for two different values of R_0. Top row (**A-B**): hazard rate as a function of treatment dose. Bottom row (**C-D**): hazard rate as a function of treatment coverage. In panels **A-C**, $R_0 = 1.75$, in panels **B-D**, $R_0 = 4$. The hazard rates got evaluated at discrete points (denoted by the symbols), for which we had obtained simulation results for the within-host probability of resistance. Lines are included for readability. Parameters: $\Lambda = 10$, $\delta = 10/S_0 = 0.001$. The within-host parameters are chosen as in the main text.

Fig IX shows the hazard rate for two different values of R_0. We see that for small R_0, the hazard rate is largest for intermediate treatment coverage and intermediate doses. This is the regime where competition with the sensitive strain is released while the "resistance input" is high.

We now derive the extinction probabilities $Q^T(c)$ and $Q^U(c)$ of a two-type branching process that is founded by a treated and an untreated resistant infection, respectively. An infected individual fails to cause a secondary infection with probability $\frac{\delta+\gamma_R(c)}{\beta S^{\text{eq}}(c)+\delta+\gamma_R(c)}$ if treated and with probability $\frac{\delta+\gamma_R(0)}{\beta S^{\text{eq}}(c)+\delta+\gamma_R(0)}$ if untreated (this is the probability that it recovers or dies before it infects anybody). In that case, the resistant strain goes extinct immediately. The probability that at any time, the next event is an infection event (rather than death) is given by $\frac{\beta S^{\text{eq}}(c)}{\beta S^{\text{eq}}(c)+\delta+\gamma_R(c)}$ ($\frac{\beta S^{\text{eq}}(c)}{\beta S^{\text{eq}}(c)+\delta+\gamma_R(0)}$). The newly infected individual receives treatment with probability f and remains untreated with probability $1-f$. For the resistant strain to go extinct, both infected individuals need to fail to cause a resistant outbreak. Putting all together, it therefore holds:

$$Q^T(c) = \frac{\delta+\gamma_R(c)}{\beta S^{\text{eq}}(c)+\delta+\gamma_R(c)} + \frac{\beta S^{\text{eq}}(c)}{\beta S^{\text{eq}}(c)+\delta+\gamma_R(c)}(fQ^T(c)+(1-f)Q^U(c))Q^T(c), \quad \text{(S5.23a)}$$

$$Q^U(c) = \frac{\delta+\gamma_R(0)}{\beta S^{\text{eq}}(c)+\delta+\gamma_R(0)} + \frac{\beta S^{\text{eq}}(c)}{\beta S^{\text{eq}}(c)+\delta+\gamma_R(0)}(fQ^T(c)+(1-f)Q^U(c))Q^U(c). \quad \text{(S5.23b)}$$

The equations can be solved to give three pairs of solutions. Following the general theory of branching processes, the extinction probability is given by the solution within the unit cube that is closest to the origin [1].

Solution 1 is given by

$$Q_1^T(c) = Q_1^U(c) = 1. \tag{S5.24}$$

Solution 2 is given by:

$$\begin{aligned} Q_2^T(c) = &\frac{\gamma_R(c)+\delta}{2fS^{\text{eq}}(c)\beta(\gamma_R(c)-\gamma_R(0))} \\ &\times \left(\beta S^{\text{eq}}(c)+\gamma_R(c)-\gamma_R(0)+\sqrt{(\beta S^{\text{eq}}(c)+\gamma_R(c)-\gamma_R(0))^2-4fS^{\text{eq}}(c)\beta(\gamma_R(c)-\gamma_R(0))}\right), \end{aligned} \tag{S5.25a}$$

$$\begin{aligned} Q_2^U(c) = &\frac{\gamma_R(0)+\delta}{-2(1-f)S^{\text{eq}}(c)\beta(\gamma_R(c)-\gamma_R(0))} \\ &\times \left(\beta S^{\text{eq}}(c)-\gamma_R(c)+\gamma_R(0)+\sqrt{(\beta S^{\text{eq}}(c)+\gamma_R(c)-\gamma_R(0))^2-4fS^{\text{eq}}(c)\beta(\gamma_R(c)-\gamma_R(0))}\right). \end{aligned} \tag{S5.25b}$$

Solution 3 is given by:

$$Q_3^T(c) = \frac{2(\gamma_R(c)+\delta)}{\beta S^{\text{eq}}(c)+\gamma_R(c)-\gamma_R(0)+\sqrt{(\beta S^{\text{eq}}(c)+\gamma_R(c)-\gamma_R(0))^2-4fS^{\text{eq}}(c)\beta(\gamma_R(c)-\gamma_R(0))}}, \tag{S5.26a}$$

$$Q_3^U(c) = \frac{2(\gamma_R(0)+\delta)}{\beta S^{\text{eq}}(c)-\gamma_R(c)+\gamma_R(0)+\sqrt{(\beta S^{\text{eq}}(c)+\gamma_R(c)-\gamma_R(0))^2-4fS^{\text{eq}}(c)\beta(\gamma_R(c)-\gamma_R(0))}}. \tag{S5.26b}$$

Since

$$\begin{aligned} &\beta S^{\text{eq}}(c)-\gamma_R(c)+\gamma_R(0)+\sqrt{(\beta S^{\text{eq}}(c)+\gamma_R(c)-\gamma_R(0))^2-4fS^{\text{eq}}(c)\beta(\gamma_R(c)-\gamma_R(0))} \\ &= \beta S^{\text{eq}}(c)-\gamma_R(c)+\gamma_R(0)+\sqrt{(\beta S^{\text{eq}}(c)-\gamma_R(c)+\gamma_R(0))^2+4(1-f)S^{\text{eq}}(c)\beta(\gamma_R(c)-\gamma_R(0))} \\ &> \beta S^{\text{eq}}(c)-\gamma_R(c)+\gamma_R(0)+\sqrt{(\beta S^{\text{eq}}(c)-\gamma_R(c)+\gamma_R(0))^2} \geq 0, \end{aligned} \tag{S5.27}$$

we know that $Q_2^U(c) < 0$. Solution 2 hence does not lie within the unit cube. For solution 3, it holds that $Q_3^T(c) > 0$ and $Q_3^U(c) > 0$. We hence obtain for the extinction probabilities of the branching process:

$$\begin{pmatrix} Q^T(c) \\ Q^U(c) \end{pmatrix} = \begin{cases} \begin{pmatrix} Q_3^T(c) \\ Q_3^U(c) \end{pmatrix} & \text{if } Q_3^T(c) < 1 \text{ and } Q_3^U(c) < 1, \\ \begin{pmatrix} 1 \\ 1 \end{pmatrix} & \text{otherwise.} \end{cases} \tag{S5.28a}$$

S6: Additional figures showing a trade-off between minimising resistance at the two scales

In Fig 3, we see that trade-offs appear for low transmission coefficients β. As discussed in the main text, similar trade-offs appear for low S_0 and for a strong treatment efficacy. This can be seen in Fig X. In all these cases, the number of transmission events of the resistant strain peaks at lower doses than the within-host probability of resistance. As pointed out in the main text, for a very bad immune system, the opposite trade-off can appear. This is shown in Fig XI. Note, however, the extremely high probability of within-host resistance for this parameter regime.

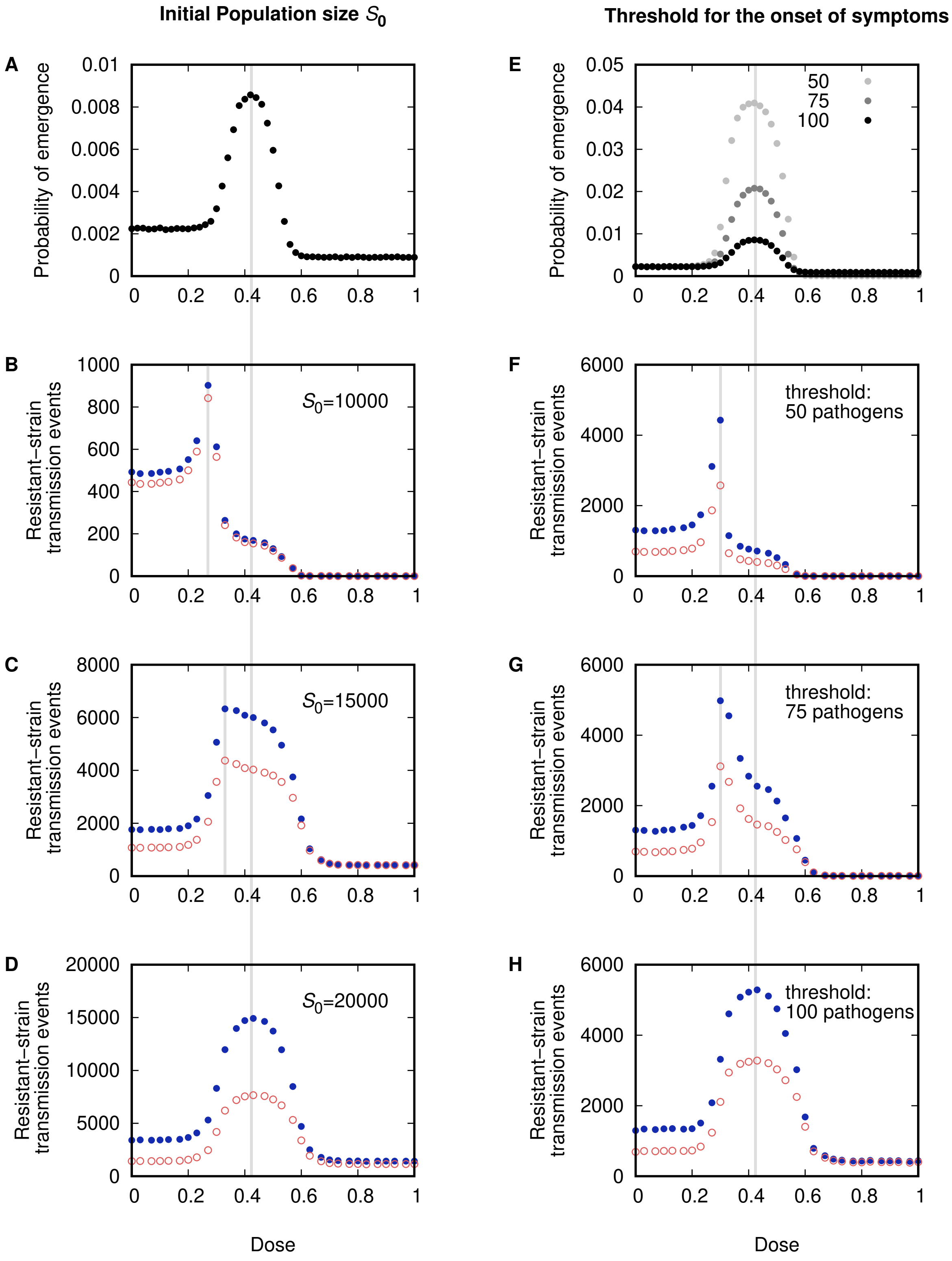

Figure X. The effect of the population size S_0 and the threshold for the onset of symptoms on the optimal drug dose at the between-host level in comparison to the within-host level. For Panels A-D, we used $\beta = 1.5 \times 10^{-5}$; for Panels E-H, we used $\beta = 2.5 \times 10^{-5}$.

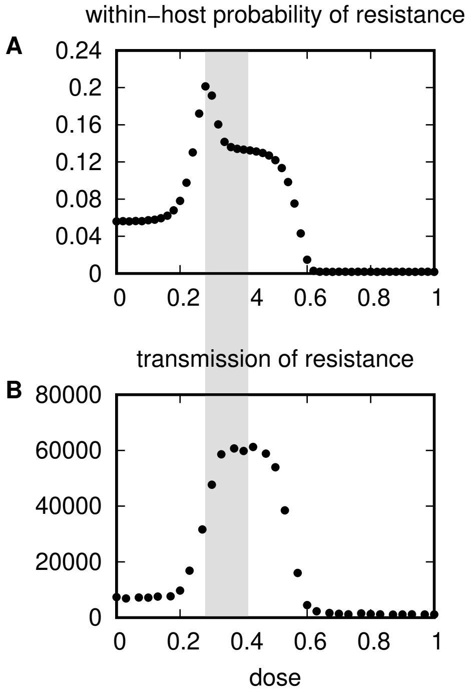

Figure XI. Comparison of the optimal dose determined through the within-host probability of resistance (A) or through the number of transmission events of the resistant strain during an epidemic (B). In the plot, the decay rate of the immune system δ is set to 0.05 days^{-1} compared to $\delta = 0.01$ days^{-1}. The transmission coefficient is $\beta = 4.5 \times 10^{-5}$ days^{-1}.

S7: Alternative measures of the overall disease impact

S7.1 Symptomatic disease burden

In the main text, we consider the disease burden based on the number of days that individuals spend being infectious. This allows for a direct comparison of results from the agent-based model to results from the SIR model. However, depending on context, the number of days during which people show symptoms might be more relevant. We therefore considered this measure as well (Fig XII). We see that it shows a very similar behavior.

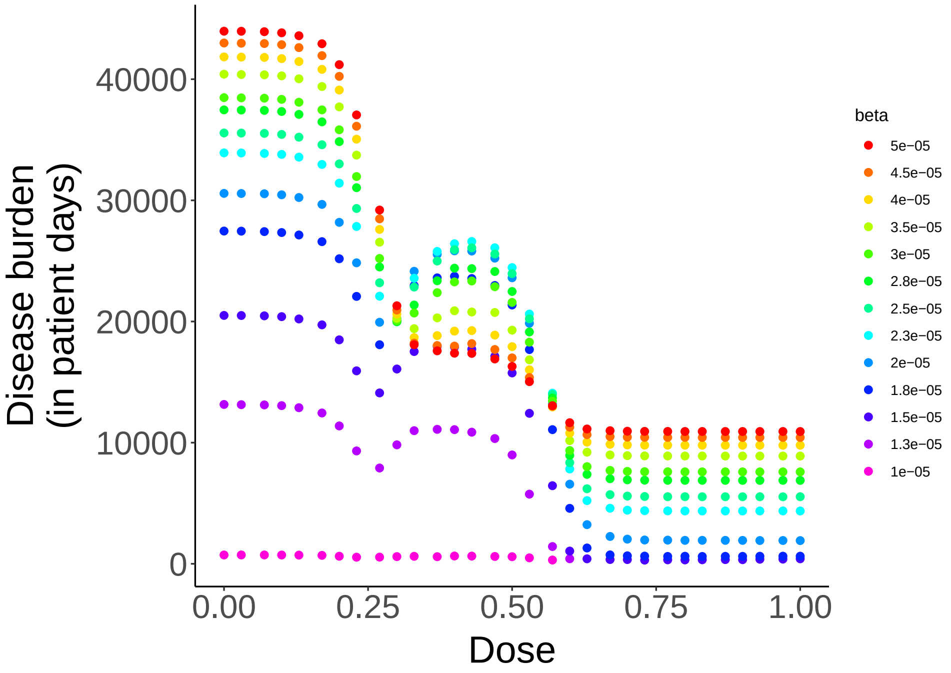

Figure XII. Total number of days spent suffering from symptoms by individuals of the infected population, as a function of the treatment dose, for various transmission coefficients. Mean for 4600 to 18000 simulation runs per data point. The confidence intervals are not shown as they are too small to be clearly seen on the plot. This measure of the disease burden behaves very similarly to the burden measure introduced in the main text.

S7.2 The "valley" in the total number of infecteds

Another measure of treatment success is the total number of infecteds during the epidemic. Fig XIII shows that the number of infecteds also shows a minimum followed by a maximum for intermediate doses. However, contrarily to the disease burden, the total number of infected hosts as a function of R_0 shows no minimum at intermediary doses.

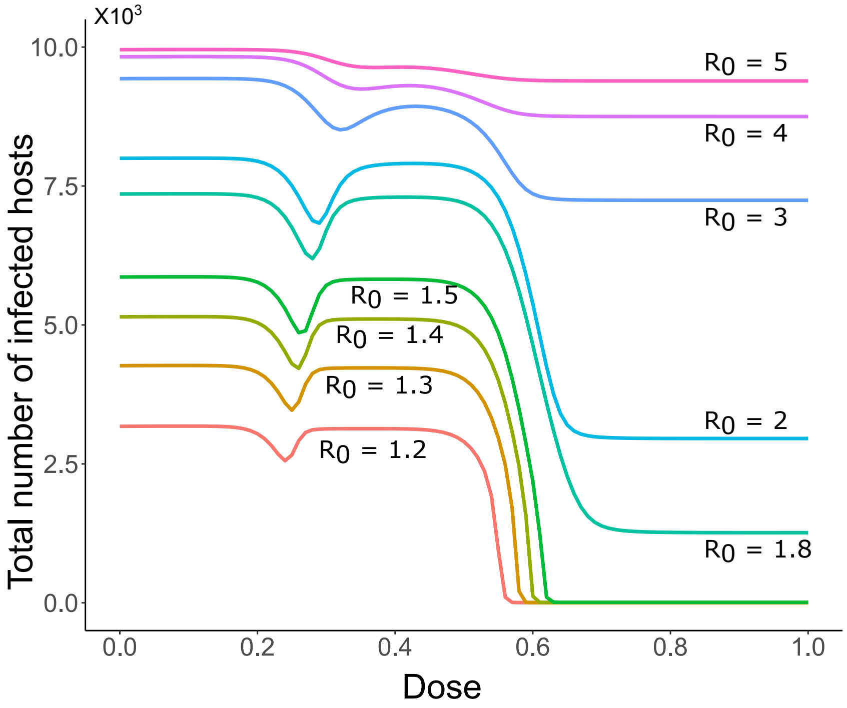

Figure XIII. Number of infected hosts during the course of the epidemic as a function of the treatment dose, for various R_0.

S8: The "valley" in the disease burden and in the total number of infected individuals

S8.1 The influence of p_e on the valley

To investigate the influence of the within-host emergence of resistance on the disease burden, we analyzed the SIR model with various values of p_e.

We simulated the SIR model with the recovery rates γ_S and γ_R estimated from the within-host simulations (see section S3.1), but we set different functions for how p_e varies with the dose. We considered four cases : p_e estimated from the within-host simulations, p_e divided by 20, p_e multiplied by 10, and p_e set to a constant value (2.010^{-3}), and we measured the disease burden when varying the dose.

We observed that p_e has little influence on the existence of a valley (Fig XIV). In particular, there is almost no difference between a constant p_e and the p_e estimated from within-host simulations. However, the lower p_e, the deeper the valley.

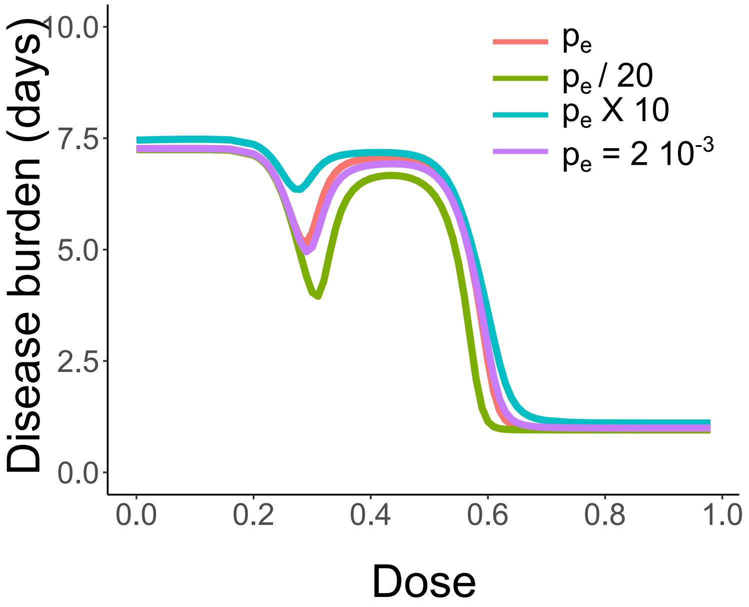

Figure XIV. Influence of p_e on the existence of a valley. The disease burden is plotted as a function of dose for $R_0 = 2$, with parameters γ_S and γ_R given in section S3.1. Four scenarios are represented for the within-host probability of emergence of resistance: the probability $p_e(c)$ given in section S3.1 (red), $\frac{1}{20}p_e(c)$ (green), $10 \times p_e(c)$ (blue), and the constant value $p_e = 2.010^{-3}$ (purple).

S8.2 Simplification: Two sequential epidemics

In our model, the resistant strain is absent initially and needs to arise *de novo* within a host before it can spread in the population. The spread of the resistant strain therefore occurs with a time delay compared to the spread of the sensitive strain. We here consider the extreme case, where the two epidemics caused by the sensitive and the resistant strains respectively occur strictly one after the other (cf. [3]): the first epidemic is caused by the sensitive strain, the second epidemic is caused by the resistant strain, spreading among the "leftover" susceptibles. This a drastic simplification of the true dynamics but we will see below that the simplified model still reproduces the general pattern in the total number of infecteds as observed in the full model.

The number of infecteds at the end of the sensitive epidemic is the solution to the equation

$$R_S^\infty = S_0 \left(1 - e^{-\frac{\beta}{\gamma_S(c)} R_S^\infty}\right), \tag{S8.29}$$

see e.g. [4, p. 28]. Likewise, the number of infecteds at the end of the subsequent resistant epidemic is given through

$$R_R^\infty = (S_0 - R_S^\infty) \left(1 - e^{-\frac{\beta}{\gamma_R(c)} R_R^\infty}\right). \tag{S8.30}$$

The total number of infecteds once both epidemics are over is the sum

$$R^\infty = R_S^\infty + R_R^\infty. \tag{S8.31}$$

Figure XV shows this for three values of R_0.

The basic reproductive number of the resistant strain after the first epidemic is $R_0^{Res}(c) = \frac{\beta S_0(c)}{\gamma_R(c)}$, where $S_0(c)$ is the number of leftover susceptibles. The resistant strain hence needs more than $\frac{\gamma_R(c)}{\beta}$ susceptible individuals left to be able to spread. In Fig XV, the total

number of infecteds is lowest at the concentration $c^\star$ for which $S_0 - R_S^\infty(c^\star) = \frac{\gamma_R(c^\star)}{\beta}$. For higher concentrations, the sensitive strain does not infect sufficiently many individuals to generate herd immunity that would prevent a secondary epidemic by the resistant strain ("overshooting", see [3]). The critical concentration $c^\star$ is shifted to the right with increasing R_0 (compare Fig XVA and B). If R_0 is too large, no such $c^\star$ exists because at all concentrations sufficiently many individuals gain immunity through infection with the sensitive strain. We also observe that for low R_0 and intermediate doses, a higher proportion of all infections is caused by the resistant strain than for high R_0 (cf. [5] for a similar observation on the effect of R_0 on the proportion of resistant cases in a model on optimal treatment coverage).

In the following, we derive an approximate formula for when a "valley" exists in the total number of infecteds. It holds in the general case that:

$$R_0 = \beta N_0/\gamma.$$

Therefore, we have at dose $c = c^\star$:

$$R_0^{Res}(c^\star) = \beta(S_0 - R_\infty^S(c^\star))/\gamma_R(c^\star)$$

We now use the above observation and put $R_0^{Res}(c^\star) = 1$. We furthermore assume that the resistant strain is not affected by the drug at concentration $c^\star$, which is a plausible assumption and can also be seen from Fig XV and the recovery rates shown in Fig 2. We therefore set $\gamma_R(c^\star) = \gamma_R(0) = \alpha\gamma_S(0) \equiv \alpha\gamma_0$. With these rearrangements, we obtain:

$$R_\infty^S(c^\star) = \frac{\gamma_0}{\beta}(R_0 - \alpha)$$

with R_0 the basic reproduction number of the sensitive strain in the absence of treatment, $R_0 = \beta S_0/\gamma_0$. It also holds true for all doses that:

$$R_\infty^S(c) = S_0(1 - e^{-\frac{\beta}{\gamma_S(c)}R_\infty^S}(c))$$

Using the two equations above, we have at the valley:

$$\frac{\gamma_0}{\beta}(R_0 - \alpha) = S_0(1 - e^{-\frac{\beta}{\gamma_S(c^\star)}\frac{\gamma_0}{\beta}(R_0-\alpha)}).$$

Thus,

$$\frac{\gamma_0}{\beta S_0}(R_0 - \alpha) = 1 - e^{-\frac{\gamma_0}{\gamma_S(c^\star)}(R_0-\alpha)}.$$

After rearrangement of terms, we get:

$$\frac{\alpha}{R_0} = e^{-\frac{\gamma_0}{\gamma_S(c^\star)}(R_0-\alpha)}.$$

Then,

$$\ln(\frac{\alpha}{R_0}) = -\frac{\gamma_0}{\gamma_S(c^\star)}(R_0 - \alpha).$$

And so,

$$\gamma_S(c^\star) = \frac{\gamma_0(R_0 - \alpha)}{\ln(R_0) - \ln(\alpha)} \approx \frac{\gamma_0(R_0 - 1)}{\ln(R_0)}. \tag{S8.32}$$

The approximation holds for α close to 1, i.e. if the fitness cost of the resistant strain is small. Since γ_S as a function of the dose c is generally strictly increasing (such as is the case of a sigmoid function), the value of $\gamma_S(c^\star)$ directly gives the value of $c^\star$, provided that such a dose exists. If it does not exist, then there is no valley for the set of parameters considered.

We can read off several observations from Eq (S8.32). For this, first note that $\frac{R_0-1}{\ln(R_0)}$ increases monotonically with R_0. We therefore see that the valley shifts to the right for

increasing R_0 as also observed in Fig XV. When R_0 becomes so large that $\frac{\gamma_0(R_0-1)}{\ln(R_0)} \geq \gamma_{\max} := \lim_{c\to\infty} \gamma_S(c)$, then no valley exists. Putting differently, a valley exists if

$$\frac{R_0 - 1}{\ln(R_0)} < \frac{\gamma_{\max}}{\gamma_0}, \tag{S8.33}$$

i.e., the stronger the maximal effect of the drug, the larger the R_0 for which we stop observing a valley. In section S8.3, we will confirm these observations in a formal analysis of the full model (considering the disease burden as in the main text).

From Eq (S8.33), we can calculate the maximal value of R_0 for which a valley can be observed. For the parameters used throughout the article, it holds that $\gamma_{\max}/\gamma_0 \approx 1.7$. With this, we obtain $R_0^{\max} \approx 2.7$, which is lower than the value of R_0 above which the valley disappears in the full deterministic SIR model (close to $R_0 = 4$, see Fig XIII). This is not surprising since in the full model, the epidemics caused by the sensitive and the resistant strain are overlapping. I.e., by the time the resistant strain starts spreading, the sensitive strain has not used up all susceptible hosts that it could use up on its own for a given R_0.

S8.3 Formal analysis of the full model and identification of the decisive composite parameters

S8.3.1 An equivalent SIR model with time-dependent recovery rate

Our objective in this section is to simplify the SIR model (3) and to analytically show the existence of a valley for certain sets of parameters. We transform the system of equations (3) by considering the new variables $I(t) = I_S(t) + I_R(t)$ and $\varphi(t) = \frac{I_S(t)}{I_S(t)+I_R(t)}$. We obtain the differential equation for φ

$$\begin{aligned}
\dot{\varphi} &= \frac{\dot{I}_S I - I_S \dot{I}}{I^2} \\
&= \frac{\beta S I_S - \gamma_S I_S}{I} - \frac{I_S}{I}\frac{\dot{I}}{I} \\
&= (\beta S - \gamma_S)\varphi - \varphi\Big(\beta S - (1-p_e)\gamma_S\varphi - \gamma_R(1-\varphi)\Big) \\
&= ((1-p_e)\gamma_S - \gamma_R)\varphi^2 - (\gamma_S - \gamma_R)\varphi.
\end{aligned} \tag{S8.34}$$

This is a Bernoulli equation, the solution of which depends on whether the coefficients are equal to 0 or not. With the condition $\varphi(0) = 1$, it can be solved and leads to the three possible solutions

$$\varphi(t) = \begin{cases} e^{-p_e\gamma_S t} \text{ if } \gamma_S = \dfrac{\gamma_R}{1-p_e} \\[2ex] \dfrac{1}{1+p_e\gamma_S t} \text{ if } \gamma_S = \gamma_R \\[2ex] \dfrac{1}{1+\frac{p_e\gamma_S}{\gamma_S-\gamma_R}\left(e^{(\gamma_S-\gamma_R)t}-1\right)} \text{ otherwise.} \end{cases}$$

If $p_e = 0$, φ is a constant equal to 1, and otherwise, goes asymptotically to 0 for $\gamma_S \geq \gamma_R$. In the case where $\gamma_S < \gamma_R$, the asymptotic value of φ is not 0 but

$$\lim_{t\to\infty} \varphi(t) = \frac{1}{1+\frac{p_e\gamma_S}{\gamma_R-\gamma_S}}. \tag{S8.35}$$

Considering $G(t) = (1-p_e)\gamma_S\varphi(t)+\gamma_R(1-\varphi(t))$, which can be regarded as the global recovery rate of the infected population, we obtain the SIR system of equations with a time-dependent

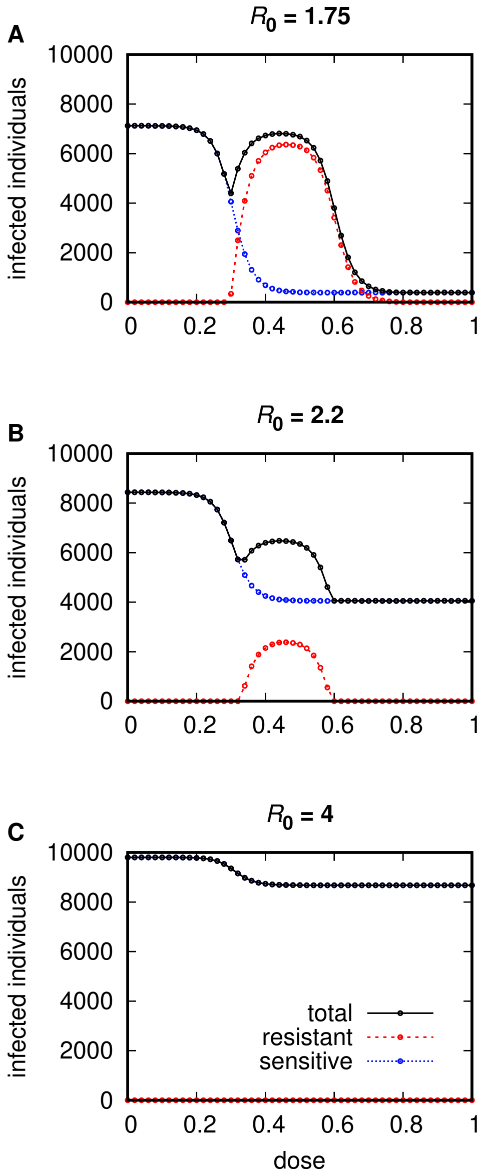

Figure XV. Number of infecteds in sequential epidemics caused by the sensitive and the resistant strains. Blue line: number of infected in the epidemic caused by the sensitive strain. Red line: number of infected in the subsequent epidemic caused by the resistant strain. Black line: sum of both. Simulations are shown for three values of the basic reproduction number R_0. Panel **A**: $R_0 = 1.75$, Panel **B**: $R_0 = 2.2$, Panel **C**: $R_0 = 4$. For $R_0 = 4$, the resistant strain cannot spread at all. The number of infected got determined at discrete points (denoted by the symbols), for which we had obtained simulation results for the recovery rates. Lines are included for readability.

recovery rate

$$\begin{aligned}\dot{S} &= -\beta S I \\ \dot{I} &= \beta S I - G(t) I \\ \dot{R} &= G(t) I.\end{aligned} \tag{S8.36}$$

The analytical solution of the SIR model and the exact value of the burden, even for a constant recovery rate, cannot be obtained explicitly. For a review of mathematical analysis of the SIR model, see [6]. In the next sections, we reduce the system of equations (S8.36) and make several approximations to formally show the presence of a valley and to determine the parameter regimes in which this valley exists.

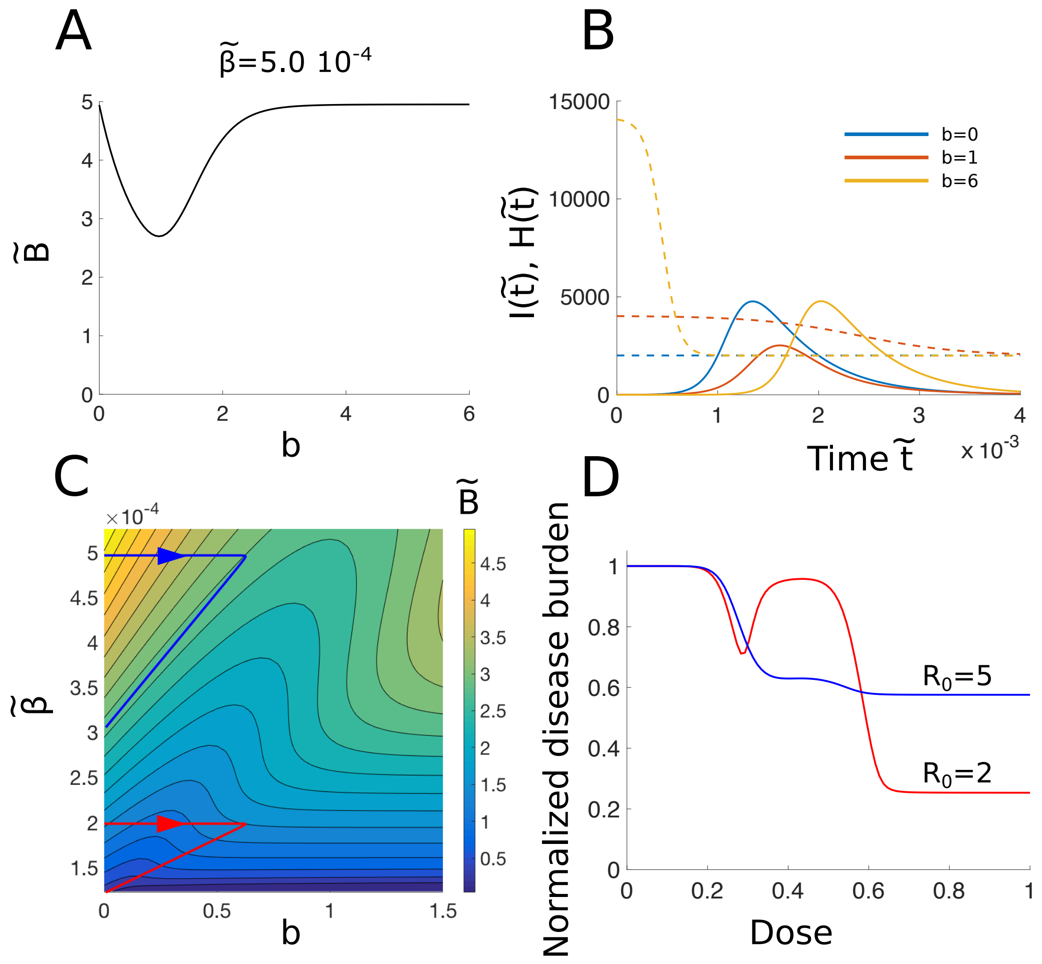

Figure XVI. Exploration of the conditions of existence of a valley in the disease burden (**A**) Disease burden $\tilde{B}$ (see Eq (S8.40)) as a function of b, for fixed values $\tilde{\beta} = 5.010^{-4}$ and $p_e = 2.010^{-4}$. (**B**) Number of infected individuals $I(\tilde{t})$ (continuous lines) and reduced recovery rate $H(\tilde{t})$ (dashed lines) as a function of time for $\tilde{\beta} = 5.010^{-4}$ and various values of b (in blue, $b = 0$, in red, $b = 1$ and in yellow, $b = 6$). (**C**) Surface plot of the reduced disease burden $\tilde{B}$ as a function of b and $\tilde{\beta}$. The black contours indicate regions of equal burden. The blue and red lines show how the value of the burden varies when increasing doses, for $R_0 = 2$ (red) and $R_0 = 5$ (blue). The arrow indicates increasing doses. (**D**) Normalized disease burden $B/B(\text{dose} = 0)$ as a function of the dose. Colors are the same as in panel (**C**).

S8.3.2 Reduction of the system of equations

We introduce the new variables $b = \frac{\gamma_S - \gamma_R}{\gamma_R}$, $\tilde{\beta} = \frac{\beta}{\gamma_R}$, $\tilde{t} = \beta t$. We first rewrite the function G (for $b \neq 0$ and $b \neq \frac{p_e}{1-p_e}$) and obtain

$$
\begin{aligned}
G(t) &= \gamma_R\Big((1-p_e)(b+1)\varphi(t) + (1-\varphi(t))\Big) \\
&= \gamma_R\Big(1 + (b + p_e(1+b))\varphi(t)\Big) \qquad \text{(S8.37)}
\end{aligned}
$$

We use the approximation

$$
G(t) = \gamma_R\Big(1 + b\varphi(t)\Big). \qquad \text{(S8.38)}
$$

This approximation is justified because p_e has little influence on the existence of a valley (Fig XIV). In particular, we showed that the disease burden has a very similar shape when we chose a small, constant p_e rather than a dose-dependent p_e. In the following, to explore the parameters that display or not a valley, we will therefore consider p_e to have a small constant value.

By dividing the equations of the system (S8.36) by γ_R, and with the change of time $\tilde{t}$, we obtain the system of equations

$$
\begin{aligned}
\frac{dS}{d\tilde{t}} &= -SI \\
\frac{dI}{d\tilde{t}} &= SI - H(\tilde{t})I \qquad \text{(S8.39)} \\
\frac{dR}{d\tilde{t}} &= H(\tilde{t})I,
\end{aligned}
$$

where $H(\tilde{t}) = \frac{1}{\beta}G(\beta t)$ is the reduced recovery rate. Note that H is independent of the variables of the system of equations S, I and R. It is given by

$$
H(\tilde{t}) = \begin{cases}
\frac{1}{\tilde{\beta}}\left(1 + \frac{1+p_e}{1-p_e} e^{-\frac{p_e}{\tilde{\beta}}\tilde{t}}\right) \text{ if } b = \frac{p_e}{1-p_e} \\[2ex]
\frac{1}{\tilde{\beta}}\left(1 + \frac{p_e}{1+\frac{p_e}{\tilde{\beta}}\tilde{t}}\right) \text{ if } b = 0 \\[2ex]
\frac{1}{\tilde{\beta}}\left(1 + \frac{b}{1+p_e\frac{b+1}{b}\left(e^{\frac{b}{\tilde{\beta}}\tilde{t}} - 1\right)}\right) \text{ otherwise.}
\end{cases}
$$

S8.3.3 Minimization of the burden

We now consider the reduced burden $\tilde{B}$, which we define as

$$
\begin{aligned}
\tilde{B} &= \int_0^\infty I(\tilde{t})d\tilde{t} \\
&= \beta\int_0^\infty I(t)dt \\
&= \beta B. \qquad \text{(S8.40)}
\end{aligned}
$$

When $\tilde{\beta}$ is fixed, there is a value of $b > 0$ that minimizes $\tilde{B}$ (provided that there is an outbreak). An illustration of this minimum is shown for $\tilde{\beta} = 5.010^{-4}$ in Fig XVIA. Finding b that minimizes the burden can be related to optimization problems in epidemiological models (see for instance [7]).

To understand why there is a minimum in the burden $\tilde{B}$, we examined in more detail the time course of I and of the reduced recovery rate H for various values of b (Fig XVIB). We find that, when $b = 0$, H is almost a constant equal to $\frac{1}{\tilde{\beta}}$. In this case the burden is the solution to the transcendental equation

$$\tilde{B} = \tilde{\beta} S_0 (1 - e^{-\tilde{B}}). \tag{S8.41}$$

In the case of large b, at the beginning of the outbreak the scaled recovery rate is $H(0) = \frac{1+b}{\tilde{\beta}}$, but decreases quickly to its asymptotic value $\frac{1}{\tilde{\beta}}$. In the beginning, the recovery rate of the infected sensitive to treatment is too large for the epidemics to spread in the population. The outbreak builds up slowly with a delay in the establishment of the epidemics which can be phrased in terms of R_0: at the beginning of the infection, R_0, which is proportional to $\frac{1}{H(0)}$, is therefore proportional to $\frac{1}{1+b}$, which is small. A large value of b corresponds to a small value of R_0. Later in the infection, the recovery rate is equal to $\frac{1}{\tilde{\beta}}$ and the burden is the same as in the case $b = 0$. Therefore, when the resistant strain first arises in the population, the pool of susceptible individuals is still almost untouched. The infection is almost the same as in the case $b = 0$, with a delay, due to the absence of the resistant strain at the beginning of the epidemic and its slow appearance through mutation (blue and yellow curves in Fig XVIB).

In the intermediate case $b = 1$, the overall epidemics is reduced. When b is not too large, the basic reproductive number of the sensitive infection is still large enough for it to spread, but at a lower rate than in the case $b = 0$. Before resistance can spread in the population, the number of susceptibles has dropped, reducing in turn the burden (cf. section S8.2).

This phenomenon can also be explained by observing the recovery rate H (dashed red curve in Fig XVIB): During the time course of the infection, the recovery rate is almost constant and larger than $\frac{1}{\tilde{\beta}}$. In the case of a constant recovery rate, the higher the recovery rate the smaller the burden (see Eq (S8.41) and [8]).

S8.3.4 When is there a valley?

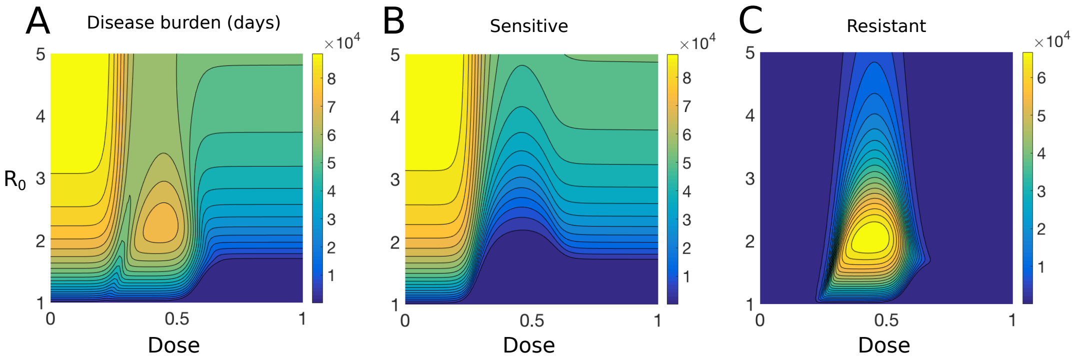

Figure XVII. Disease burden as a function of the dose and R_0. (**A**) Total burden $B = \int_0^\infty (I_S(t) + I_R(t))dt$. (**B**) Burden due to infections with the sensitive strain $B_S = \int_0^\infty I_S(t)dt$. (**C**) Burden due to infections with the resistant strain $B_R = \int_0^\infty I_R(t)dt$.

To look more systematically at the effect of the parameters b and $\tilde{\beta}$ on the burden $\tilde{B}$, we represented it as a surface plot (Fig XVIC).

We show the disease burden for varying dose and $R_0 = 2$ and $R_0 = 5$. Varying the dose is equivalent to changing the ratio $b = \frac{\gamma_S}{\gamma_R} - 1$, but also the normalized transmission coefficient $\tilde{\beta} = \frac{\beta}{\gamma_R}$. On the surface plot, varying the dose corresponds first to a horizontal line (little variation of γ_R with the dose, but large variations of γ_S), and for larger dosage the difference

in recovery rates reduces. Thus, b becomes smaller, and $\tilde{\beta}$ also decreases due to the increase in γ_R.

We observe that at $R_0 = 2$ and $R_0 = 5$, the regions explored for various doses are very different. For $R_0 = 2$, the disease burden first decreases, then increases, in a region where $\tilde{\beta}$ is almost constant, and then decreases with decreasing b. These variations in the burden correspond to the existence of a valley.

For $R_0 = 5$, the disease burden almost continuously decreases. If b would have taken larger values, it would have been possible to observe an increase in the burden even for such high R_0. This is in agreement with the predictions of section S8.2: we see that it is possible to observe a valley even for large values of R_0, provided that the difference in recovery rates is large enough. To visualize more clearly the different behaviors of the burden, we also represent the (non reduced) burden B as a function of the dose (Fig XVID) for $R_0 = 2$ and $R_0 = 5$.

To conclude, we showed that the most important parameters for the existence of a valley in the disease burden are the values explored by β/γ_R and the range of values taken by the ratio γ_S/γ_R when varying the dose.

Other parameters, such as p_e, certainly have an influence on the position of the local minimum in the disease burden but little influence on the existence of a valley. Indeed, taking a constant value for p_e instead of the inverted U-shape shows little to no changes in the disease burden (Fig XIV, blue and purple curves).

S9: Sensitivity analysis

We conducted a sensitivity analysis to determine how the recovery rates and the resistance probability of emergence varied with changes of one parameter. For this, we ran stochastic simulation of the within-host model. The sigmoidal shape of the recovery rates and the inverted U-shape of the probability of emergence are conserved in the range of explored parameters (Fig XVIII, XIX and XX). (Only for extreme parameter choices for the immune response do the recovery rates turn non-monotonic; we excluded these parameters since they lead to very high probabilities of within-host resistance and do not appear relevant. If the MIC of the two strains is very similar, the within-host probability of resistance becomes monotonically decreasing; again, this is situation is not very relevant.) What is more, the location of the peak in the within-host probability of resistance proves to be robust. It corresponds to doses where spread of resistance is easiest.

In the main text, we defined the probability of within-host resistance as the probability that the number of resistant pathogens crosses a threshold of 50. If we change the criterion for the emergence of resistance to 100 pathogens as in [9], the probability of within-host resistance is lower but the location of the peak remains the same (Fig XXI). It is again robust to variations in single parameters (Fig XXII).

At the population level, we also varied parameters (the transmission rate and the population size). We chose a subset of within-host parameters and performed stochastic simulations of the nested model to determine how the outbreak probability, the number of resistant strain transmission events, and the disease burden varied with dosage if we vary one of these within-host parameters (Fig XXIII- XXV).

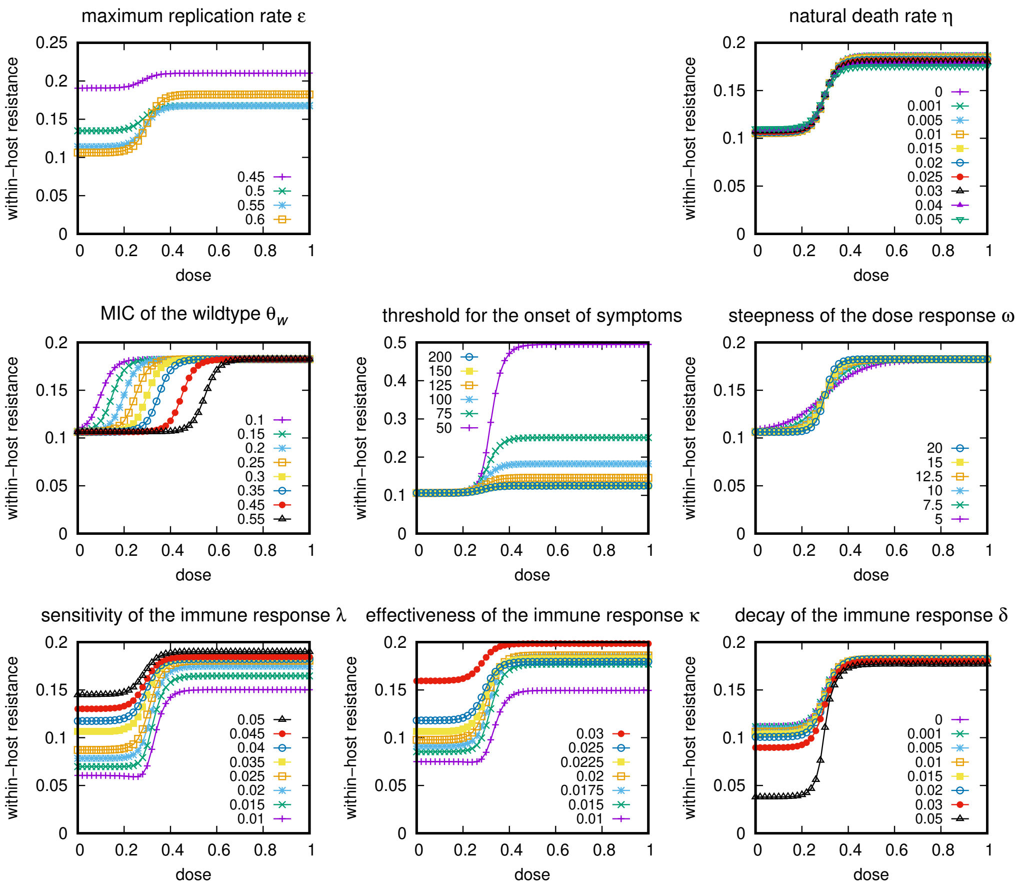

Figure XVIII. Sensitivity analysis exploring the recovery rate of the wild-type strain in stochastic simulations. In each panel, we vary one parameter with respect to the parameter set used in the main text.

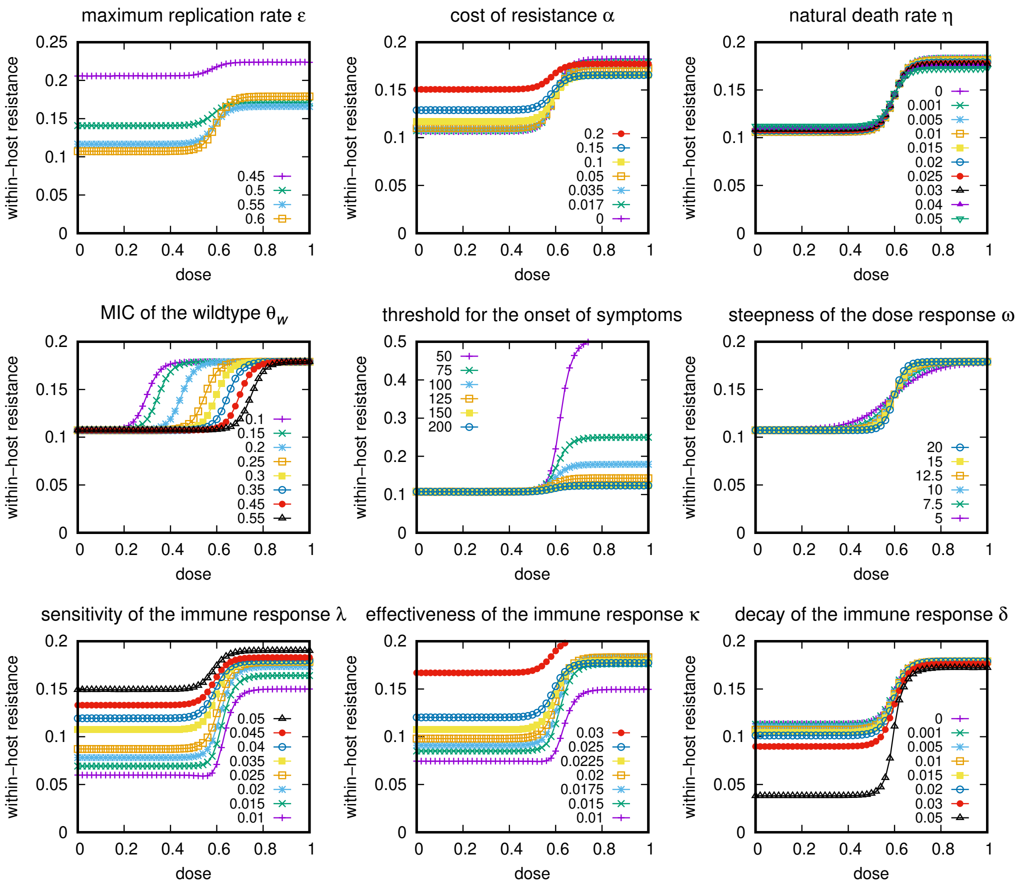

Figure XIX. Sensitivity analysis of the recovery rate of the resistant strain. In each panel, we vary one parameter with respect to the parameter set used in the main text.

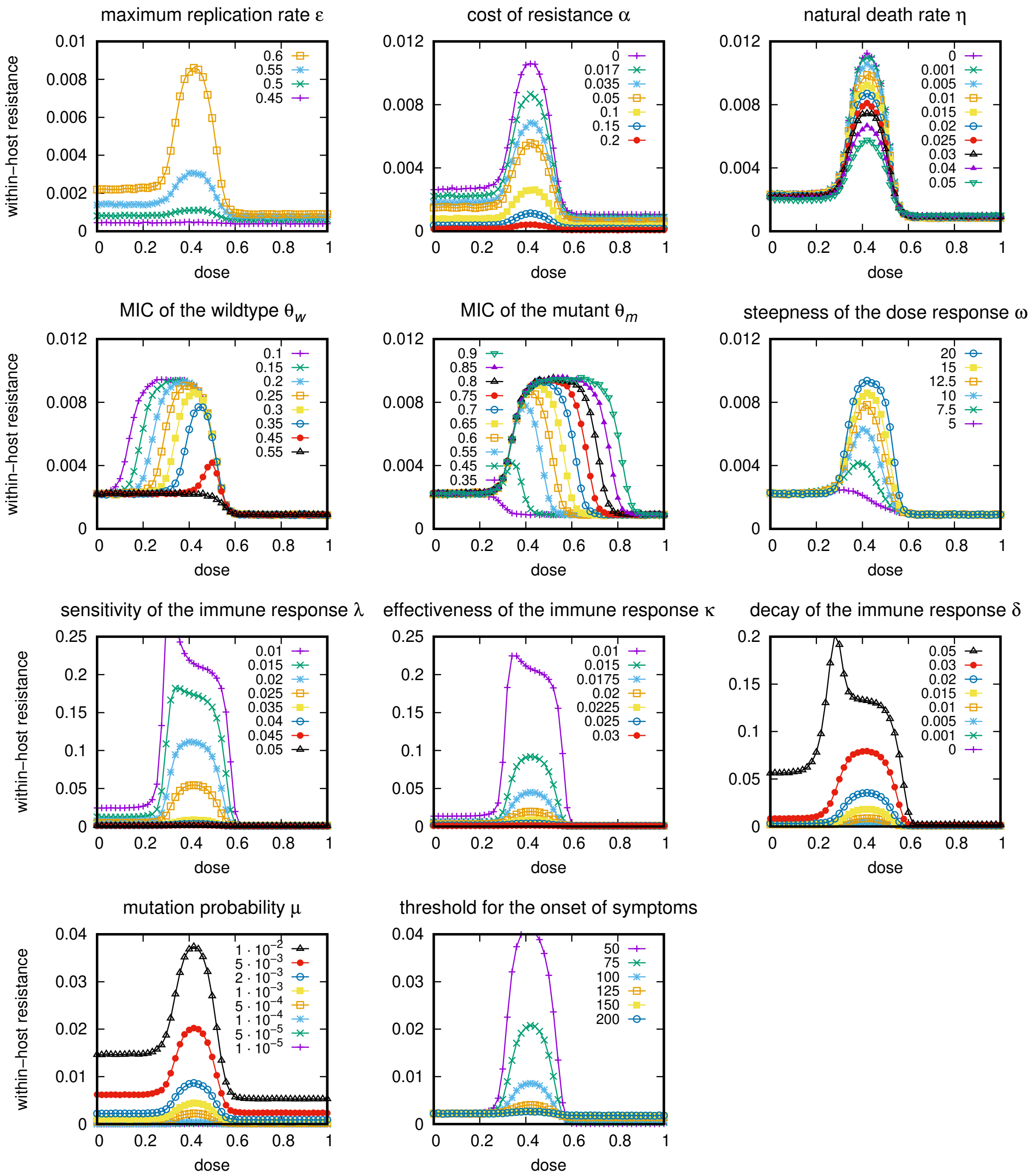

Figure XX. Sensitivity analysis of the probability of emergence of resistance at the within-host scale. In each panel, we vary one parameter with respect to the parameter set used in the main text.

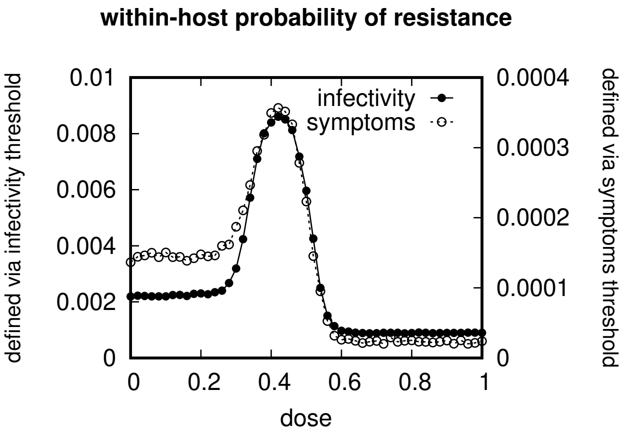

Figure XXI. Comparison of the within-host probability of resistance if it is defined as the number of resistant pathogens crossing (1) the infectivity threshold of 50 pathogens (filled circles) or (2) the symptoms threshold of 100 pathogens (empty circles).

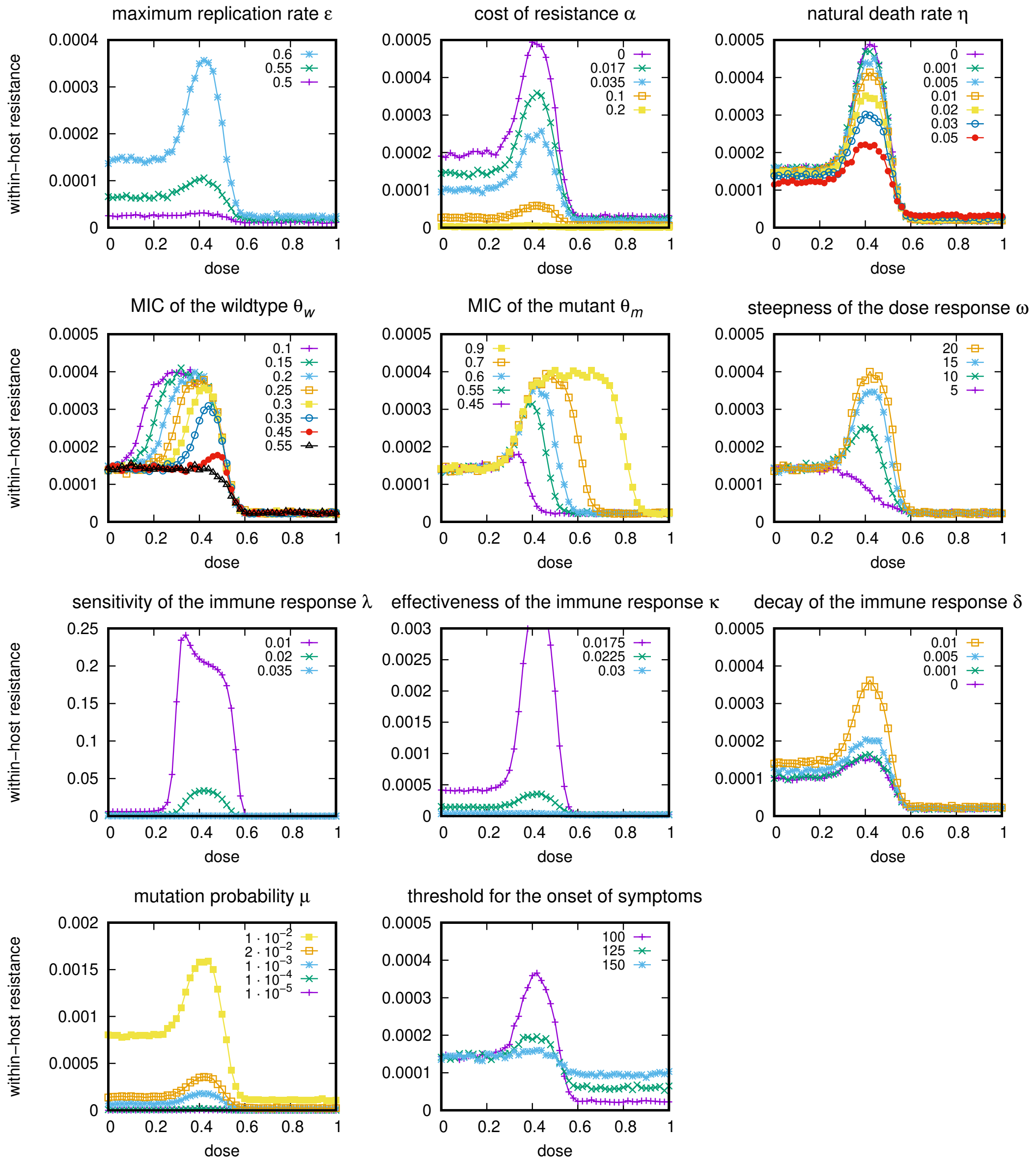

Figure XXII. Sensitivity analysis of the probability of emergence of resistance at the within-host scale if we define the emergence of resistance as the number of resistant pathogens reaching 100. In each panel, we vary one parameter with respect to the parameter set used in the main text.

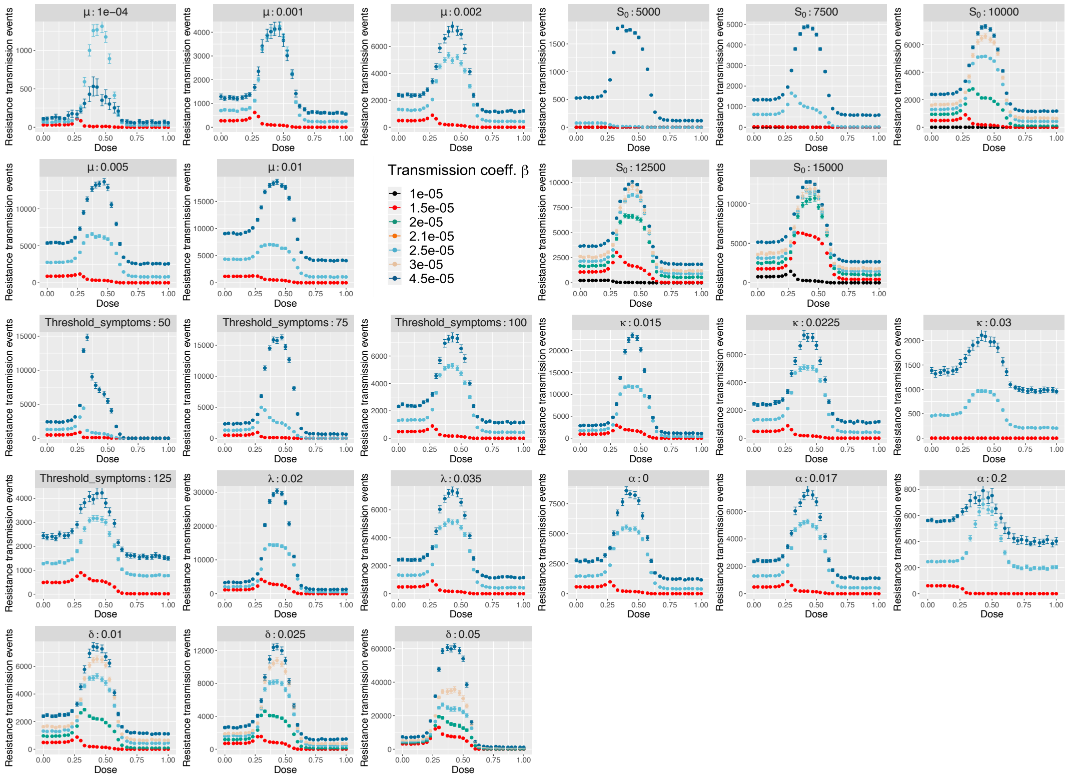

Figure XXIII. Sensitivity analysis of the number of resistant strain transmission events. The influence of several within-host parameters (δ, α, κ, λ, μ, threshold for the appearance of symptoms) and the population parameters β and S_0 were tested.

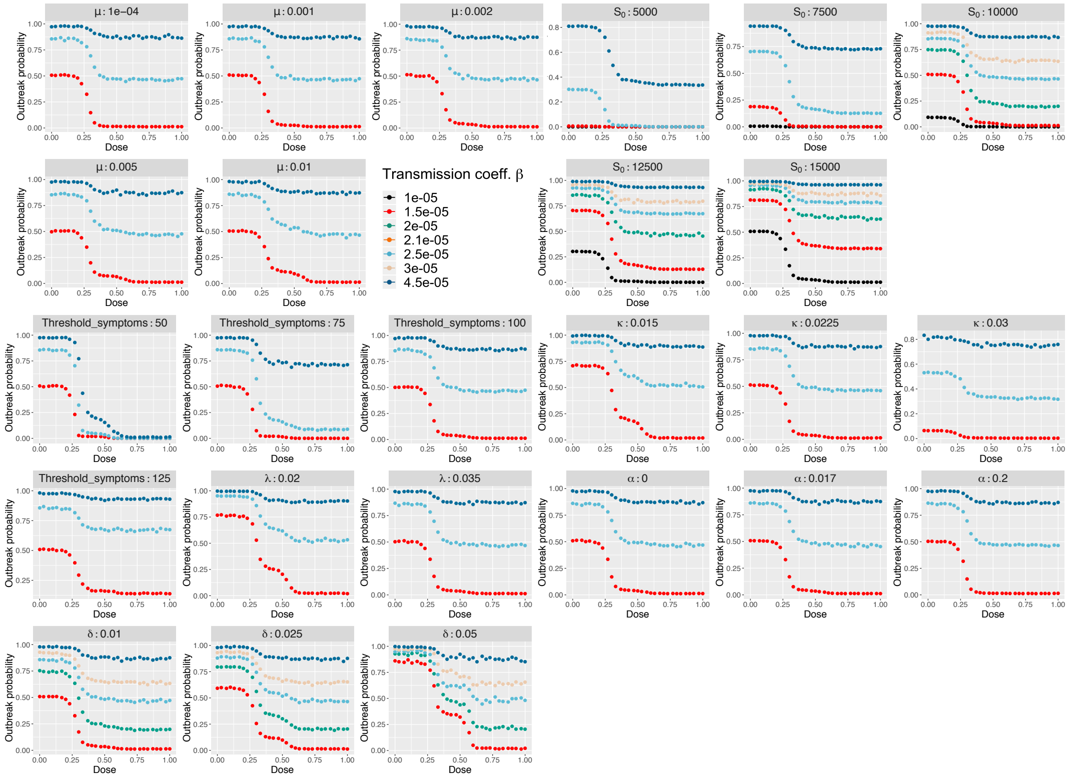

Figure XXIV. Sensitivity analysis of the outbreak probability. The influence of several within-host parameters (δ, α, κ, λ, μ, threshold for the appearance of symptoms) and the population parameters β and S_0 were tested.

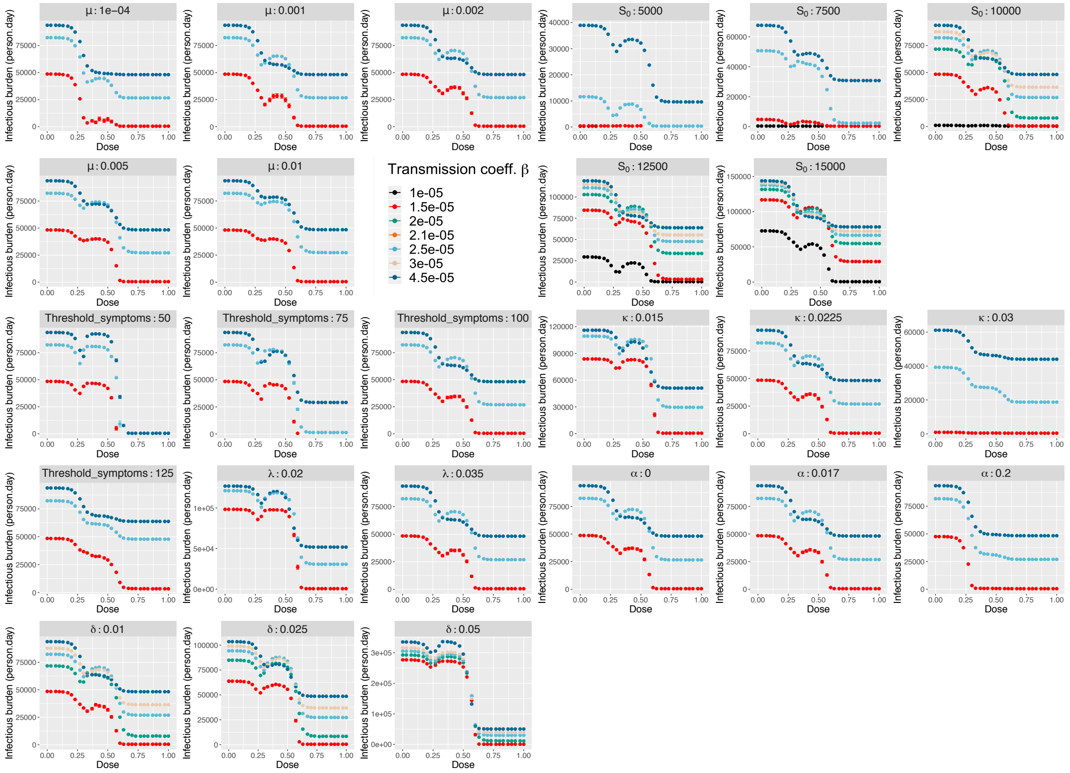

Figure XXV. Sensitivity analysis of the infectious disease burden. The influence of several within-host parameters (δ, α, κ, λ, μ, threshold for the appearance of symptoms) and the population parameters β and S_0 were tested.

References

[1] Sewastjanow BA. Verzweigungsprozesse. Berlin: Akademie-Verlag; 1974.

[2] Allen LJS. An Introduction to Stochastic Epidemic Models. In: Brauer F, van den Driessche P, Wu J, editors. Mathematical Epidemiology. Berlin, Heidelberg: Springer; 2008.

[3] Handel A, Longini Jr IM, Antia R. What is the best control strategy for multiple infectious disease outbreaks? Proceedings of the Royal Society B. 2007;274:833–837.

[4] Brauer F. Compartmental Models in Epidemiology. In: Brauer F, van den Driessche P, Wu J, editors. Mathematical Epidemiology. Berlin, Heidelberg: Springer; 2008.

[5] Lipsitch M, Cohen T, Murray M, Levin BR. Antiviral resistance and the control of pandemic influenza. PLoS Medicine. 2007;4(1):e15.

[6] Hethcote HW. The mathematics of infectious diseases. SIAM review. 2000;42(4):599–653.

[7] Hansen E, Day T. Optimal control of epidemics with limited resources. Journal of Mathematical Biology. 2011;62(3):423–451.

[8] Weiss HH. The SIR model and the Foundations of Public Health. MATerials MATemàtics. 2013; p. 1–17.

[9] Day T, Read AF. Does High-Dose Antimicrobial Chemotherapy Prevent the Evolution of Resistance? PLoS Computational Biology. 2016;12(1). doi:10.1371/journal.pcbi.1004689.
